# Interactions between curcumin and human salt-induced kinase 3 elucidated from computational tools and experimental methods

**DOI:** 10.3389/fphar.2023.1116098

**Published:** 2023-04-13

**Authors:** Mingsong Shi, Yan Zhou, Haoche Wei, Xinyu Zhang, Meng Du, Yanting Zhou, Yuan Yin, Xinghui Li, Xinyi Tang, Liang Sun, Dingguo Xu, Xiaoan Li

**Affiliations:** ^1^ NHC Key Laboratory of Nuclear Technology Medical Transformation, Mianyang Central Hospital, School of Medicine, University of Electronic Science and Technology of China, Mianyang, Sichuan, China; ^2^ State Key Laboratory of Biotherapy/Collaborative Innovation Center of Biotherapy and Cancer Center, West China Hospital, Sichuan University, Chengdu, Sichuan, China; ^3^ West China School of Pharmacy, Sichuan University, Chengdu, Sichuan, China; ^4^ College of Chemistry, MOE Key Laboratory of Green Chemistry and Technology, Sichuan University, Chengdu, Sichuan, China; ^5^ Key Laboratory of Basic Pharmacology of Ministry of Education and Joint International Research Laboratory of Ethnocentric of Ministry of Education, Zunyi Medical University, Zunyi, Guizhou, China; ^6^ Shenzhen Shuli Tech Co., Ltd, Shenzhen, Guangdong, China; ^7^ Research Center for Material Genome Engineering, Sichuan University, Chengdu, Sichuan, China

**Keywords:** curcumin, salt-induced kinase, molecular dynamics simulation, molecular docking, binding free energy, binding model

## Abstract

Natural products are widely used for treating mitochondrial dysfunction-related diseases and cancers. Curcumin, a well-known natural product, can be potentially used to treat cancer. Human salt-induced kinase 3 (SIK3) is one of the target proteins for curcumin. However, the interactions between curcumin and human SIK3 have not yet been investigated in detail. In this study, we studied the binding models for the interactions between curcumin and human SIK3 using computational tools such as homology modeling, molecular docking, molecular dynamics simulations, and binding free energy calculations. The open activity loop conformation of SIK3 with the ketoenol form of curcumin was the optimal binding model. The I72, V80, A93, Y144, A145, and L195 residues played a key role for curcumin binding with human SIK3. The interactions between curcumin and human SIK3 were also investigated using the kinase assay. Moreover, curcumin exhibited an IC_50_ (half-maximal inhibitory concentration) value of 131 nM, and it showed significant antiproliferative activities of 9.62 ± 0.33 µM and 72.37 ± 0.37 µM against the MCF-7 and MDA-MB-23 cell lines, respectively. This study provides detailed information on the binding of curcumin with human SIK3 and may facilitate the design of novel salt-inducible kinases inhibitors.

## 1 Introduction

Mitochondrion plays indispensable role in programmed cell death, redox signaling, and energy metabolism, which suggests mitochondrion is a favorable target (Indran et al., 2011). Direct targeting mitochondria improves antiproliferative activity and decreases cancer invasion (Millard et al., 2013; Ye et al., 2017; Pan et al., 2018). In addition, mitochondria-targeted cytotoxic conjugates, which was combined one parent drug to mitochondria-directing compound with linker, are one the important approaches for targeting therapeutics (Zorova et al., 2018; Jeena et al., 2020). For example, the natural drug honokiol linked with the effective mitochondria-directing compound berberine were designed and synthesized a mitochondria-targeted conjugates (Shi et al., 2020). Those derivatives for honokiol shown effective antitumor activity than honokiol. Thus, based on natural mitochondria-targeted cytotoxic conjugates is one interesting field for cancer therapeutic.

**FIGURE 1:**
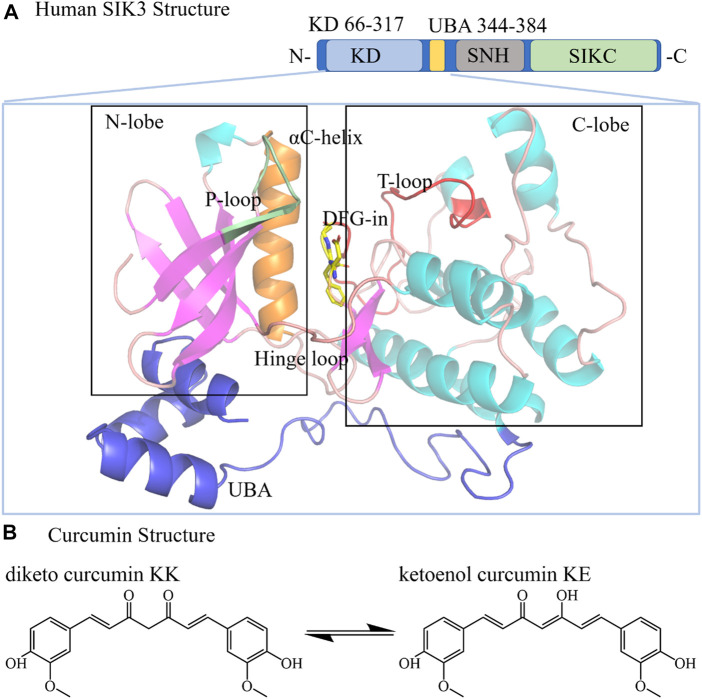
Structures of human SIK3 and curcumin. **(A)** Structure of human SIK3. Human SIK3 (UniProt ID: Q9Y2K2, residues: 1–1321) formed from a kinase domain (KD, residues: 66–317), central SNF protein kinase homology domain (SNH), and phosphorylation domain near the C-terminal (SIKC). The UBA domain (residues: 344–384) is a linker between KD and SNH. The kinase domain includes an N-terminal region (N-lobe), a C-terminal region (C-lobe), and a hinge loop between the N-lobe and C-lobe. The αC-helix, DFG motif, T-loop, and P-loop also play important roles in protein kinase activity. The ATP (adenosine triphosphate)-binding pocket (active pocket) was formed between the N-lobe and C-lobe. **(B)** Structure of the diketo (KK) and ketoenol (KE) forms of curcumin.

Natural products have many notable characteristics, such as wide efficacy and low toxicity; they have recently attracted increasing research attention as lead compounds to treat mitochondrial dysfunction-related diseases, cancer, and antimicrobial-resistant infections (Du et al., 2021; An et al., 2022; Rahman M. A. et al., 2022; Bernal et al., 2022; Rahman M. M. et al., 2022; He et al., 2022; Liao et al., 2022; Nurumal et al., 2022; Su et al., 2022; Xu et al., 2022). Especially, the effects of many natural product on mitochondrial dysfunction-related diseases have been recently studied (An et al., 2022; Rahman M. A. et al., 2022; Rahman M. M. et al., 2022). Particularly, curcumin (bis(4-hydroxy-3-methoxyphenyl)-1,6-heptadiene-3,5-dione, CAS No. 458-37-7, Figure 1), a natural dietary polyphenol compound, has played close attention to its anti-inflammatory, antioxidant, neuroprotective, and antitumor activities (Golonko et al., 2019; Dev et al., 2021; Lei et al., 2022). Curcumin also induces paraptosis in breast cancer cell lines from swelling and fusion of mitochondria and endoplasmic reticulum (Yoon et al., 2010). Curcumin is a natural product which has been extracted from the turmeric, zedoary, and other ginger plants. Notably, it is the main bioactive compound in turmeric. Curcumin is mainly used for treating cancer. (Rajendran et al., 2022; Tang et al., 2022; Vadukoot et al., 2022; Wang et al., 2022; Yin et al., 2022). However, the anticancer activity of curcumin has not been fully elucidated *in vivo* and *in vitro*.

The mechanism for the antitumor activity of natural compound curcumin were related with multiple signaling pathways, such as the signal transducer, nuclear factor-κB, cyclooxygenase 2, mitogen-activated protein kinase, and activator of transcription 3, and TNF-α signaling pathways. Curcumin is a spectrum inhibitor that targets epidermal growth factor receptor (Gopal, 2022), protein kinase C (Hasmeda and Polya, 1996), tubulin (Chakraborti et al., 2011; Ota et al., 2018), cyclin-dependent kinase 2 (Sumirtanurdin et al., 2020), casein kinase 2 (Cozza et al., 2020), adenosine monophosphate-activated protein kinases (Soltani et al., 2019), and Abelson leukemia virus tyrosine kinase 1 (Rodrigues et al., 2021). A previous study reported that curcumin exhibits antitumor activity in triple-negative breast cancer patient-derived xenograft tumor mice by inhibiting the expression of salt-inducible kinase 3 (SIK3) (Cheng et al., 2021). However, the interactions between curcumin and SIK3 remain largely unclear, which hinders its use as a lead compound to design salt-inducible kinases inhibitors.

Salt-inducible kinases (SIKs) control the anti-inflammatory cytokine interleukin-10 (IL-10) in macrophages which is one of the cyclic adenosine monophosphate-dependent production (Okamoto et al., 2004; Du et al., 2016; Sakamoto et al., 2018; Wein et al., 2018; Chen F. Y. et al., 2019; Sun et al., 2020). Three isoforms exist for the SIKs family: SIK1, SIK2, and SIK3 (Katoh et al., 2004). SIKs are ubiquitously expressed and control the gene expression for increasing the intracellular levels of cyclic adenosine monophosphate. They are also found to regulate lipid and energy metabolism (Du et al., 2008; Sun et al., 2020). Moreover, SIKs inhibition can be considered as a therapeutic strategy for inflammatory disease (Antonio et al., 2022). In addition, SIKs are also dysregulated in prostate cancer, ovarian cancer, lung cancer, breast cancer, and other cancers (Du et al., 2016; Wein et al., 2018; Chen F. Y. et al., 2019), which indicates that SIKs are appealing pharmacological targets for treating cancer. Recently, numerous SIKs inhibitors have been developed and applied in the preclinical or clinical studies (Heap et al., 2017; Jin et al., 2020), such as HG-9-91-01 (Clark et al., 2012), KIN112 (Clark et al., 2012), YKL-06-062 (Mujahid et al., 2017), MRT67307, MRT199665, OMX-0407 (Hartl et al., 2021), and ARN-3236 (Supplementary Figure S1) (Zhou et al., 2017). However, no specifically SIKs inhibitors have been clinically approved. Thus, novel inhibitors specifically targeting SIKs is need to develop.

Curcumin inhibits the activity of SIK3 (Cheng et al., 2021), which suggests that it can be used as a lead compound for the optimization of SIKs inhibitors. However, reliable curcumin-SIKs structures have not yet been reported. The detailed structural information on drug–target binding plays a significant important role in computer–aided drug design. The ligand-receptor interaction is one of the incorporations to elucidate the drug–target binding. The target structure can be obtained from crystal structure or computational modeling. The homology modeling (HM) as one widely applied method can be used to predict target structures, which is based on amino acid sequences (Wieman et al., 2004; Xiang, 2006; Cavasotto and Phatak, 2009; Cavasotto, 2011; Tuccinardi and Martinelli, 2011; Gadhe et al., 2015; Munsamy and Soliman, 2017; Salam et al., 2018; Muhammed and Aki-Yalcin, 2019). The molecular docking method is employed to construct drug-target complexes for drug design (Hirayama, 2007; Kroemer, 2007; Bello et al., 2013; Wong, 2015; Scotti et al., 2017; Amaro et al., 2018; Pinzi and Rastelli, 2019; Saikia and Bordoloi, 2019; Jakhar et al., 2020). Molecular dynamics (MD) simulations have been applied to learn the interactions between inhibitors and proteins (Rognan, 1998; Durrant and McCammon, 2011; Aci-Seche et al., 2016; Hernandez-Rodriguez et al., 2016; Do et al., 2018; Liu et al., 2018; Yamashita, 2018; Bera and Payghan, 2019; Zou et al., 2019).

In this study, we obtained the curcumin–SIK3 binding models using homology modeling, molecular docking, and molecular dynamics simulation. We identified that the I72, V80, A93, Y144, A145, and L195 residues of curcumin showed antitumor activity. Moreover, curcumin inhibited human SIK3 protein kinase with an IC_50_ value (half maximal inhibitory concentration) of 131 nM. The antitumor activity of curcumin was also tested in human breast cancer cell line MDA-MB-231 and MCF-7. To the best of our knowledge, our study had identified the complexes for curcumin binding with human SIK3 based on computational analysis and experimental methods.

## 2 Materials and methods

### 2.1 Kinase assay

The IC_50_ values of curcumin against human SIK3 were determined by KinaseProfiler radiometric protein kinase assays obtained from Eurofins Pharma Discovery Services UK Limited (Eurofins). The adenosine triphosphate (ATP) concentrations used here represent the *K*_m_ values of the corresponding kinases.

### 2.2 Cell assay

Human breast cancer cell line MDA-MB-231 and MCF-7 were obtained from the American Type Culture Collection (ATCC; Manassas, Virginia, United States). The cells were cultured in Dulbecco’s modified Eagle’s medium (DMEM, for MDA-MB-231) or minimum essential medium (MEM, for MCF-7), which was supplemented with 1% Penicillin-Streptomycin (PS) and 10% Fetal bovine serum (FBS). Approximate 4 × 10^3^ cells, the cells were plated into each well in 96-well plate and were incubated in 5% CO_2_ at 37°C for 24 h. Curcumin (MDL No. MFCD00008365, CAS No. 458-37-7, purity 98%) was purchased from Shanghai Macklin Biochemical Co., Ltd. (Shanghai, China). HG-9-91-01 (TargeMol No. T4599, CAS No. 1456858-58-4, purity 99.64%) was purchased form TargetMol (TargetMol Chemicals Inc., Boston, United States). The tested compounds at the indicated final concentrations were added to the culture medium and incubated for 72 h (3-(4,5-dimethylthiazol-2-yl)-2,5-diphenyl-2H-tetrazolium bromide) (MTT) was added in each cell. Subsequently, the cells were incubated for an additional 2 h in each well which were dissolved in 100 μL dimethyl sulfoxide (DMSO). The absorbance values (OD) of the 96-well tissue culture plates were read at 570 nm on a Spectra MAX M5 microplate spectrophotometer. The cell viability results were calculated using the GraphPad Prism 6.0 software. This assay was repeated three times.

### 2.3 SIK3 structure preparation

The protein sequence of human SIK3 (UniProt ID: Q9Y2K2, 1,321 residues) was downloaded from the UniProt database (Bateman et al., 2015; Bateman et al., 2017; Bateman et al., 2019). Experimentally, most SIKs inhibitors bind to the ATP-competitive inhibitors with bind into the ATP-binding pocket (Zhou et al., 2017; Liu et al., 2021; Tesch et al., 2021). Moreover, curcumin binds to the ATP-binding site of human DYRK2 [PDB ID: 6HDR (Elkins and Knapp, 2019)]. Thus, we speculated that curcumin may also bind to the ATP-binding site of human SIK3. The T-loop (also named activation loop or A-loop)) conformation of SIK2 had two class conformation with closed and open conformation, and so on the two class conformations of T-loop for SIK3 also presented. To construct the two conformations of SIK3 structure, two separate homology modeling experiments were run from the online service SWISS-MODEL (Waterhouse et al., 2018). Although the full sequence of human SIK3 contains three main domains, we only considered the protein kinase domain and ubiquitin-associated (UBA) domain (residues: 1–390) in this study. The crystal structure of catalytic and UBA domain of rat MARK2 [PDB ID: 2WZJ (Marx et al., 2010)] was employed to model the closed T-loop conformation for human SIK3 (labeled as SIK3-C). Meanwhile, kinase and UBA domain of human MARK2 [PDB ID: 3IEC (Nesic et al., 2010)] was used to construct the structure of the open conformation of T-loop for human SIK3 (defined as SIK3-O). Both SIK3 and MARK2 have similar structures and functions which results from belonging to the calcium/calmodulin-dependent protein kinase-like family (Gormand et al., 2011; Alexander et al., 2019). The VERIFY3D software (Bowie et al., 1991), ERRAT (Colovos and Yeates, 1993), the GMQE method (Benkert et al., 2011), and a Ramachandran plot obtained by the ProCheck software (Laskowski et al., 1993) were applied to validate the modeling SIK3 structure SIK3-C and SIK3-O.

### 2.4 Curcumin structure preparation

The two tautomers of curcumin, namely the diketo (KK) and ketoenol forms (KE), were constructed via the In Draw software (Integle chemical draw). The molecular structures of the two form curcumin KK and KE were optimized at the B3LYP/6-311G (d, p) level in the gas phase (Hohenberg and Kohn, 1964; Kohn and Sham, 1965; Becke, 1988; Lee et al., 1988; Miehlich et al., 1989; Blaudeau et al., 1997; Rassolov et al., 1998; Tran et al., 1999; Rassolov et al., 2001). After the optimization, frequency calculations were also performed at the B3LYP/6-311G (d, p) level. There are no imaginary frequencies for the optimization steps which indicate that those optimized structures for KK and KE curcumin are stable. All calculations were performed with the Gaussian 09 software package (Frisch et al., 2009).

### 2.5 Molecular docking

Currently, no crystalline structures have been reported for the binding of curcumin to human SIK3. Additionally, the T-loop conformation of SIK3 of curcumin during its binding is also unclear. Therefore, in this study, both the open and closed T-loop conformations (SIK3-C and SIK3-O) were considered. In addition, the two forms curcumin (KK and KE) were also applied to construct the curcumin-SIK3 complex structures. KK or KE form curcumins with an open or closed conformation of the T-loop (SIK3-C and SIK3-O) were employed to construct the curcumin/SIK3 complexes. PYMOL 2.1 (Schrödinger, 2010) was employed to prepare the receptor structure of human SIK3 based on the HM results of SIK3-C and SIK3-O. Subsequently, receptor protein SIK3 and ligand small molecule curcumin were pretreated by AutoDockTools 1.5.6, (Sanner, 1999), although three steps: 1) added hydrogen atoms; 2) adding Gasteiger charges (Gasteiger and Marsili, 1980); 3) adjusted the unreasonable atomic overlap. Subsequently, a grid box was estimated by AutoGrid v.4.2 (Morris et al., 2009) with a 0.375 Å grid spacing and a 40 × 40 × 40 grid size. The center of the ATP-binding pocket of human SIK3 was defined as the center of the grid box. Finally, 2000 conformations were constructed by the Lamarckian genetic algorithm (Fuhrmann et al., 2010) for the four systems (SIK3-C-KK, SIK3-C-KE, SIK3-O-KK, and SIK3-O-KE) in AutoDock v.4.2 (Morris et al., 2009). The conformation with the best rational orientation in the ATP-binding pocket, which was based on the curcumin with DYRK2 crystal structure (such as the key hydrogen bonds between curcumin and the hinge loop of DYRK2, PDB ID: 6HDR), was selected as the optimal conformation.

### 2.6 Molecular dynamics (MD) simulation

The four complex curcumin/SIK3 systems, SIK3-C-KK, SIK3-C-KE, SIK3-O-KK, and SIK3-O-KE, which were obtained from molecular docking, were run in molecular dynamics simulations to study the binding models. Thus far, no standard force field parameters have been generated for the KE and KK forms of curcumin. Therefore, the restrained electrostatic potential protocol (Bayly et al., 1993) and the general amber force field (GAFF, version 2) (Wang et al., 2004) were employed to generate the force field parameters for the KK and KE forms of curcumin with the Antechamber module in AMBERTools21 (Case et al., 2020). The partical aomic charges of KK and KE curcumin were obtained with HF/6-31G theory level. In addition, the standard protein force field [AMBER ff19SB force field (Tian et al., 2020)] was applied to generate the human SIK3 topology parameters. The protonation state of human SIK3 was determined with pH of 7.4 using the H++ online service (Bashford and Karplus, 1990; Gordon et al., 2005; Myers et al., 2006; Anandakrishnan et al., 2012). One Na^+^ ion was required to neutralize the curcumin/SIK3 complex systems. TIP3P water (Jorgensen et al., 1983) was used to solvate the complex systems in a 15 Å cuboid water box. Subsequently, 20,067, 20,065, 19,677, and 19,676 waters were added for SIK3-C-KK, SIK3-C-KE, SIK3-O-KK, and SIK3-O-KE, respectively. Lastly, the curcumin/SIK3 complex systems included 320 residues of human SIK3, one small molecule of curcumin, one Na^+^ ion, and approximately 19,670–20,100 water molecules. Firstly, the curcumin/SIK3 complex system was subjected to optimize with steepest descent method (9,000 steps) and the conjugate gradient method (1,000 steps) that all the solute molecules were fixed and not considered to minimize. Secondly, conjugate gradient method (10,000-step) was employed to minimize the entire system without restriction. After the two minimization steps, Langevin dynamics were employed to increase the temperature of the complex system from 0 to 300 K in 200 ps. The pressure was then maintained by isotropic position scaling at 1 bar for 200 ps. Subsequently, the system was pre-equilibrated within the NPT (isothermal-isobaric) ensemble at 300 K and 1 bar in 200 ps. Finally, the entire system underwent 500 ns MD simulations for data collection and analyses. In this work, all MD simulations were run with AMBER20 and AMBERTools21 (Case et al., 2020). The *CPPTRAJ* module (Roe and Cheatham, 2018; Cheatham et al., 2019) was then employed to analyze the data.

### 2.7 Binding free energy estimation

The MM/GBSA approach (Srinivasan et al., 1998; Lee et al., 2004) was used to calculate the binding free energies between curcumin and human SIK3. The MM/GBSA method has been widely employed to evaluate the binding affinities between ligands and enzyme (Tse and Verkhivker, 2015; Wang et al., 2017; Chen Q. Q. et al., 2019; Shi and Xu, 2019; Wei et al., 2019; Xu and Zheng, 2022). Previously, the MM/GBSA framework has been detailly introduced (Honig and Nicholls, 1995; Genheden and Ryde, 2015; Onufriev and Case, 2019). For the four curcumin/SIK3 complex systems, the MM/GBSA approach was employed to calculate the energy terms over 1,000 frames from the last 200 ns of the MD simulation. In addition, the entropy was obtained via the statistical average with 100 frames for 2 ns interval. In order to determine the contributions of each residue, the total binding energy between SIK3 and curcumin was decomposed with the MM/GBSA binding energy decomposition without considering the entropies (Gaillard and Simonson, 2014). The energy was then calculated using the MMPBA. py program in AMBERTools21 (Case et al., 2020). More information about the binding free energy calculation is provided in the Supplementary Material. A scheme of the methods employed is shown in Supplementary Figure S2.

## 3 Results and discussion

### 3.1 SIK3 structure modelling

Generally, the SIK3 protein comprises three domains (Figure 1): a serine-threonine kinase domain (KD) near the N-terminal, a central sucrose non-fermenting protein kinase homology domain (SNF), and a long phosphorylation domain (SIKC) near the C-terminal (Katoh et al., 2004; Antonio et al., 2022). A ubiquitin-associated (UBA) region followed by KD has also been defined. Specifically, the kinase domain phosphorylated threonine residues on the substrate proteins regulate downstream genes, such as CREB, class IIa HDAC, and PME-1 (Okamoto et al., 2004; Taub et al., 2010; Du et al., 2016; Wein et al., 2018; Chen F. Y. et al., 2019; Taub, 2019; Sun et al., 2020; Antonio et al., 2022). In this study, the kinase domain (residues: 66–317) and UBA region (344–384) were considered for following structural construction (residues: 1–400, labeled as SIK3 in the following text), and SNF and SIKC were excluded.

The three-dimensional structure of the human SIK3 kinase domain is composed of an N-terminal region (labeled as N-lobe) and a C-terminal region (defined as C-lobe). The N-lobe consists of five stranded β-sheets (named as β1–β5) and one α-helix (labeled as αC helix). The C-lobe is mostly α-helical (αD–αI) but contains important β-sheets (β6–β7). The hinge loop for protein kinase is referred for the linker between the C- and N-lobes (Supplementary Figure S3) (Hanks and Hunter, 1995; Cherry and Williams, 2004). The ATP-binding site is formed from the pocket between the N-lobe and C-lobe. In protein kinases, the DFG (Asp-Phe-Gly) motif in the C-lobe is highly conserved and follows T-loop. The T-loop serves as a key regulating region for protein kinase activities (Canagarajah et al., 1997; Stevenson et al., 2002; Cha et al., 2015; Mansueto et al., 2019). The activation of protein kinases depends considerably on the DFG conformation. For instance, when human FAK binds with *N*-Methyl-*N*-(3-((2-phenylamino-5-trifluoromethyl-pyrimidin-4-ylamino)-methyl)-pyridin-2-yl)-methanesulfonamide [PDB ID: 6YXV, (Berger et al., 2021), Supplementary Figure S4], the side chain of D toward into the active pocket, while the side chain of F orients outside of the pocket (defined as DFG-in conformation) (Modi and Dunbrack, 2019). Conversely, in the DFG-out conformation, the side chain of D orients out of the ATP-binding site, and that of F packs into the ATP-binding pocket (Roskoski, 2016). Based on the binding models of inhibitors and protein kinases, small molecule protein kinase inhibitors can be classified into six types (Supplementary Figure S5): type I (ATP-binding pocket, DFG-in), type II (ATP-binding pocket, DFG-out), type III (allosteric inhibitor binding next to ATP-binding pocket), type IV (allosteric inhibitor binding far away ATP-binding pocket), type V (bivalent inhibitor), and type VI (covalent inhibitor) (Roskoski, 2016; Modi and Dunbrack, 2019; Arter et al., 2022). The criterion for type-I inhibitors is that the protein kinases should have the DFG-in conformation. Previous studies have shown that when curcumin binds with DYRK2, it acts as a type I inhibitor. Thus, in this work, we assumed that curcumin behaves as type I inhibitor with SIK3 and exhibits the DFG-in and αC-in conformations.

However, some type-I inhibitors, such as dasatinib (Shi et al., 2021a), bosutinib (Shi et al., 2022b), MRT67307 (Shi et al., 2021b), MRT199665 (Shi et al., 2021b), KIN112 (Shi et al., 2021b), and HG-9-91-01 (Shi et al., 2021b), exhibit diverse T-loop conformations while binding with SIK2. Such diverse conformations of the of T-loop have also been reported for type-I inhibitors binding with human FAK (Shi et al., 2022a). Therefore, we speculated that curcumin may also bind with human SIK3 with diverse T-loop conformations, namely the open T-loop (labeled as SIK3-O that T-loop orients far away from the active site) and closed T-loop (defined as SIK3-C that T-loop orients toward the active site) conformations. Atomic information of the open and closed conformations is very important for exploring the binding models between curcumin and SIK3.

In this study, we constructed SIK3-C with closed T-loop and SIK3-O with open T-loop for SIK3 using HM, which were based on the crystal structures of MARK family. The published crystal structures for MARKs were obtained to verify the conformations, and four structures were observed in the overall T-loop residues. The 6C9D (PDB ID) (Emptage et al., 2018) 3FE3 (Nugoor et al., 2008) crystal structures were observed for MARK1 and MARK3, respectively. Meanwhile, the 3IEC and 2WZJ structures were published for MARK2. In addition, the T-loops in 3IEC exhibited the open conformation and those in 2WZJ exhibited the closed conformation. Thus, we selected two crystal structures as templates to construct the closed (known as SIK3-C, PDB ID: 2WZJ (Marx et al., 2010), Figure 2) and open conformation structures [labeled as SIK3-O, PDB ID: 3IEC (Nesic et al., 2010)]. The term root mean square deviation (RMSD) of the backbone was 0.05 Å between SIK3-C and 2WZJ and 0.08 Å between SIK3-O and 3IEC. The overall quality of the modeled structures was also evaluated from their non-bonded atomic interactions using the ERRAT analysis (86.49% and 89.61% for SIK3-O and SIK3-C, respectively, Supplementary Figure S6). The occupancy of residues with average 3D-1D score >0.2 from VERIFY-3D were 82.87% of SIK3-O and 72.27% of SIK3-C (Supplementary Figure S7). Additionally, one or more residues were located in the disallowed regions from the Ramachandran plot analysis. Meanwhile, 91.1% residues for SIK3-O and 89.3% residues for SIK3-C were found in the most favored regions (Supplementary Figure S8). These results indicate that the constructed human SIK3-C and SIK3-O structures were suitable and reliable for molecular docking. At the same time, the difference conformation of T-loop was the mainly diversity between SIK3-O and SIK3-C (Supplementary Figure S9) and the RMSD between SIK3-C and SIK3-O was 1.15 Å. Therefore, the constructed three-dimensional structures for human SIK3 (SIK3-C and SIK3-O) were well to be employed as receptor structures in the subsequent simulations.

**FIGURE 2:**
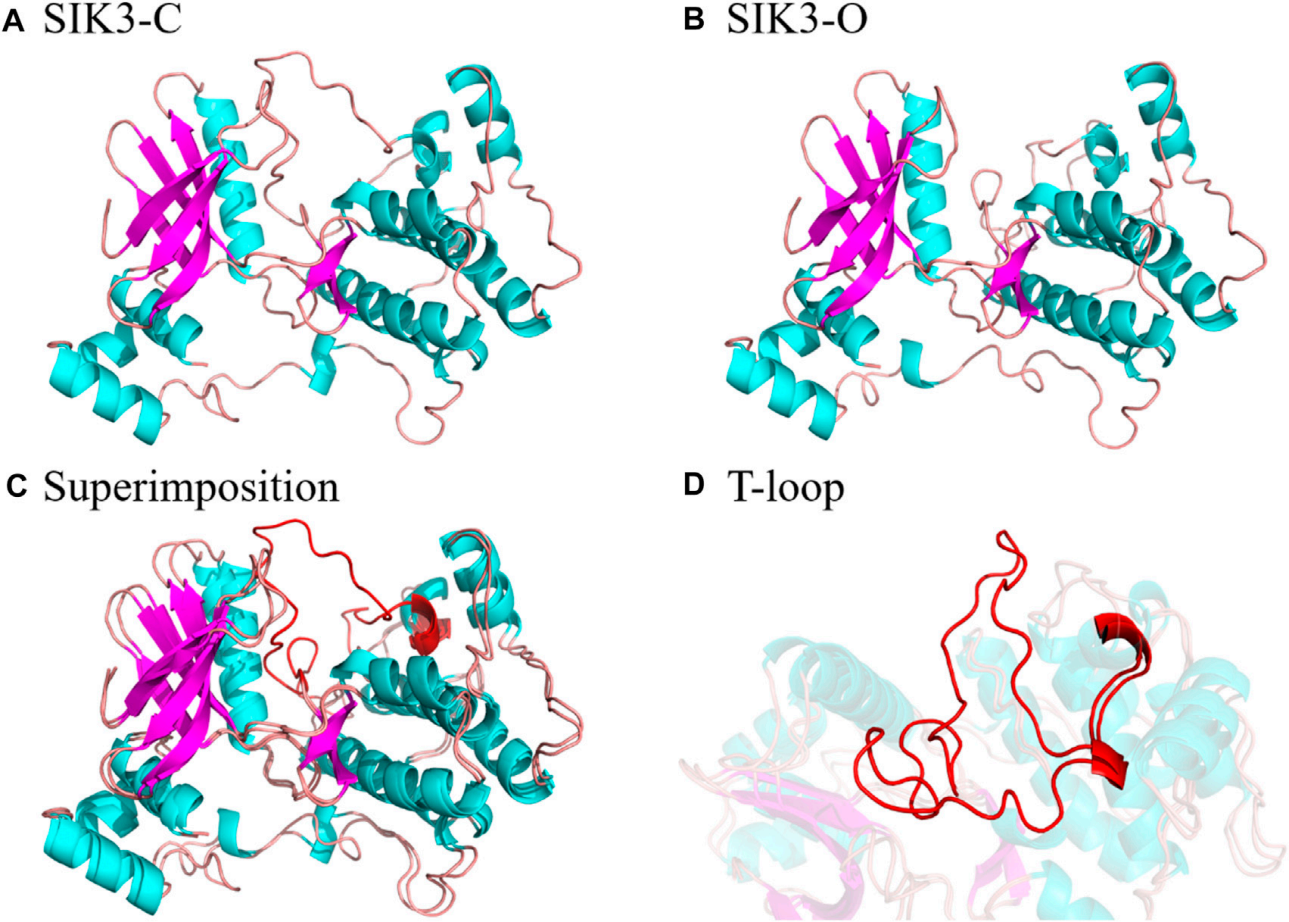
Structures of SIK3 with open and closed conformations of the T-loop. **(A)** Constructed structure of SIK3-C with a closed conformation of the T-loop generated by HM based on MARK2 (PDB ID: 2WZJ). **(B)** Constructed structure of SIK3-O with an open conformation of the T-loop generated by HM based on MARK2 (PDB ID: 3IEC). **(C)** Superimposition of SIK3-O to SIK3-C and **(D)** the enlarged T-loop conformation during the superimposition.

### 3.2 Curcumin structure

Similar to other β-diketo compounds, the diketo (KK) and ketoenol (KE) tautomers of curcumin exist in equilibrium. Both these forms have different potencies to bind with biomolecules (Rege et al., 2019; Osifova et al., 2022). Three polymorph crystal structures have been reported for curcumin (such as the monoclinic and orthorhombic forms) (Supplementary Figure S10) (Tonnesen et al., 1982; Parimita et al., 2007; Sanphui et al., 2011; Matlinska et al., 2018). The KE tautomer can be seen in those three polymorphs curcumin. Notably, the tautomeric forms of curcumin and curcumin derivatives are closely related with the biological activity. For example, the KK form of curcumin exerts antioxidant activity (Jovanovic et al., 1999), while the KE form curcumin is prone to degradation (Rege et al., 2019). In addition, the KE form can penetrate the blood–brain barrier and bind amyloid-beta aggregates. However, the curcumin tautomer that binds with SIK3 has not been identified. Thus, the KK and KE forms of curcumin were considered in this work.

**FIGURE 3:**
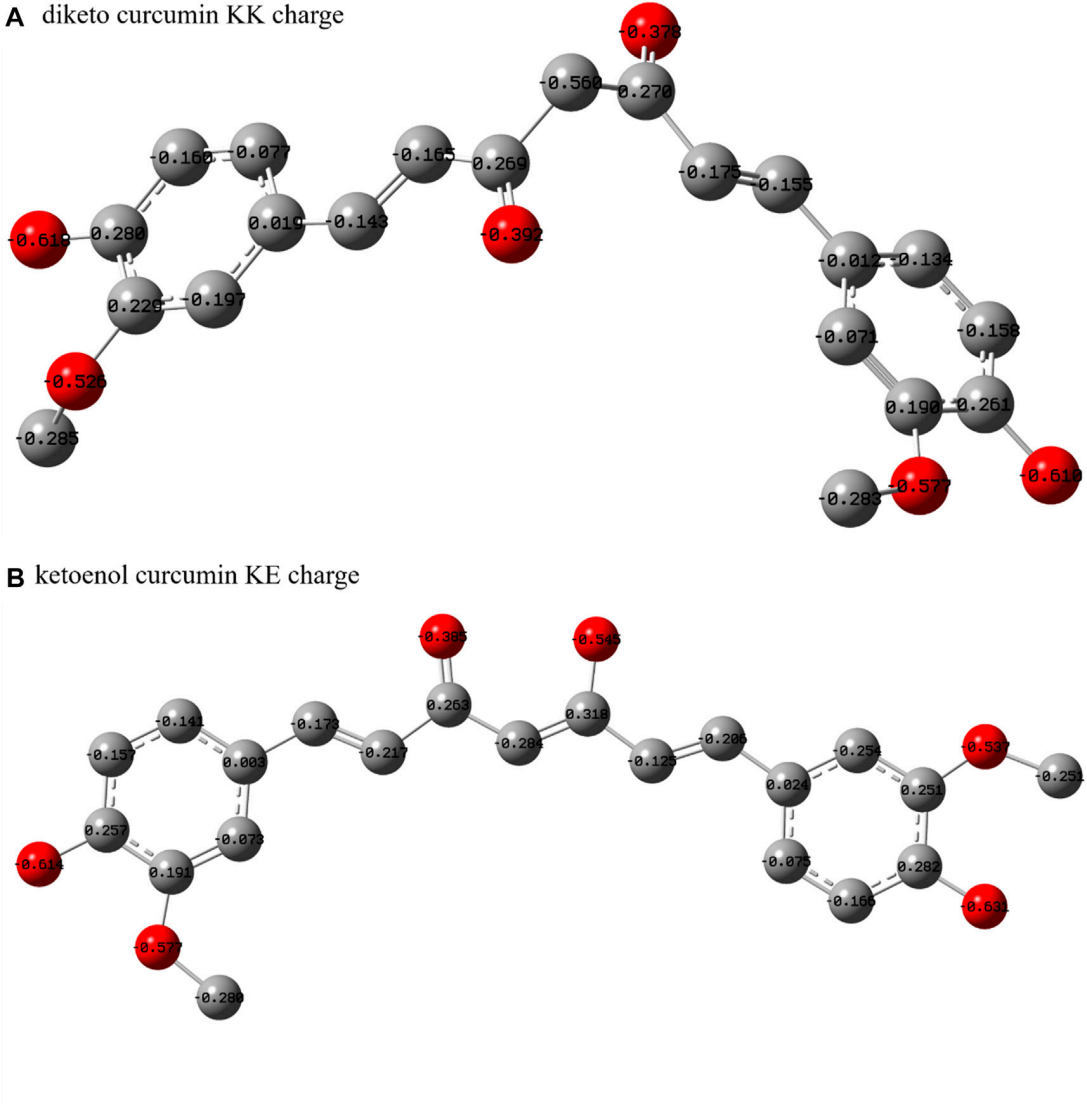
Heavy atom charges of curcumin. **(A)** For diketo curcumin and **(B)** for ketoenol curcumin.

When ligand molecule interacts with receptor protein, the geometry conformation of the ligand molecule must be complementary with the active site of receptor protein. The conformation of curcumin needs to be considered for it binding with human SIK3. Therefore, the density functional theory had been performed to regard the geometries of the KE and KK forms of curcumins. After optimized of the KK and KE forms of curcumin in the gaseous phase, the geometrical parameters are shown in Supplementary Figures S11, S12, respectively. In accordance with the optimized structures, the KE and KK forms adopt planar and non-planer conformations, respectively. Meanwhile, the charge distribution of the two tautomers is also different, especially between the diketo and ketoenol regions (Figure 3 and Supplementary Figure S13). The different protonation states between the KK and KE forms contribute to the differences in their binding models and antitumor activities. Therefore, these two forms of curcumin were docked into the ATP-pocket of human SIK3.

### 3.3 Docking pose analysis

**FIGURE 4:**
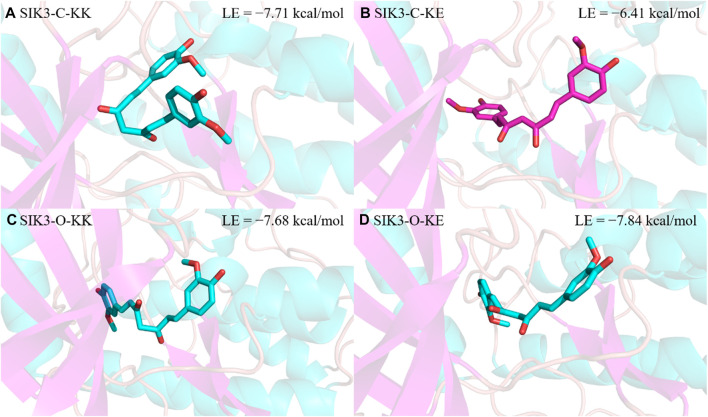
Selected results of curcumin docking with human SIK3. The lowest binding energy was obtained from the docking score. **(A)** For SIK3-C-KK, **(B)** for SIK3-C-KE, **(C)** for SIK3-O-KK, and **(D)** for SIK3-O-KE.

From the binding models of protein kinase inhibitors, we can assume that the binding model for curcumin with SIK3 is based on one key interaction, namely the hydrogen bonding between the hinge loop of SIK3 and the diketo or ketoenol moieties of curcumin. Two thousand conformations of the KK form of curcumin bound to SIK3-C were obtained from the docking experiments; these conformations were initially clustered into 33 clusters with a maximum RMS tolerance of 2.00 Å (Supplementary Figure S14). The occupancies of the top five clusters were >66.9%. Based on the speculated binding model and docking score, Cluster 1 was selected as the SIK3-C_KK conformation with a docking binding energy of −7.71 kcal/mol. Meanwhile, the occupancy of the top five clusters for the binding of KE with SIK3-C also exceeded 67.3% (Supplementary Figure S15). Based on the possible binding models, Cluster 2 was selected as the SIK3-C_KE conformation with a docking binding energy of −6.41 kcal/mol. In addition, Cluster 1 was selected for the possible binding models of KK or KE with SIK3-O, which had docking binding energies of −7.68 and −7.84 kcal/mol, respectively (Supplementary Figures S16, S17). Meanwhile, the occupancies of the top five clusters of SIK3-O_KK and SIK3-O_KE were 58.9% and 90.7%, respectively. As shown in Figure 2, we plotted four representative molecular docking models with different conformations, which were labeled as SIK3-C_KK, SIK3-C_KE, SIK3-O_KK, and SIK3-O_KE (Figure 4). Interestingly, the possible hydrogen bonding network remained between the diketo or ketoenol moieties of curcumin and the A145 residue of the hinge loop of the open or closed T-loop conformations of SIK3, which could potentially facilitate binding (Supplementary Figure S18). Additionally, unstable hydrogen bonds were found in all four complex systems; for instance, the I72 residue formed a hydrogen bond with the hydroxyphenyl group of the KK form of curcumin. However, this hydrogen bond was not observed for the KE form. Furthermore, some residues, such as I72, M117, L195, A205, and L212, formed van der Waals (vdW) interactions with the phenyl ring of curcumin. However, unfavorable donor-donor interactions were observed for SIK3-C_KE, SIK3-O_KK, and SIK3-O_KE systems. This indicates that the molecular dynamics simulations of the complex systems need to be optimized from the docking experiments.

### 3.4 System stability

**FIGURE 5:**
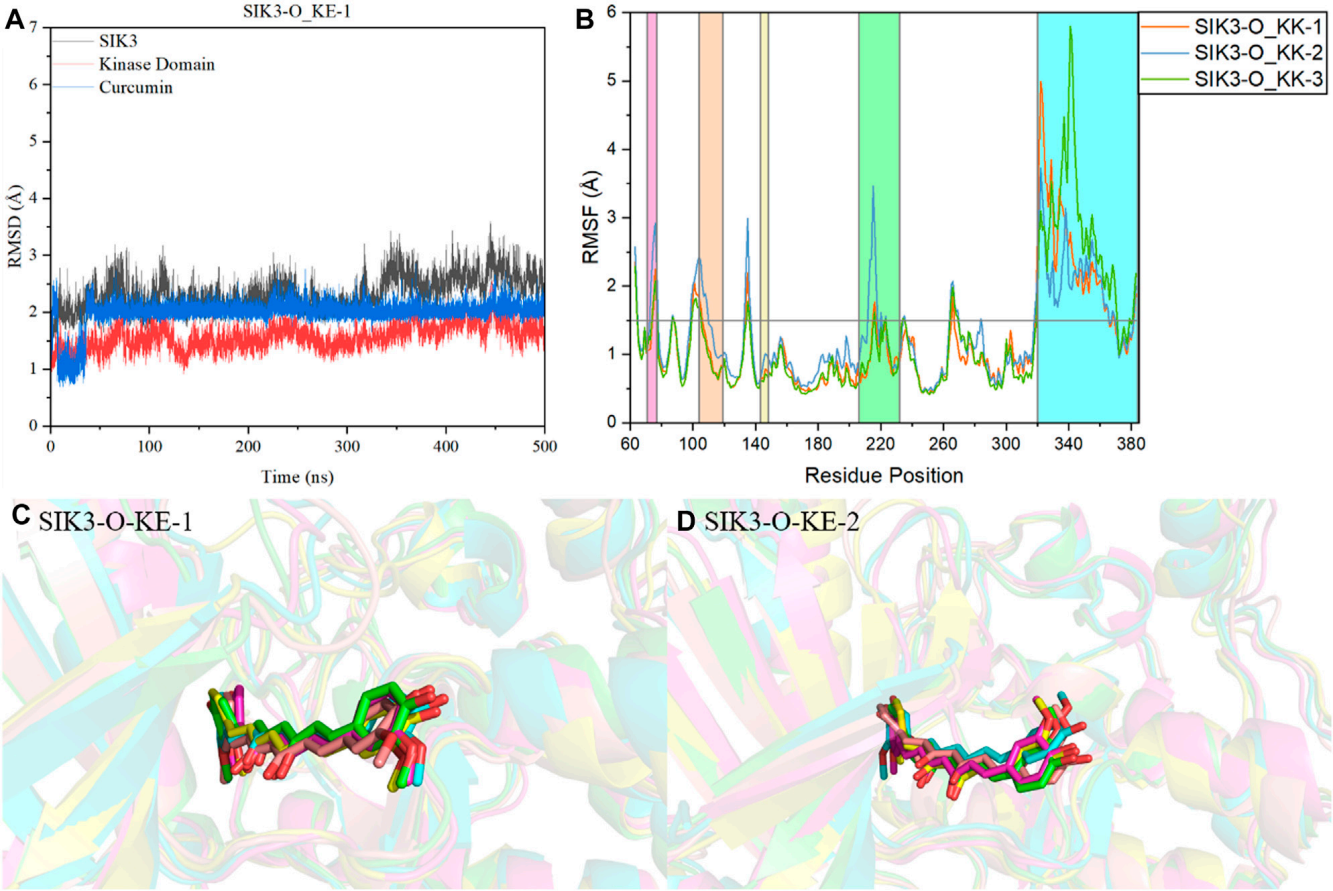
Analysis of molecular dynamics simulations. **(A)** Plot of RMSD of the Cα atom vs. time for the 500-ns MD simulation of SIK3-O-KE-1. **(B)** RMSF variations of the Cα atom of SIK3 from the 500-ns MD simulation of SIK3-O-KE. SIK3-O-KE-1 **(C)** and SIK3-O-KE-2 **(D)** snapshots along the dynamic simulation timeline at 100 ns, 200 ns, 300 ns, 400 ns, and 500 ns. For clarity, the water molecules have been removed. Curcumin is represented by a stick diagram, while SIK3 is depicted by a cartoon diagram.

The RMSD value of the kinase domain (2.36 ± 0.25 Å for residues 66–317) was less than that of the SIK3 (3.17 ± 0.39 Å for residues 63–383) for SIK3-C-KK-2 system (Supplementary Table S1). This result indicates that the kinase domain is more stable than SIK3. This stable kinase domain also can be found in the other curcumin/SIK3 complex systems (Supplementary Figure S19). In addition, the RMSD value of the SIK3-C system was larger than that of the SIK3-O systems. For example, the lowest RMSD value of the kinase domain for the SIK3-C systems was 2.05 ± 0.31 Å, which was larger than the largest RMSD value of the kinase domain for the SIK3-O systems (1.73 ± 0.31 Å) (Figure 5). This phenomenon was also observed for the RMSD values of SIK3. This observation suggests that the SIK3-O complex systems are more stable than the SIK3-C complex systems. However, the RMSD values of both the KK and KE forms of curcumin were higher than those of SIK3 and the kinase domain, especially for the curcumin/SIK3-C systems. Nevertheless, the fluctuations in the RMSD values of curcumin were not related to the KK or KE forms as the RMSD values of curcumin for SIK3-O_KK-1 and SIK3-O_KE-1 were 1.98 ± 0.31 Å and 1.98 ± 0.25 Å, respectively. Simultaneously, we observed that the KE form of curcumin may cause more fluctuations for curcumin than the KK form. For instance, the RMSD values of curcumin for SIK3-C_KK-1 and SIK3-C_KE-1 were 3.64 ± 0.67 and 4.95 ± 0.74 Å, respectively. Thus, the conformation of curcumin fluctuated significantly in the simulation. The closed conformation of the T-loop induced more fluctuations than the open conformation. Supplementary Figure S20 shows the plots of RMSD values vs. time for the SIK3-C-Apo and SIK3-O-Apo systems, which excluded the curcumin molecule (Supplementary Figure S20). The receptor structure was also stable in the simulations after 100 ns, and the kinase domain was more stable than the overall SIK3 structure. The stability of these systems suggests that these simulations can be applied to following analyses.

The root-mean-square fluctuation (RMSF) of the residues of human SIK3 had been analyzed from the 500 ns simulations. Interestingly, there are two regions of human SIK3 with siginifacance fluctuations (Supplementary Figure S21), that is, the regions at positions 206–232 (T-loop) and 344–384 (UBA) (Figure 5). The conformation of the T-loop was different from the crystal structures of protein kinases; for instance, human FAK may exhibit open or closed T-loop conformation while binding with FAK-inhibitors (Supplementary Figure S22) (Guo et al., 2022). The T-loop was phosphorylated to activate the kinase domain and to regulate the downstream signal. In a previous study, protein-protein interactions were observed between SIK2 and 14-3-3 proteins when the T-loop was phosphorylated by LKB1 (Al-Hakim et al., 2005; Sonntag et al., 2018). Meanwhile, bosutinib binds with SIK2 while exhibiting multi-state conformations of the T-loop (Shi et al., 2022b). This flexibility of the T-loop can be observed in the SIK3-C and SIK3-O systems. These observations indicate that the different initial models of T-loops are very important for studying the interactions between inhibitors and SIKs. The UBA region was also found to be flexible; this region directly or indirectly regulates kinase activities (Jaleel et al., 2006; Marx et al., 2006; Wang et al., 2018). The UBA conformations were different for MELK, BRSK2, AMPK, and SNARK, which indicates UBA may form different conformations in SIKs (Wang et al., 2018). Moreover, the UBA region is an important domain for protein kinase autoinhibition (Marx et al., 2006; Cho et al., 2014; Wu et al., 2015; Emptage et al., 2018). In this work, we constructed the UBA region based on MARK2 structures. The RMSF value of the T-loop for the SIK3-O systems was less than that for the SIK3-C systems. In contrast, the RMSF value of the UBA region for the SIK3-O systems was larger than that for the SIK3-C systems. This shows that conformation of UBA and T-loop can be induced from curcumin binding into the ATP-binding site of human SIK3. Furthermore, the overall structure of the kinase domain was stable next to the T-loop region in the simulation.

For clearly demonstrating the conformation fluctuations of curcumin in the ATP-binding pocket of human SIK3, snapshots were obtained from the MD simulation at 100, 200, 300, 400, and 500 ns (Figures 5C, D, and Supplementary Figures S23–S34). However, both KK and KE forms produced binding complexes in the simulation, which indicates that curcumin can directly bind to the ATP-binding pocket of SIK3. In the SIK3-C-KK system, one 4-hydroxy-3-methoxyphenyl group of curcumin was approximately perpendicular to its other 4-hydroxy-3-methoxyphenyl group. This structure was obtained from the docking study. Meanwhile, this class formation was not observed in the other three systems. Curcumin exhibits line structure for the SIK3-C-KE, SIK3-O-KK, and SIK3-O-KE systems. Additionally, one 4-hydroxy-3-methoxyphenyl group of curcumin was exposed to the solvent environment, which may improve its bioavailability (Finlay et al., 2019; Wang et al., 2019). The 4-hydroxy-3-methoxyphenyl group was similar to the 4-methylpiperazine group of bosutinib and the piperazine moiety of the dasatinib (Shi et al., 2021a; Shi et al., 2022b). However, this region of the cyclin-dependent kinase 6 inhibitors can be applied to improve their selectivity among cyclin-dependent kinases (Lu and Schulze-Gahmen, 2006). This surface-exposed portion of the inhibitors can be considered as a modification. Thus, curcumin can form a stable binding model into the active site of the human SIK3 in simulation time.

### 3.5 Hydrogen bond analysis

The hydrogen bonds formed between small chemical scaffold molecules and the hinge loop of protein kinases are commonly required for potential inhibitors. Moreover, hydrogen bonding is an important feature of ATP-competitive inhibitors for protein kinases (Stavenger, 2008; Xing et al., 2015; Pang et al., 2021). Hydrogen bonds maybe significantly contribute to inhibitor binding with human SIK3 as one of protein kinase. Thus, the hydrogen bond numbers formed between curcumin and human SIK3 were first examined for the 12 curcumin/SIK3 complex systems from the simulation trajectory (Supplementary Figures S35). The wide range of hydrogen bond numbers (0–8) for the curcumin/SIK3 systems showed that some unstable hydrogen bonds were formed in the simulations. The SIK3-C-KK system had the lowest hydrogen bond number among the other class systems. The hydrogen bond numbers for SIK3-C-KK-1, SIK3-C-KK-2, and SIK3-C-KK-3 were 2.04 ± 0.80, 1.60 ± 0.70, and 1.76 ± 0.72, respectively (Supplementary Table S2). This indicates that disordered structure of the 4-hydroxy-3-methoxyphenyl group of curcumin forms hydrogen bonds with the back pocket of ATP-binding site of SIK3. Meanwhile, the SIK3-O-KE systems had the largest hydrogen bond numbers among the curcumin/SIK3 complexes (3.72 ± 0.93, 3.19 ± 0.99, and 3.17 ± 1.03 for SIK3-O_KE-1, SIK3-O_KE-2, and SIK3-O_KE-3, respectively). This shows that the KE form of curcumin forms higher number of hydrogen bonds with SIK3 than the KK form (Supplementary Figure S36).

**FIGURE 6:**
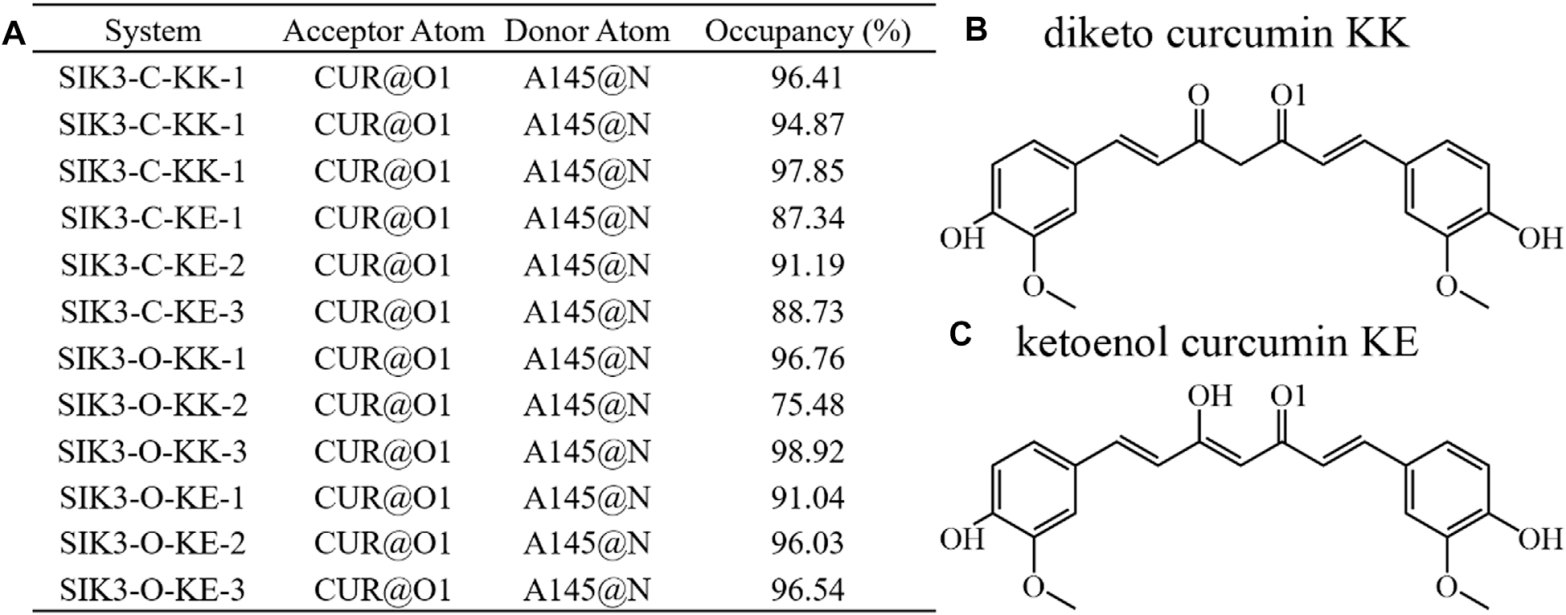
Hydrogen bonding network analysis of interactions between curcumin and human SIK3. **(A)** Occupancy is expressed as % of the period (500 ns, 50,000 frames) during which specific hydrogen bonds are formed. Hydrogen bond is defined as the distance between the acceptor and donor atoms (<3.5 Å), with an internal angle between the H-acceptor and H-donor (>120°). **(B)** and **(C)** labeled the O1 atom of curcumin for diketo and ketoenol curcumin.

The hydrogen bond occupancy analysis of the 12 complex systems was also performed. A hydrogen bond is formed when the distance between the acceptor and donor atoms is shorter than 3.5 Å, and the internal angle of the acceptor···H-donor is larger than 120°. The results of the hydrogen bond occupancy analysis are summarized in Figure 6. High occupancies were found for the diketo and ketoenol groups of curcumin with the hinge loop of SIK3. The occupancies of the KK form were 96.41%, 94.87%, and 97.85% for the SIK3-C-KK-1, SIK3-C-KK-2, and SIK3-C-KK-3 systems, respectively. Meanwhile, the occupancies of the KK form with the hinge loop for SIK3-O (87.34%–91.19%) were less than those of SIK3-C (>94.87%). In contrast, this hydrogen bond forming with A145 of hinge loop was more than 95% for SIK3-O systems with the KK and KE forms of curcumin except for the SIK3-O-KK-2 system (75.48%). However, this occupancy increased to 97.48% when the last 200 ns frames were applied to count the occupancy. Thus, the hydrogen bond between the A145 residue of the hinge loop and the diketo and ketoenol groups of curcumin play an important role in its binding with SIK3. These results agree with those of the experimental and theoretical studies (Shi et al., 2021a; Shi et al., 2021b; Tesch et al., 2021; Shi et al., 2022b). Moreover, the distance between the acceptor and donor atoms was calculated, and the angles between the acceptor atom, hydrogen atom, and donor atom were also checked (Supplementary Figures S37, S38). The distances for SIK3-C-KK-1, SIK3-C-KE-1, SIK3-O-KK-1, and SIK3-O-KE-1were 3.03 ± 0.21, 3.10 ± 0.36, 3.03 ± 0.30, and 3.15 ± 0.23 Å, respectively (Supplementary Table S3). This shows that the hydrogen bond between A145 and the diketo group is as strong as that between A145 and the ketoenol group. These results agreed with occupancy analysis. Especially, the length of the hydrogen bond in the SIK3-O-KK-2 system was greater than 3.5 Å in the first 100 ns, which indicates that no hydrogen bond was formed with the oxygen atom of the diketo group. This can be conformed from the occupancy analysis with 75.48% for 500 ns and 97.48% for the last 200 ns. The hydrogen bond formed between A145 and the hinge loop plays a significant role in the curcumin binding with human SIK3. Additionally, the two-dimensional interaction between curcumin and human SIK2 was drawn based on the representative frame of the maximum cluster for every system using the cluster analysis that had been performed with the average linkage cluster algorithm and epsilon = 2.5 Å from the last 200 ns of the simulation (Supplementary Figure S39). However, the diketo and ketoenol groups are related to the insatiability and low bioavailability of curcumin (Zhi et al., 2022). This limits the potential applications of curcumin. Some studies have overcome the structural disadvantages of curcumin, such as the methyl and methylene groups between 1,3-diketones (Anand et al., 2007; Tu et al., 2017). Thus, other scaffolds that form hydrogen bonds with the hinge loop can be replaced with the diketo and ketoenol groups. For example, adenine, 1H-pyrazole-3-amine, 2-aminopyridine, 1H-pyrrolo [2,3-b]pyridine, 1H-indazole, 7H-pyrrolo [2,3-d]pyrimidin-4-amine, pyrimidine-4,6-diamine, pyridine, 7H-pyrrolo [2,3-d]pyrimidine may be used for this purpose.

### 3.6 Binding free energies

The simulations provide useful information regarding the binding of curcumin with human SIK3. However, the best binding model cannot be identified from these simulations. Thus, four different binding models of curcumin with SIK3 were applied to calculate the binding free energy using the MM/GBSA. The last 200 ns from the simulation trajectory were used herein. The binding free energies of the four complex systems are summarized in Table 1 and Supplementary Figures S4–S15. These calculations were repeated three times for every system. Both KK and KE forms of curcumin can be bound to the ATP-binding pocket of human SIK3 from the binding free energies of the 12 complex systems (−8.20 to −25.86 kcal/mol). The average binding free energies for SIK3-C-KK, SIK3-C-KE, SIK3-O-KK, and SIK3-O-KE were −9.38, −18.47, −18.65, and −22.75 kcal/mol, respectively. SIK3-C-KK showed the biggest binding free energy value, which indicates that the folding of curcumin was unfavorable for its binding with SIK3. In contrast, the line structures of curcumin exhibits better binding affinity for the other three complex systems. The dasatinib also bound with human SIK2 with line structure of dasatinib from our previous study (Shi et al., 2021a). Curcumin was also bound with protein kinase DYRK2 using a line structure (PDB ID: 5ZTN and 6HDR, Supplementary Figure S40) (Banerjee et al., 2018; Elkins and Knapp, 2019). In addition, curcumin was bound with human transthyretin (TTR) using a line structure (PDB ID: 4PME) (Ciccone et al., 2015). However, the non-line conformation of curcumin also binds with TTR (PDB ID: 4PMF) (Ciccone et al., 2015). Thus, the line conformation of curcumin is a better conformation than non-line conformation for binding with SIK3. Furthermore, the binding free energies of SIK3-O (−18.65 and −22.75 kcal/mol) were higher than those for SIK3-C (−9.38 and −18.47 kcal/mol). This suggests that SIK3-O is a more favorable conformation for curcumin binding than SIK3-C. Meanwhile, the KE form of curcumin showed binding affinities of −9.09 and −4.10 kcal/mol for SIK3-C and SIK3-O, respectively. Thus, the binding model for SIK3-O-KE (KE form curcumin binding with open T-loop conformation of human SIK3) is the optimal binding model when curcumin binding with human SIK3.

**TABLE 1:** Binding free energies, decomposition and electrostatic interactions ( $E_{ele}$ ), van der Waals interactions ( $E_{vdW}$ ), solvation free energies ( $E_{polar}$ ), non-polar solvation energies ( $E_{nonpolar}$ ), and entropy ( $TS_{total}$ )^#^ of the curcumin/SIK3 complexes.

Energies	SIK3-C-KK-1	SIK3-C-KK-2	SIK3-C-KK-3	SIK3-C-KE-1	SIK3-C-KE-2	SIK3-C-KE-3	SIK3-O-KK-1	SIK3-O-KK-2	SIK3-O-KK-3	SIK3-O-KE-1	SIK3-O-KE-2	SIK3-O-KE-3
${∆E}_{vdW}$	−40.00	−42.39	−42.01	−36.89	−42.01	−47.88	−45.12	−47.77	−47.39	−45.77	−45.38	−45.24
$∆E_{ele}$	−21.71	−29.66	−23.66	−43.19	−23.39	−26.33	−35.86	−31.93	−21.40	−36.75	−30.33	−29.61
$∆E_{polar}$	37.53	45.12	39.16	48.69	35.22	37.48	47.66	45.97	36.05	42.53	39.77	40.88
${∆E}_{nonpolar}$	−5.87	−6.37	−6.22	−6.14	−6.54	−7.38	−6.94	−7.53	−7.28	−7.07	−6.94	−7.02
${∆E}_{gas}$	−61.71	−72.06	−65.67	−80.08	−65.40	−74.20	−80.98	−79.70	−68.79	−82.52	−75.71	−74.85
${∆E}_{solv}$	31.66	38.75	32.95	42.54	28.68	30.10	40.72	38.44	28.77	35.46	32.83	33.85
${∆(E}_{gas}+E_{sol}$	−30.05	−33.31	−32.73	−37.53	−36.72	−44.10	−40.26	−41.25	−40.02	−47.06	−42.89	−40.99
$∆TS_{total}$	−21.85	−23.47	−22.64	−21.83	−21.89	−19.22	−22.88	−22.27	−20.43	−21.20	−21.42	−20.07
$∆G_{bind}^{cal}$	−8.20	−9.84	−10.09	−15.70	−14.83	−24.88	−17.38	−18.98	−19.59	−25.86	−21.46	−20.92

^#^ ${∆E}_{vdW}$ : Contribution to the binding free energy from van der Waals forces. ${∆E}_{ele}$ : Contribution to the binding free energy from electrostatic interactions. ${∆E}_{polar}$ : Contribution to the binding free energy from polar solvation energies. ${∆E}_{nonpolar}$ : Contribution to the binding free energy from non-polar solvation energies. $∆E_{gas}$ : Contribution to the binding free energy of binding from $∆E_{vdW}$ + ${∆E}_{ele}$ . $∆E_{solv}$ : Contribution to the binding free energy from solvation energies ${∆E}_{polar}$ + ${∆E}_{nonpolar}$ . $∆TS_{total}$ : Contribution to the binding free energy from entropy. $∆G_{bind}^{cal}$ : The final estimated binding free energy from ${∆E}_{gas}+{∆E}_{sol}$ – $∆TS_{total}$ .

^*^The standard errors for all terms are included in the supporting information, which were calculated as the root mean square errors for all the frames extracted from the MM/GBSA, calculation.
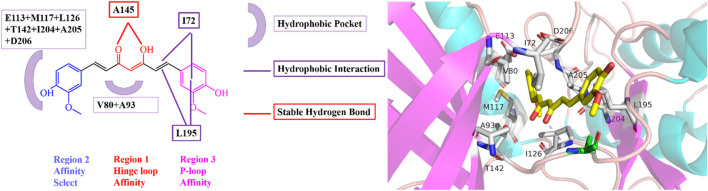

The binding entropies and enthalpies for curcumin and SIK3 are in the range of −19.22 to −23.47 kcal/mol and −30.05–47.06 kcal/mol, respectively. The negative values of both entropy and enthalpy suggest that curcumin binding with human SIK3 is an enthalpy-driven process. The average entropies for curcumin binding with SIK3 are −22.65, −20.98, −21.86, and −20.90 kcal/mol for SIK3-C-KK, SIK3-C-KE, SIK3-O-KK, and SIK3-O-KE, respectively. The entropy values of the four binding systems did not differ significantly, which suggests that entropy does not affect the binding free energy of the systems. However, the differences in the enthalpies of the SIK3-C and SIK3-O conformations were 8.48 and 4.20 kcal/mol for the KK and KE forms of curcumin, respectively. The enthalpy difference between the KK and KE forms of curcumin for SIK3-C and SIK3-O were 7.42 and 3.14 kcal/mol, respectively. Therefore, enthalpy may affect the binding affinity of curcumin toward SIK3.

Meanwhile, the vdW interactions between curcumin and SIK3-C (−41.47 and −42.26 kcal/mol for SIK3-C-KK and SIK3-O-KE, respectively) are less than those between curcumin and SIK3-O (−46.76 and −45.46 kcal/mol for SIK3-C-KK and SIK3-C-KE, respectively). This suggests that the vdW interactions contribute more to the binding of curcumin with SIK3-O than that with SIK3-C. Electronic interactions are also favorable for the formation of the curcumin-SIK3 complex. The contribution of electrostatic interactions to the binding free energies of SIK3-C-KK, SIK3-C-KE, SIK3-O-KK, and SIK3-O-KE was −25.01, −30.97, 29.73, and −32.23 kcal/mol, respectively. Furthermore, the vdW interactions contribute more to the binding free energy than electronic interactions for the four binding models.

Usually, the binding interactions between inhibitors and targets depend on two major factors: the polar term ( $E_{ele}$ + $E_{polar}$ ) and the non-polar term ( $E_{vdW}$ + $E_{nonpolar}$ ). When the KE form of curcumin binds to SIK3-O, the polar terms have positive values. For instance, the polar terms of SIK3-O-KE-1, SIK3-O-KE-2, and SIK3-O-KE-3 are 5.78, 9.44, and 11.27 kcal/mol, respectively. Meanwhile, positive polar terms were obtained for the other three binding models. The polar terms of SIK3-C-KK, SIK3-C-KE, and SIK3-O-KK were 15.59, 9.49, and 13.50 kcal/mol, respectively. This indicates that the polar interactions occur between curcumin and human SIK3. In contrast, the non-polar terms contributed −47.62 kcal/mol for SIK3-C-KK and −48.95 kcal/mol for SIK3-C-KE. The non-polar values of curcumin binding with SIK3-O were less than −52.47 kcal/mol. The negative values (−47.62 kcal/mol) of the non-polar terms suggest that the curcumin binding to SIK3 mainly contributes to the non-polar term. In other words, the polar term was unfavorable for the curcumin binding with human SIK3, and the non-polar term was favorable for this process.

In our previous study (Shi et al., 2022b), the binding free energies for bosutinib bound with ten different conformations of human SIK2 were between −12.81 and −24.72 kcal/mol. Meanwhile, the binding free energies for dasatinib with four different conformations of SIK2 were in the range of −20.09 to −29.37 kcal/mol (Shi et al., 2021a). In addition, the binding free energies for HG-9-91-01, KIN112, MRT67307, and MRT199665 were in the range of −18.81 to −28.40 kcal/mol for three different conformations (Shi et al., 2021b). In this work, the binding free energies of curcumin and human SIK3 were in the −18.47 to −22.75 kcal/mol range, excluding the unfavorable SIK3-C-KK system with the non-liner curcumin structure. Curcumin may bind with SIK3 with similarity binding affinities of other inhibitor binding with SIK2. Therefore, curcumin can be used as a lead compound to develop novel SIKs inhibitors.

### 3.7 Free energy decomposition

The binding of curcumin with human SIK3 can be explained using the four different binding models. However, the key residues that contribute to the complex formation are still unclear. Therefore, the per-residue free energy decomposition method was employed to find the key residues involved in the curcumin-SIK3 complex. The interaction energies for the 12 systems were estimated from the MM/GBSA method with per-residue decomposition (Supplementary Tables S16–S27).

**FIGURE 7:**
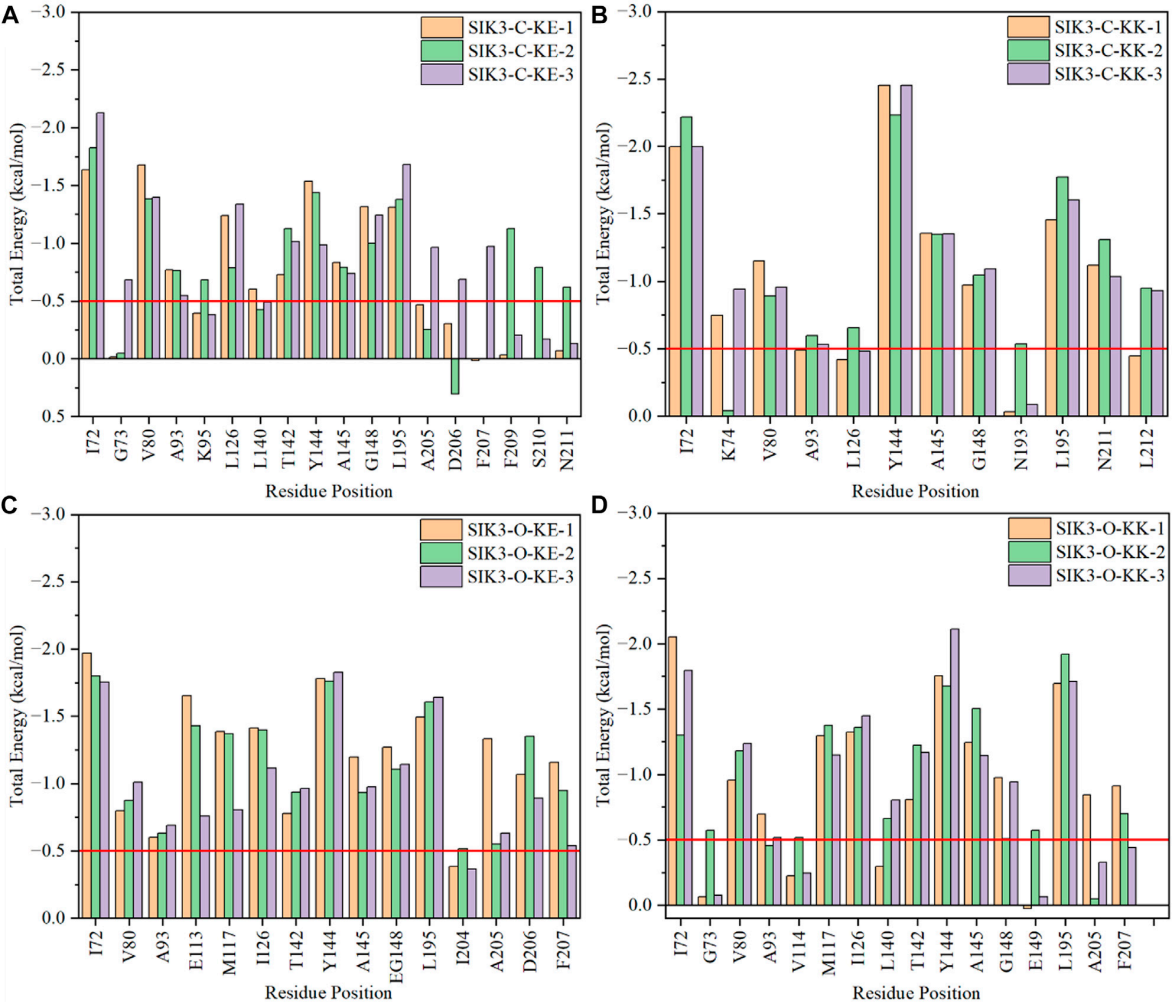
Per-residue binding free energies of selected amino acids in the interactions between curcumin and human SIK3. **(A)** For SIK3-C-KE, **(B)** for SIK3-C-KK, **(C)** for SIK3-O-KE, and **(D)** for SIK3-O-KK.

In the SIK3-C-KK system, the I72, K74, V80, A93, L126, Y144, A145, G148, N193, L195, N211, and L212 residues contributed more than 0.5 kcal/mol (>0.5 kcal/mol in one of the three replicas) (Figure 7). The Y144, A145, and G148 residues are located on the hinge loop, which links the N- and C-lobes to form the ATP-binding pocket (Supplementary Figure S41). A145 forms hydrogen bonds with curcumin and contribute more than −1.35 kcal/mol. Meanwhile, the vdW interactions of Y144 in the SIK3-C-KK-1, SIK3-C-KK-2, and SIK3-C-KK3 systems were −2.24, −1.84, and −2.13 respectively. These interactions mainly arise from the side chain of Y144. The vdW interactions for the SIK3-C-KK-1, SIK3-C-KK-2, and SIK3-C-KK3 systems were −1.56, −1.39, and −1.55 kcal/mol, respectively. In addition, G148 forms vdW interactions with the phenyl ring of 4-hydroxy-3-methoxy phenyl group of curcumin and contributes more than 0.97 kcal/mol. In contrast, the 4-hydroxy-3-methoxy phenyl group of curcumin also interacts with I72 to form a clip between I72 and G148 for the phenyl ring (Supplementary Figure S40). Nevertheless, I72 was not perpendicular to the phenyl and was close to the diketo group of curcumin. Additionally, the L195 residue interacted with the phenyl ring and the diketo group. Thus, the diketo group can be replaced with aromatic groups to enhance the vdW interactions of I72.

I72, G73, V80, A93, K95, L126, L140, T142, Y144, A145, G148, L195, A205, D206, F207, F209, S210, and N211 are the main residues of curcumin that bind with SIK3 in the SIK3-C-KE systems. The I72, Y144, A145, G148, and L195 residues interact similarly with the SIK3-C-KK systems (Supplementary Figure S42). One of the 4-hydroxy-3-methoxy phenyl groups of curcumin was located in the ATP-binding pocket along with V80, A93, K95, L126, L140, T142, A205, D206, and F209 (Supplementary Figure S43). This same pocket was occupied by the 2-chloro-6-methylphenyl ring of dasatinib and other six-membered rings (Shi et al., 2021a). G73 only interacted with the SIK3-C-KE-3 system and was not found in other replicated simulations, which suggest that G73 is unstable and does not interact with curcumin. Thus, if the aromaticity of the phenyl groups is modified to stabilize the interactions between G73 and curcumin, the binding affinity of curcumin toward SIK3 may improve.

The Y144 and A145 residues in the hinge loop of SIK3 play a similar role (hydrogen bond for A145 and vdW interaction for Y144) in the SIK3-O-KK, SIK3-C-KK, and SIK3-C-KE systems (Supplementary Figure S44). This contribution was more in the SIK3-O-KK system (less than −1.65 and −1.14 kcal/mol for Y144 and A145, respectively) than in the SIK3-C-KE systems (less than −1.54 and −0.83 kcal/mol for Y144 and A145, respectively). Meanwhile, the contribution was lower in the SIK3-O-KK system (average value of three replicas with −1.85 and −1.30 kcal/mol for Y144 and A145, respectively) than in the SIK3-C-KK system (average value of three replicas with −2.38 and −1.35 kcal/mol for Y144 and A145, respectively). Thus, Y144 and A145 are the key residues for curcumin binding with SIK3. I72 and L195 form a well flip for the phenyl group in the SIK3-O-KK-1 system but not in the SIK3-O-KK-2 and SIK3-O-KK-3 systems. Which results that I72 has −2.05, −1.30, and −1.80 kcal/mol for SIK3-O-KK-1, SIK3-O-KK-2, and SIK3-O-KK-3, respectively. Thus, if the flip for phenyl group between I72 and L195, the affinity will be increased for curcumin binding with SIK3.

The SIK3-O-KE system exhibited the lowest binding free energy (−22.75 kcal/mol), which suggests that the KE form of curcumin had the high binding affinity with the T-loop open conformation of human SIK3. The hinge loop also formed similar interactions with the other three models (Supplementary Figure S45). Moreover, the I72 residue played a similar role in SIK3-O-KE (−1.84 kcal/mol), SIK3-O-KK (−1.72 kcal/mol), and SIK3-C-KE (−1.86 kcal/mol); however, its contribution was less in the SIK3-C-KK system (−2.07 kcal/mol). This may have occurred because I72 interacts with both the phenyl and diketo groups in SIK3-C-KK. In addition, the back pocket related to ^204^IADFGFSNLF^213^ was important for the line structure of curcumin binding with human SIK3. The DFG motif can be applied to control the kinase activity of protein kinases; DFG-in can be used as the active conformation and DFG-out as the inactive conformation (Modi and Dunbrack, 2019). However, in this work, the DFG-in conformation of SIK3 was considered for type-I kinase inhibitors (Roskoski, 2016; Arter et al., 2022). The conformation of DFG motif may affect the binding models for curcumin and SIK3. Moreover, the DFG motif of human FAK is an important region, which improves the affinity of the FAK inhibitors by forming a hydrogen bond between the DFG motif and inhibitors (Shi et al., 2022a). This indicates that the DFG motif of SIK3 can also be employed to increase the affinity between curcumin and SIK3 by replacing the 4-hydroxy-3-methoxy phenyl group of curcumin.

**FIGURE 8:**
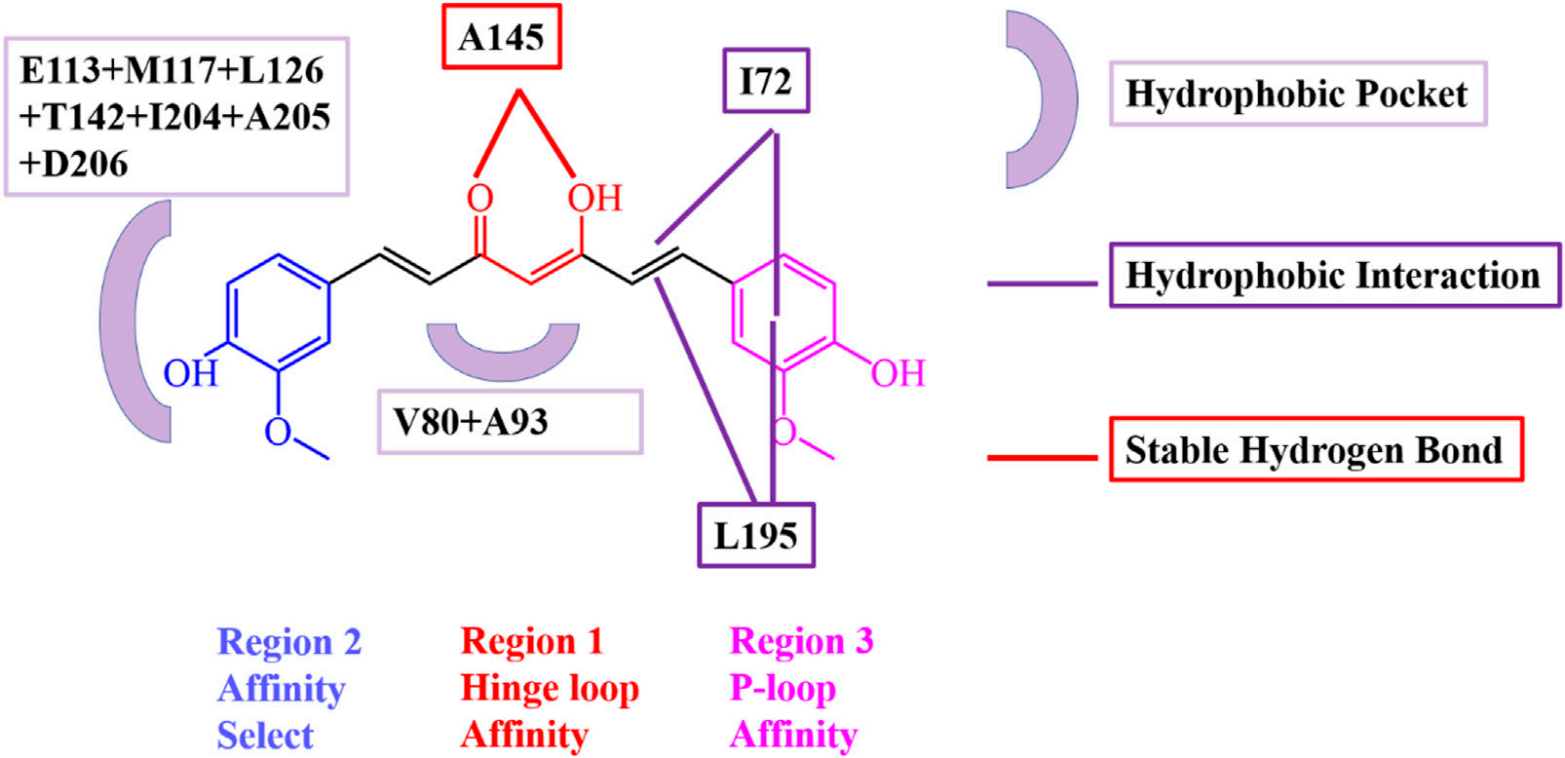
Interaction model of curcumin and human SIK3 obtained from the MD simulations for SIK3-O-KE system. Key residues involved in hydrogen bonding and hydrophobic interactions were highlighted.

Particularly, some hydrophobic residues are shown to have significant subtotal binding free energies for curcumin binding with SIK3. For instance, the I72, V80, A93, Y144, A145, and L195 residues contributed more than −1.30, −0.80, −0.46, −0.98, −0.74, and −1.31 kcal/mol, respectively (Figure 8). Especially, the I72, A145, and L195 residues were the key resides in the four binding models. The A145 residue forms hydrogen bonds with the diketo and ketoenol groups of curcumin. I72 is located in the P-loop, which interacts with the 4-hydroxy-3-methoxy phenyl group. Meanwhile, L195 also forms van der Walls interactions with the 4-hydroxy-3-methoxy phenyl group and the diketo and ketoenol groups of curcumin. Thus, the diketo and ketoenol groups can be replaced with aromatic rings, which remain hydrogen bonds with hinge loop to improve the affinity of curcumin.

### 3.8 Kinase assay

The commercial KinaseProfiler Service (Eurofins Scientific, Inc.) was used to evaluate the IC_50_ of curcumin against human SIK3. KinaseProfiler assay protocols measure the percentage of inhibition of the phosphorylation of a peptide substrate in the presence of fixed concentrations of ATP, which are close to the *K*_m_ values of human SIK3. Curcumin can inhibit the kinase activity of SIK3 with IC50 = 131 nM, with reference to the PKR inhibitor (CAS NO. 608512-97-6). Curcumin remarkably inhibits the kinase activity of human SIK3, which suggests that it can be used as a lead compound for designing and optimizing curcumin derivatives that target human SIK3.

Additionally, the antiproliferative effect of curcumin and HG-9-91-01 was tested for human breast cancer cell lines MDA-MB-231 and MCF-7. The antiproliferative activity of curcumin was significantly higher than that of HG-9-91-01. The IC_50_ values for the antiproliferative activity of curcumin and HG-9-91-01 against MCF-7 were 9.62 ± 0.33 and 15.92 ± 0.90 µM, respectively (Supplementary Figure S46). Meanwhile, the IC_50_ values for curcumin and HG-9-91-01 against MDA-MB-23 were 72.37 ± 0.37 and 55.41 ± 5.11 µM, respectively. These results suggest that curcumin can directly target human SIK3 to inhibit the proliferative activity of MDA-MB-231 and MCF-7.

## 4 Conclusion

In this study, the binding models of curcumin and human SIK3 were studied using computational methods. HM was used to construct the SIK3 kinase domain and UBA region with open/closed conformations of the T-loop (SIK3-C and SIK3-O). Quantum chemistry was employed to calculate the optimized structures and atomic charges of the KK and KE forms of curcumin. Molecular docking was used to obtain the initial complex structures for curcumin and human SIK3 (SIK3-C-KK, SIK3-C-KE, SIK3-O-KK, and SIK3-O-KE). Molecular dynamics simulation was used to obtain the qualitative model for the binding of curcumin with SIK3. Additionally, the binding free energies were calculated for four binding models, and SIK3-O-KE was found to be the optimal model. The three regions of curcumin were identified to increase its binding affinity and to improve its selectivity among protein kinases. Furthermore, in the kinase assay, curcumin showed an IC_50_ value of 131 nM and antiproliferative activities of 9.62 ± 0.33 µM and 72.37 ± 0.37 µM against MCF-7 and MDA-MB-23 cell lines, respectively. This study provides detailed information on the binding of curcumin with human SIK3, which may facilitate the design of novel SIKs inhibitors. The computed and modeled results obtained in this study can be validated by designing curcumin derivatives based on the binding models reported herein. Meanwhile, novel SIKs inhibitors may be developed in the future to treat breast cancer.

## Data Availability

The original contributions presented in the study are included in the article/Supplementary Material, further inquiries can be directed to the corresponding authors.
