# Supplementary material for: Interactions between curcumin and human salt-induced kinase 3 elucidated from computational tools and experimental methods
Source: Front Pharmacol. 2023 Apr 13;14:1116098. doi: 10.3389/fphar.2023.1116098 (PMC10133576; doi:10.3389/fphar.2023.1116098)
Supplement: Supplementary file 1 [file DataSheet1.PDF]

Interactions between curcumin and human salt-induced kinase 3 elucidated from computational tools and experimental methods

Mingsong Shi[1], Yan Zhou[1], Haoche Wei[2], Xinyu Zhang[3], Meng Du[4], Yanting Zhou[5], Yuan Yin[1], Xinghui Li[3], Xinyi Tang[3], Liang Sun[6], Dingguo Xu[4,7,*], Xiaoan Li[1,*]

[1] NHC Key Laboratory of Nuclear Technology Medical Transformation, Mianyang Central Hospital, School of Medicine, University of Electronic Science and Technology of China, Mianyang, Sichuan, 621000, China

[2] State Key Laboratory of Biotherapy/Collaborative Innovation Center of Biotherapy and Cancer Center, West China Hospital, Sichuan University, Chengdu, Sichuan, 610041, China

[3] West China School of Pharmacy, Sichuan University, Chengdu, Sichuan, 610041, China

[4] College of Chemistry, MOE Key Laboratory of Green Chemistry and Technology, Sichuan University, Chengdu, Sichuan, 610065, China

[5] Key Laboratory of Basic Pharmacology of Ministry of Education and Joint International Research Laboratory of Ethnocentric of Ministry of Education, Zunyi Medical University, Zunyi, Guizhou, 563006, China

[6] Shenzhen Shuli Tech Co., Ltd, Shenzhen, Guangdong, 518126, China

[7] Research Center for Material Genome Engineering, Sichuan University, Chengdu, Sichuan, 610065, China

*** Correspondence:**
Corresponding Authors: Dingguo Xu, Xiaoan Li
dgxu@scu.edu.cn, lixiaoan@sc-mch.cn

Content

Binding Free Energy Calculation..S8

Figure S1. Structure for SIKs inhibitor. S11

Figure S2. Methods applied in this work. S12

Figure S3. Structure of kinase domain of human SIK3. ....S13

Figure S4. Crystal structure for human FAK binding with inhibitor. S14

Figure S5. Class of protein kinase inhibitor. S15

Figure S6. ERRAT plot of SIK3 for residue-wise analysis of homology model for SIK3-O and SIK3-C. S16

Figure S7. VERIFY3D analysis results for SIK3-O and SIK3-C models. S17

Figure S8. Ramachandran plot of the models. Validation of the model was done by Ramachandran plot analysis using PROCHECK. S18

Figure S9. Diagram of the SIK3-C and SIK3-O models with T-loop highlight. S19

Figure S10. Crystal structures of curcumin. S20

Figure S11. Optimized geometrical parameters of KK curcumin. S21

Figure S12. Optimized geometrical parameters of KE curcumin. S22

Figure S13. Heavy atom charge of curcumin S23

Figure S14. Docking results for curcumin-KK/SIK3-C system. S24

Figure S15. Docking results for curcumin-KE/SIK3-C system................................S25

Figure S16. Docking results for curcumin-KK/SIK3-O system. S26

Figure S17. Docking results for curcumin-KE/SIK3-O system. S27

Figure S18. Putative binding mode of inhibitor curcumin in the kinase domain of SIK3. ....S28

Figure S19. Root mean square deviation (RMSD) value of heavy atoms of backbone for protein receptor SIK3 and of the ligand curcumin along 500 ns MD simulation for **curcumin/SIK3** systems. S29

Figure S20. Root mean square deviation (RMSD) value of heavy atoms of backbone for protein receptor SIK3 along 500 ns MD simulation for **SIK3-Apo** systems. S30

Figure S21. RMSF variations for Cα atom of SIK3 for Curcumin/SIK3 and Apo-SIK3 systems from the 500 ns MD simulation. S31

Figure S22. Conformation of human FAK binding with inhibitors...........................S32

Figure S23. Snapshots of the SIK3-C-KK-1 along the dynamic simulation time for 100, 200, 300, 400, and 500 ns. S33

Figure S24. Snapshots of the SIK3-C-KK-2 along the dynamic simulation time for 100, 200, 300, 400, and 500 ns. S34

Figure S25. Snapshots of the SIK3-C-KK-3 along the dynamic simulation time for 100, 200, 300, 400, and 500 ns. S35

Figure S26. Snapshots of the SIK3-C-KE-1 along the dynamic simulation time for 100, 200, 300, 400, and 500 ns. S36

Figure S27. Snapshots of the SIK3-C-KE-2 along the dynamic simulation time for 100, 200, 300, 400, and 500 ns. S37

Figure S28. Snapshots of the SIK3-C-KE-3 along the dynamic simulation time for 100, 200, 300, 400, and 500 ns. S38

Figure S29. Snapshots of the SIK3-O-KK-1 along the dynamic simulation time for 100, 200, 300, 400, and 500 ns. S39

Figure S30. Snapshots of the SIK3-O-KK-2 along the dynamic simulation time for 100, 200, 300, 400, and 500 ns. S40

Figure S31. Snapshots of the SIK3-O-KK-3 along the dynamic simulation time for 100, 200, 300, 400, and 500 ns. S41

Figure S32. Snapshots of the SIK3-O-KE-1 along the dynamic simulation time for 100, 200, 300, 400, and 500 ns. S42

Figure S33. Snapshots of the SIK3-O-KE-2 along the dynamic simulation time for 100, 200, 300, 400, and 500 ns. S43

Figure S34. Snapshots of the SIK3-O-KE-3 along the dynamic simulation time for 100, 200, 300, 400, and 500 ns. S44

Figure S35. Hydrogen bond number along the 500-ns MD simulation for curcumin/SIK3. ....S45

Figure S36. Statistical hydrogen bond number profile along the 500-ns MD simulation

for curcumin/SIK3. ....S46

Figure S37. Distance between oxygen atom of diketo or ketoenol group of curcumin and nitrogen atom of A145 in hinge loop for curcumin/SIK3 systems. S47

Figure S38. Angle among oxygen atom of diketo or ketoenol group of curcumin, hydrogen atom of A145, and nitrogen atom of A145 in hinge loop for curcumin/SIK3 systems. S48

Figure S39. Two-dimension interaction between curcumin and human SIK2. ....S49

Figure S40. Crystal structure for curcumin binding with protein. S50

Figure S41. Interaction between curcumin and SIK3 for SIK3-C-KK systems. S51

Figure S42. Interaction between curcumin and SIK3 for SIK3-C-KE systems. S52

Figure S43. Interaction between curcumin and SIK3 for SIK3-C-KE-1 systems for back pocket. S53

Figure S44. Interaction between curcumin and SIK3 for SIK3-C-KK systems. S54

Figure S45. Interaction between curcumin and SIK3 for SIK3-C-KE systems. S55

Figure S46. Cellular activities of curcumin and HG-9-91-01 in human breast cancer cell line MCF-7. ....S56

Table S1. Root mean square deviation (RMSD) value of the SIK3, kinase domain, and curcumin for the curcumin/SIK3 complex systems although 500 ns MD simulations. S57

Table S2. Statistical hydrogen bond number for curcumin/SIK3 systems although 500 ns MD simulations. S58

Table S3. Statistical hydrogen bond distance between A145 and curcumin for curcumin/SIK3 systems although 500 ns MD simulations. S59

Table S4. Binding free energies (ΔG_{bind}^{cal}) for SIK3-C-KK-1 system. S60

Table S5. Binding free energies (ΔG_{bind}^{cal}) for SIK3-C-KK-2 system. S61

Table S6. Binding free energies (ΔG_{bind}^{cal}) for SIK3-C-KK-3 system. S62

Table S7. Binding free energies (ΔG_{bind}^{cal}) for SIK3-C-KE-1 system. S63

Table S8. Binding free energies (ΔG_{bind}^{cal}) for SIK3-C-KE-2 system. S64

Table S9. Binding free energies (ΔG_{bind}^{cal}) for SIK3-C-KE-3 system. S65

Table S10. Binding free energies (**ΔGbindcal**) for SIK3-O-KK-1 system. S66

Table S11. Binding free energies (**ΔGbindcal**) for SIK3-O-KK-2 system...............S67

Table S12. Binding free energies (**ΔGbindcal**) for SIK3-O-KK-3 system. S68

Table S13. Binding free energies (**ΔGbindcal**) for SIK3-O-KE-1 system...............S69

Table S14. Binding free energies (**ΔGbindcal**) for SIK3-O-KE-2 system...............S70

Table S15. Binding free energies (**ΔGbindcal**) for SIK3-O-KE-3 system...............S71

Table S16. Free energy decomposition for the curcumin/SIK3 (**SIK3-C-KK-1**) complex on the individual residue basis, where decomposition is performed in terms of the contributions from van der Waals energy, the electrostatic interaction energy, the nonpolar solvation free energy, the polar solvation free energy, the backbone energy, and the side chain energy S72

Table S17. Free energy decomposition for the curcumin/SIK3 (**SIK3-C-KK-2**) complex on the individual residue basis, where decomposition is performed in terms of the contributions from van der Waals energy, the electrostatic interaction energy, the nonpolar solvation free energy, the polar solvation free energy, the backbone energy, and the side chain energy S73

Table S18. Free energy decomposition for the curcumin/SIK3 (**SIK3-C-KK-3**) complex on the individual residue basis, where decomposition is performed in terms of the contributions from van der Waals energy, the electrostatic interaction energy, the nonpolar solvation free energy, the polar solvation free energy, the backbone energy, and the side chain energy S74

Table S19. Free energy decomposition for the curcumin/SIK3 (**SIK3-C-KE-1**) complex on the individual residue basis, where decomposition is performed in terms of the contributions from van der Waals energy, the electrostatic interaction energy, the nonpolar solvation free energy, the polar solvation free energy, the backbone energy, and the side chain energy S75

Table S20. Free energy decomposition for the curcumin/SIK3 (**SIK3-C-KE-2**) complex on the individual residue basis, where decomposition is performed in terms of the contributions from van der Waals energy, the electrostatic interaction energy, the nonpolar solvation free energy, the polar solvation free energy, the backbone energy, and the side chain energy S76

Table S21. Free energy decomposition for the curcumin/SIK3 (**SIK3-C-KE-3**) complex on the individual residue basis, where decomposition is performed in terms of the contributions from van der Waals energy, the electrostatic interaction energy, the nonpolar solvation free energy, the polar solvation free energy, the backbone energy, and the side chain energy S77

Table S22. Free energy decomposition for the curcumin/SIK3 (**SIK3-O-KK-1**) complex on the individual residue basis, where decomposition is performed in terms of the contributions from van der Waals energy, the electrostatic interaction energy, the nonpolar solvation free energy, the polar solvation free energy, the backbone energy, and the side chain energy S78

Table S23. Free energy decomposition for the curcumin/SIK3 (**SIK3-O-KK-2**) complex on the individual residue basis, where decomposition is performed in terms of the contributions from van der Waals energy, the electrostatic interaction energy, the nonpolar solvation free energy, the polar solvation free energy, the backbone energy, and the side chain energy S79

Table S24. Free energy decomposition for the curcumin/SIK3 (**SIK3-O-KK-3**) complex on the individual residue basis, where decomposition is performed in terms of the contributions from van der Waals energy, the electrostatic interaction energy, the nonpolar solvation free energy, the polar solvation free energy, the backbone energy, and the side chain energy S80

Table S25. Free energy decomposition for the curcumin/SIK3 (**SIK3-O-KE-1**) complex on the individual residue basis, where decomposition is performed in terms of the contributions from van der Waals energy, the electrostatic interaction energy, the nonpolar solvation free energy, the polar solvation free energy, the backbone energy, and the side chain energy S81

Table S26. Free energy decomposition for the curcumin/SIK3 (**SIK3-O-KE-2**) complex on the individual residue basis, where decomposition is performed in terms of the contributions from van der Waals energy, the electrostatic interaction energy, the nonpolar solvation free energy, the polar solvation free energy, the backbone energy, and the side chain energy S82

Table S27. Free energy decomposition for the curcumin/SIK3 (**SIK3-O-KE-3**) complex on the individual residue basis, where decomposition is performed in terms of

the contributions from van der Waals energy, the electrostatic interaction energy, the nonpolar solvation free energy, the polar solvation free energy, the backbone energy, and the side chain energy S83

Binding Free Energy Calculation

In addition to qualitative analysis of ligand/protein binding, quantitative analysis is also important in determining the binding affinity between ligands and proteins. Several methods, such as molecular mechanics/Poisson Boltzmann (or generalized Born) surface area (MM/PBSA or MM/GBSA),(Srinivasan et al., 1998; Lee et al., 2004) solvated interaction energy,(Naim et al., 2007) linear interaction energy,(Aqvist et al., 1994; Bren et al., 2006; Perdih et al., 2009) free energy pathway method,(Gilson and Zhou, 2007)and linear response approximation,(Lee et al., 1992) have been developed to estimate the absolute binding free energy between inhibitors and their target proteins. The MM/GBSA approach is a timesaving and efficient method to evaluate the binding free energy between inhibitors and proteins.(Cheng et al., 2018; King et al., 2021; Shi et al., 2021; Shi et al., 2022; Shirvani and Fassihi, 2022) In this study, only a short description of the calculation of the binding free energy ($\Delta G_{binding}$) from the MM/GBSA method is provided in the following formulae:

$$\Delta G_{binding} = G_{complex} - G_{protein} - G_{ligand} \qquad (1)$$

$$G = E_{gas} + E_{sol} - TS \qquad (2)$$

$$E_{gas} = E_{int} + E_{vdW} + E_{ele} \qquad (3)$$

$$E_{sol} = E_{polar} + E_{nonpolar} \qquad (4)$$

$$G_{nonel} = \gamma \cdot \mathrm{SA} + \mathrm{b} \qquad (5)$$

$G_{complex}$, $G_{protein}$, and G_{ligand} denote the free energies of curcumin/SIK3, SIK3, and curcumin, respectively. G can be decomposed into enthalpy ($H = E_{gas} + E_{sol}$) and entropy (TS). The molecular mechanical energies (E_{gas}) can also be summarized from the intramolecular energy (E_{int}), van der Waals forces (E_{vdW}), and electrostatic forces (E_{ele}). Meanwhile, the contributions of E_{int}, E_{vdW}, and E_{ele} can be obtained through the statistical average based on molecular mechanics. In addition, solvation free energy (E_{sol}) can be divided into polar solvation (E_{polar}) and nonpolar solvation energies ($E_{nonpolar}$). $E_{nonpolar}$ is obtained from the favorable van der Waals interactions between the solute and solvent and the unfavorable cost of surface formation. $E_{nonpolar}$ can be calculated using equation (5), where γ = 0.0072 kcal/Å^2 and b = 0.0 kcal/mol. The linear combination of pairwise overlaps (LCPO) method(Weiser et al., 1999) was employed to estimate the solvent accessible surface

area (SA). However, the E_{polar} contribution was calculated from the GB equation.(Still et al., 1990; Srinivasan et al., 1999) The dielectric constant for the ligand/protein and water were set to 1 and 80, respectively. In addition, normal model analysis was used to calculate the entropy contribution. Thousand snapshots were extracted from the last 200 ns MD trajectory to calculate the statistical average of the MM/GBSA method. The 100 snapshots from the last 200 ns MD trajectory were employed to estimate the entropy contribution.

The binding free energy between curcumin and each residue of human SIK3 was also decomposed for van der Waals (ΔG_{vdW}), electrostatic (ΔG_{ele}), polar solvation (ΔG_{polar}), and nonpolar solvation energies ($\Delta G_{nonpolar}$) using the MM/GBSA method, and the same parameters were applied in the binding free energy calculation. In addition, the free energy decomposition was calculated for the backbone ($B\Delta G_{subtotal}$) and sidechain energies ($S\Delta G_{subtotal}$) for each residue.

References:

Aqvist, J., Medina, C., and Samuelsson, J.E. (1994). A new method for predicting binding affinity in computer-aided drug design. *Protein Eng.* 7(3), 385-391. doi: 10.1093/protein/7.3.385.

Bren, U., Martinek, V., and Florian, J. (2006). Free energy simulations of uncatalyzed DNA replication fidelity: Structure and stability of t center dot g and dttp center dot g terminal DNA mismatches flanked by a single dangling nucleotide. *J. Phys. Chem. B* 110(21), 10557-10566. doi: 10.1021/jp060292b.

Cheng, P., Li, J.J., Wang, J., Zhang, X.Y., and Zhai, H.L. (2018). Investigations of fak inhibitors: A combination of 3d-qsar, docking, and molecular dynamics simulations studies. *J. Biomol. Struct. Dyn.* 36(6), 1529-1549. doi: 10.1080/07391102.2017.1329095.

Gilson, M.K., and Zhou, H.X. (2007). Calculation of protein-ligand binding affinities. *Annu. Rev. Biophys. Biomolec. Struct.* 36, 21-42. doi: 10.1146/annurev.biophys.36.040306.132550.

King, E., Aitchison, E., Li, H., and Luo, R. (2021). Recent developments in free energy calculations for drug discovery. *Front. Mol. Biosci.* 8, 24. doi: 10.3389/fmolb.2021.712085.

Lee, F.S., Chu, Z.T., Bolger, M.B., and Warshel, A. (1992). Calculations of antibody-antigen interactions: Microscopic and semi-microscopic evaluation of the free energies of binding of phosphorylcholine analogs to mcpc603. *Protein Eng.* 5(3), 215-228. doi: 10.1093/protein/5.3.215.

Lee, M.S., Salsbury, F.R., and Olson, M.A. (2004). An efficient hybrid explicit/implicit solvent method for biomolecular simulations. *J. Comput. Chem.* 25(16), 1967-1978. doi: 10.1002/jcc.20119.

Naim, M., Bhat, S., Rankin, K.N., Dennis, S., Chowdhury, S.F., Siddiqi, I., et al. (2007). Solvated interaction energy (sie) for scoring protein-ligand binding affinities. 1. Exploring the parameter space. *J. Chem Inf. Model.* 47(1), 122-133. doi:

10.1021/ci600406v.

Perdih, A., Bren, U., and Solmajer, T. (2009). Binding free energy calculations of n-sulphonyl-glutamic acid inhibitors of murd ligase. *J. Mol. Model.* 15(8), 983-996. doi: 10.1007/s00894-009-0455-8.

Shi, M., Chen, T., Wei, S., Zhao, C., Zhang, X., Li, X., et al. (2022). Molecular docking, molecular dynamics simulations, and free energy calculation insights into the binding mechanism between vs-4718 and focal adhesion kinase. *ACS Omega* 7(36), 32442-32456. doi: 10.1021/acsomega.2c03951.

Shi, M., Wang, L., Li, P., Liu, J., Chen, L., and Xu, D. (2021). Dasatinib-sik2 binding elucidated by homology modeling, molecular docking, and dynamics simulations. *ACS Omega* 6(16), 11025-11038. doi: 10.1021/acsomega.1c00947.

Shirvani, P., and Fassihi, A. (2022). In silico design of novel fak inhibitors using integrated molecular docking, 3d-qsar and molecular dynamics simulation studies. *J. Biomol. Struct. Dyn.* 40(13), 5965-5982. doi: 10.1080/07391102.2021.1875880.

Srinivasan, J., Cheatham, T.E., Cieplak, P., Kollman, P.A., and Case, D.A. (1998). Continuum solvent studies of the stability of DNA, rna, and phosphoramidate - DNA helices. *J. Am. Chem. Soc.* 120(37), 9401-9409. doi: 10.1021/ja981844+.

Srinivasan, J., Trevathan, M.W., Beroza, P., and Case, D.A. (1999). Application of a pairwise generalized born model to proteins and nucleic acids: Inclusion of salt effects. *Theor. Chem. Acc.* 101(6), 426-434. doi: 10.1007/s002140050460.

Still, W.C., Tempczyk, A., Hawley, R.C., and Hendrickson, T. (1990). Semianalytical treatment of solvation for molecular mechanics and dynamics. *J. Am. Chem. Soc.* 112(16), 6127-6129. doi: 10.1021/ja00172a038.

Weiser, J., Shenkin, P.S., and Still, W.C. (1999). Approximate atomic surfaces from linear combinations of pairwise overlaps (lcpo). *J. Comput. Chem.* 20(2), 217-230. doi: 10.1002/(sici)1096-987x(19990130)20:2<217::Aid-jcc4>3.0.Co;2-a.

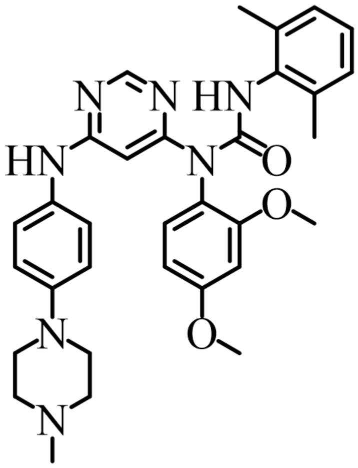

HG-9-91-01

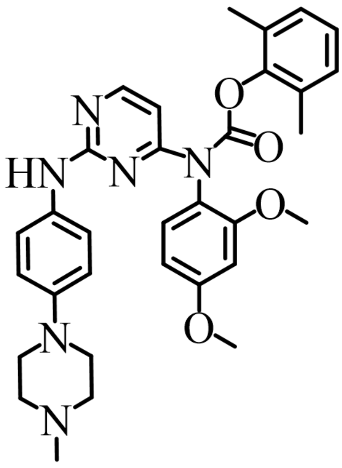

KIN112

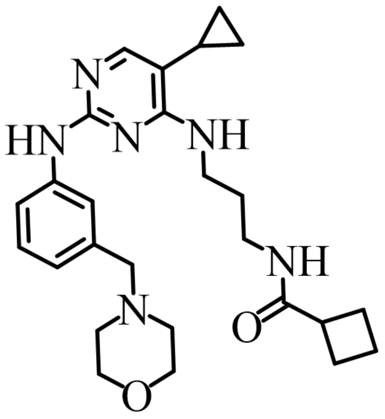

MRT67307

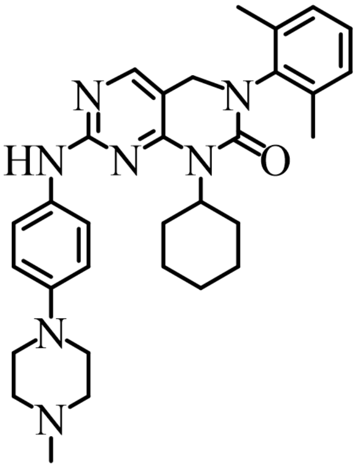

YKL-06-062

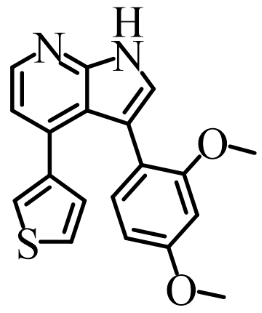

ARN-3236

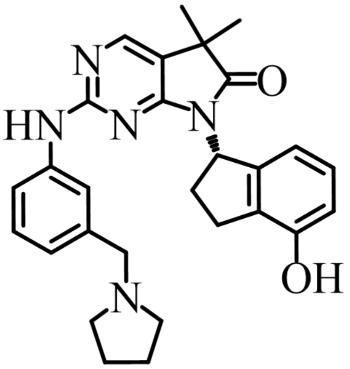

MRT199665

Figure S1. Structure for SIKs inhibitor.

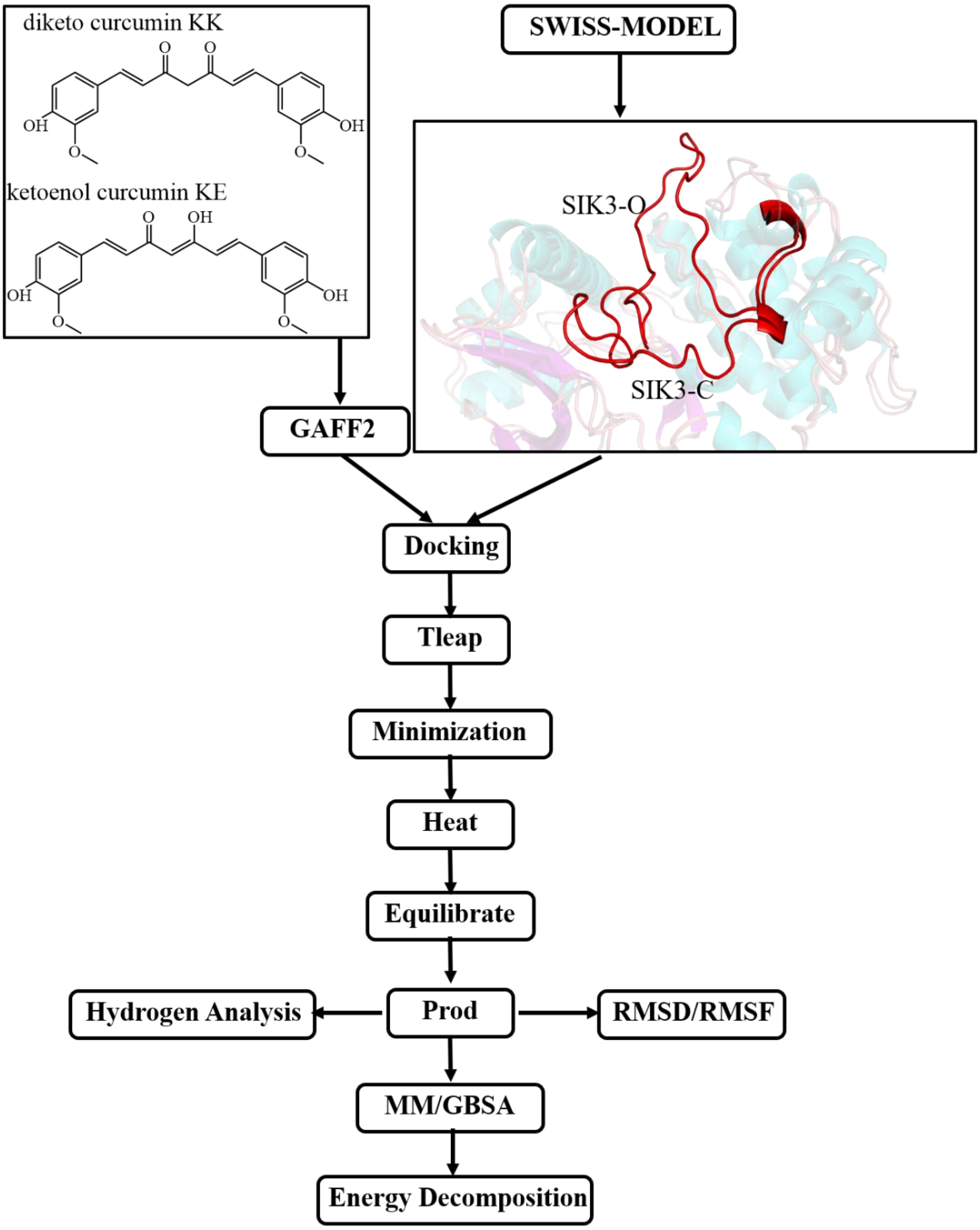

Figure S2. Methods applied in this work.

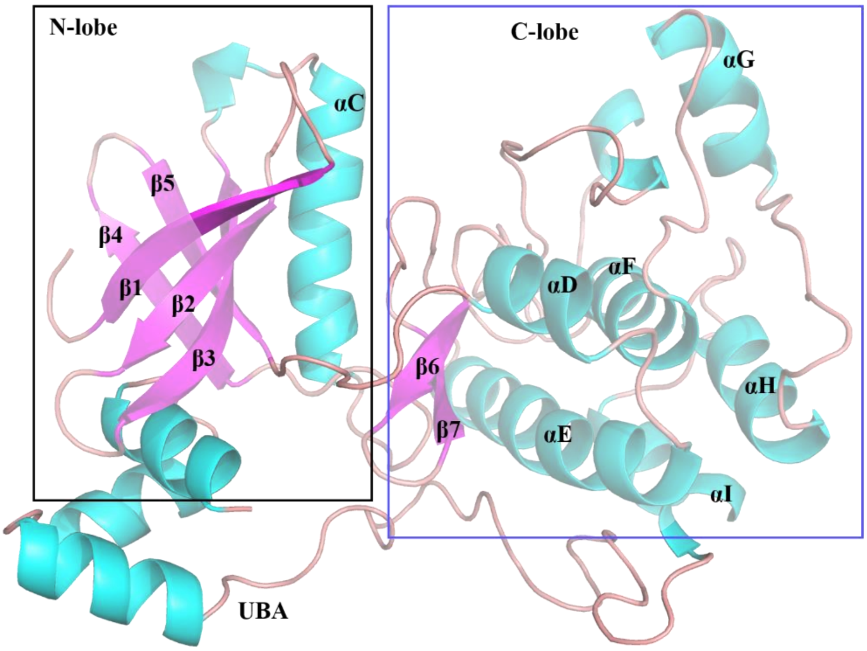

Figure S3. Structure of kinase domain of human SIK3.

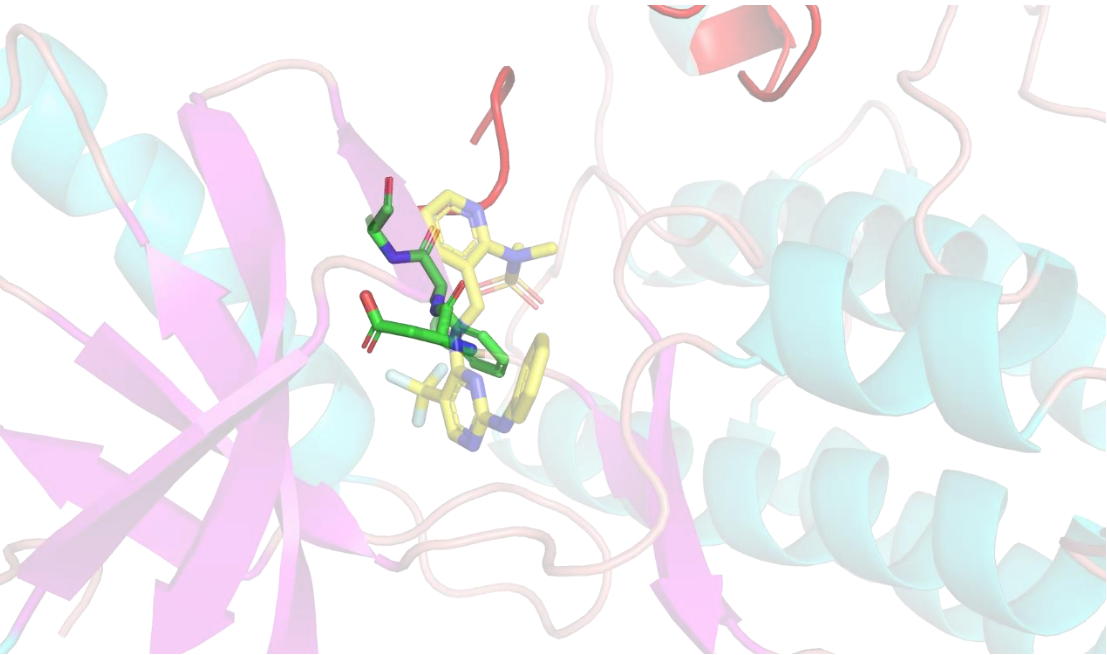

Figure S4. Crystal structure for human FAK binding with inhibitor.
The human FAK was bound with N-Methyl-N-(3-((2-phenylamino-5-trifluoromethyl-pyrimidin-4-ylamino)-methyl)-pyridin-2-yl)-methanesulfonamide (PDB ID: 6YXV).

**Protein Kinase Inhibitors**				
Type I	DFG-in	αC-in	ATP-pocket	Competitive
Type $I_{1/2}$	DFG-in	αC-out	ATP-pocket	Competitive
Type II	DFG-out	αC-in or αC-out	ATP-pocket	Competitive
Type III	DFG-in or DFG-out	αC-in or αC-out	ATP-pocket + next to ATP-pocket	Competitive Allosteric
Type IV	DFG-in or DFG-out	αC-in or αC-out	Far away ATP-pocket	Allosteric
Type V	DFG-in or DFG-out	αC-in or αC-out	Two different regions	
Type VI	DFG-in or DFG-out	αC-in or αC-out	Mainly ATP-pocket	

Figure S5. Class of protein kinase inhibitor.

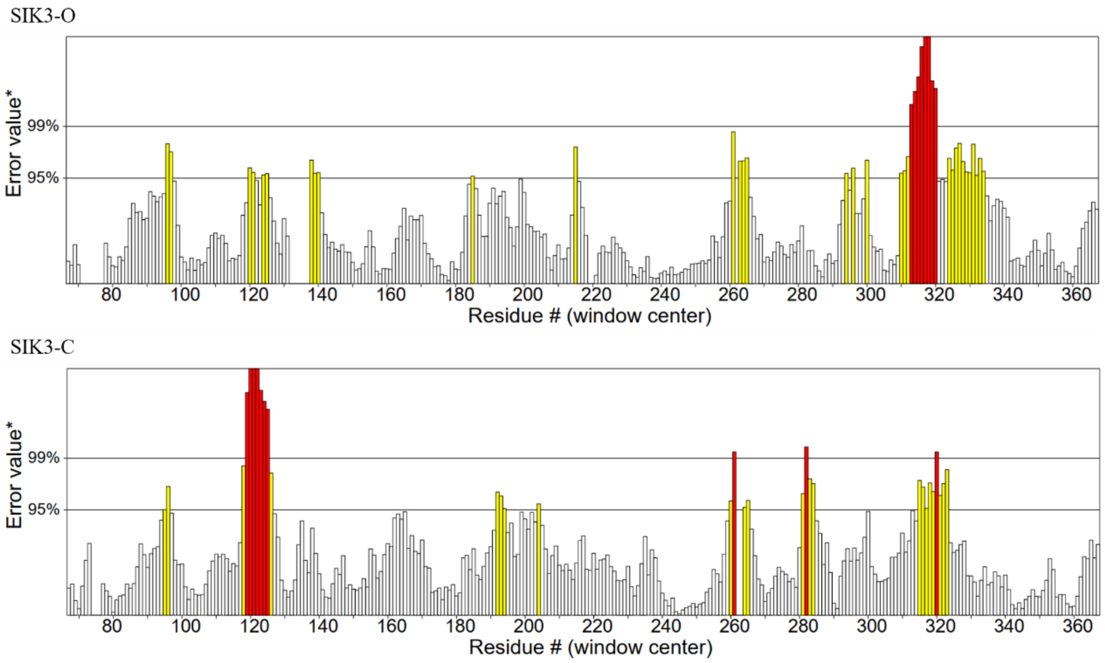

Figure S6. ERRAT plot of SIK3 for residue-wise analysis of homology model for SIK3-O and SIK3-C.

For the SIK3-C model, the 95-96, 118-127, 192-194, 204, 260-261, 263-264, 281-284, and 315-323 residues can be rejected. Meanwhile, the 96-97, 120-121,125-126, 138-140, 185, 215, 261, 263-265, 294, 296, 300, 310-320, and 324-334 residues can also be rejected. The most possible regions for rejection were the loop region between β4 and β5 for SIK3-C and the loop region in the UBA domain for SIK3-O. However, these residues will be optimized in simulation and further away from the ATP-binding site of human SIK3. Thus, this homology modeling can be applied to other studies.

SIK3-O

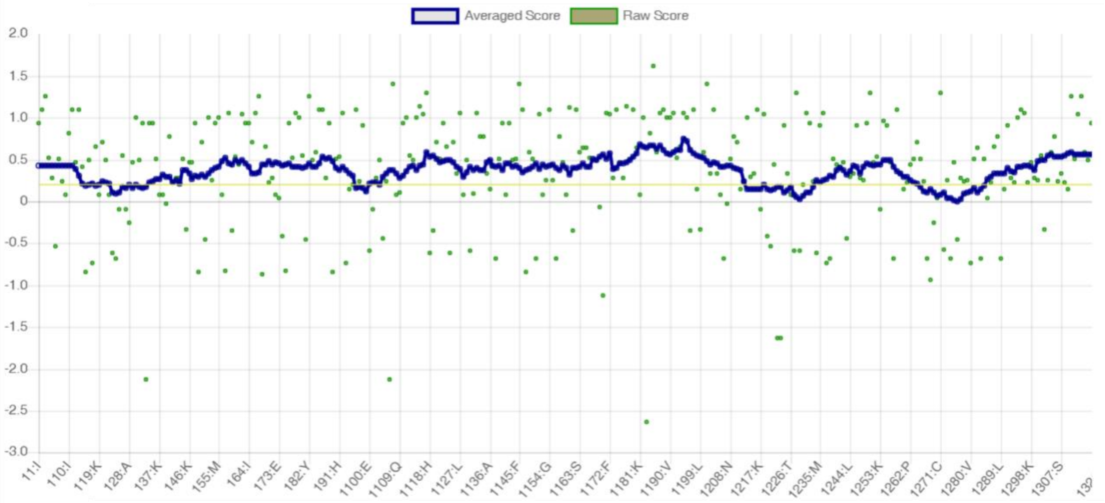

SIK3-C

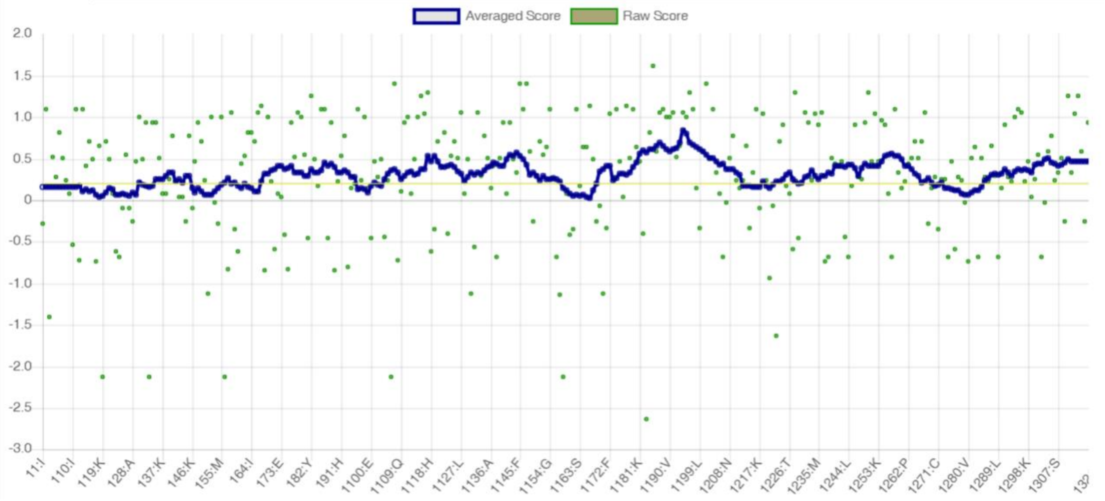

Figure S7. VERIFY3D analysis results for SIK3-O and SIK3-C models.
For SIK3-C, 72.27% of the amino acids have scored >= 0.2 in the 3D/1D profile. For SIK3-O, 82.87% of the amino acids have scored >= 0.2 in the 3D/1D profile.

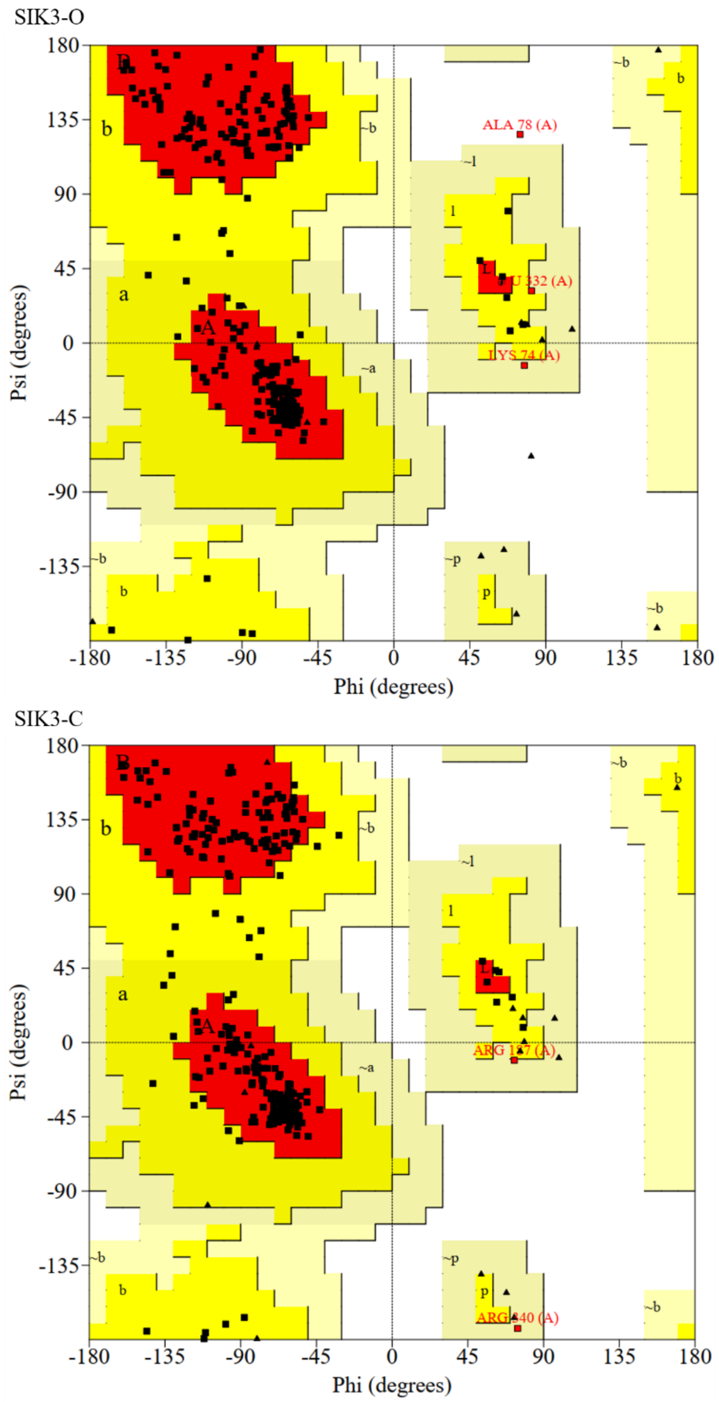

Figure S8. Ramachandran plot of the models. Validation of the model was done by Ramachandran plot analysis using PROCHECK.

Residues in most favoured regions were 89.3% for SIK3-C and 91.1% for SIK3-O ;

Residues in additional allowed regions were 10.0% for SIK3-C and 7.9% for SIK3-O;

Residues in generously allowed regions were 0.7% for SIK3-C and 0.7% for SIK3-O;

Residues in disallowed regions were 0.0% for SIK3-C and 0.3% for SIK3-O.

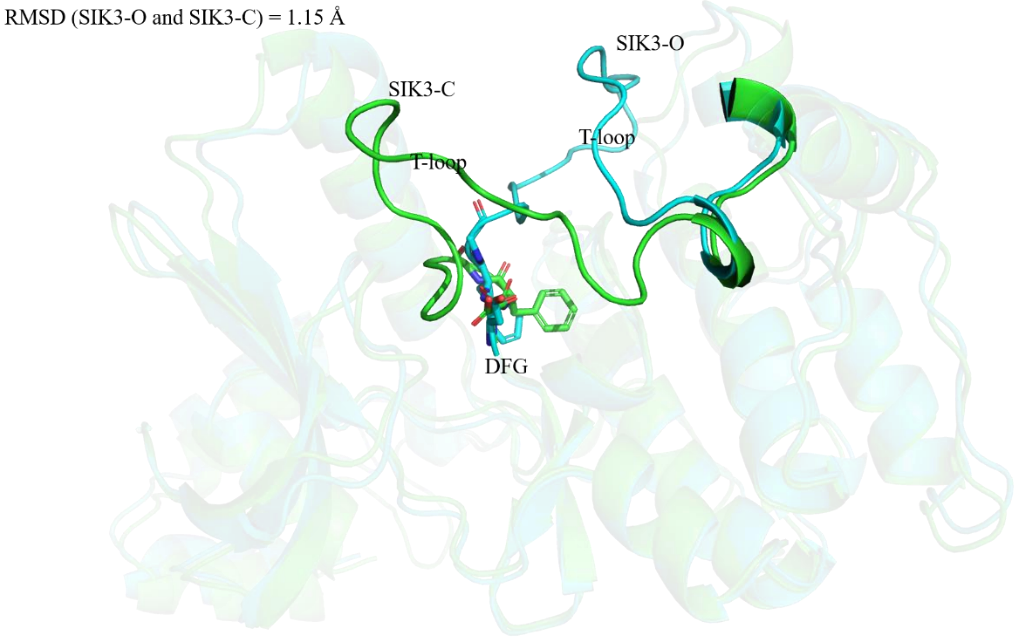

Figure S9. Diagram of the SIK3-C and SIK3-O models with T-loop highlight.

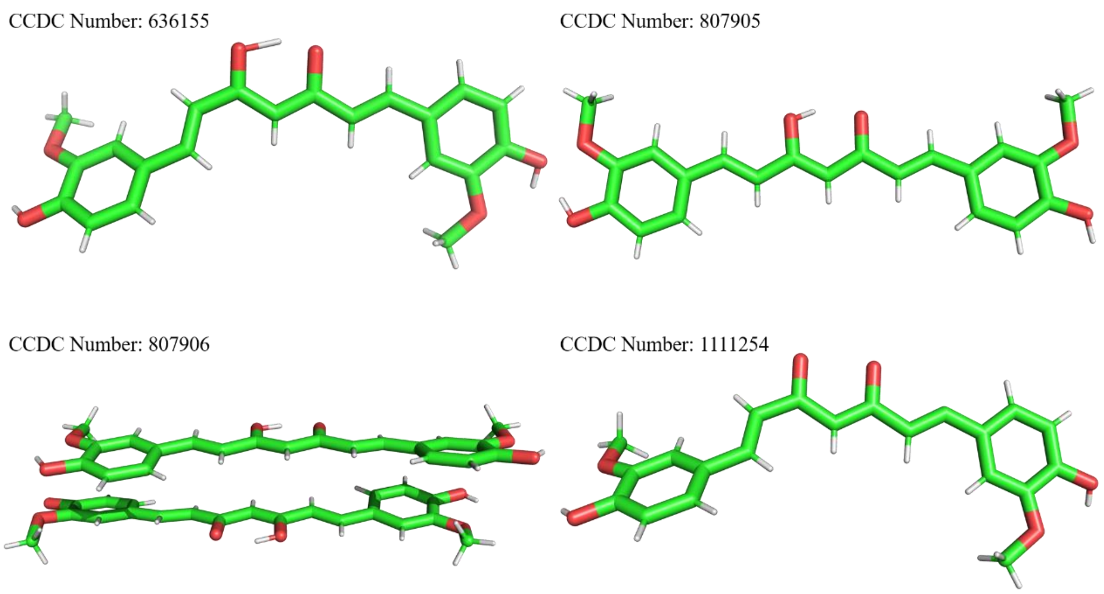

Figure S10. Crystal structures of curcumin.
Those structures were obtained from WebCSD.

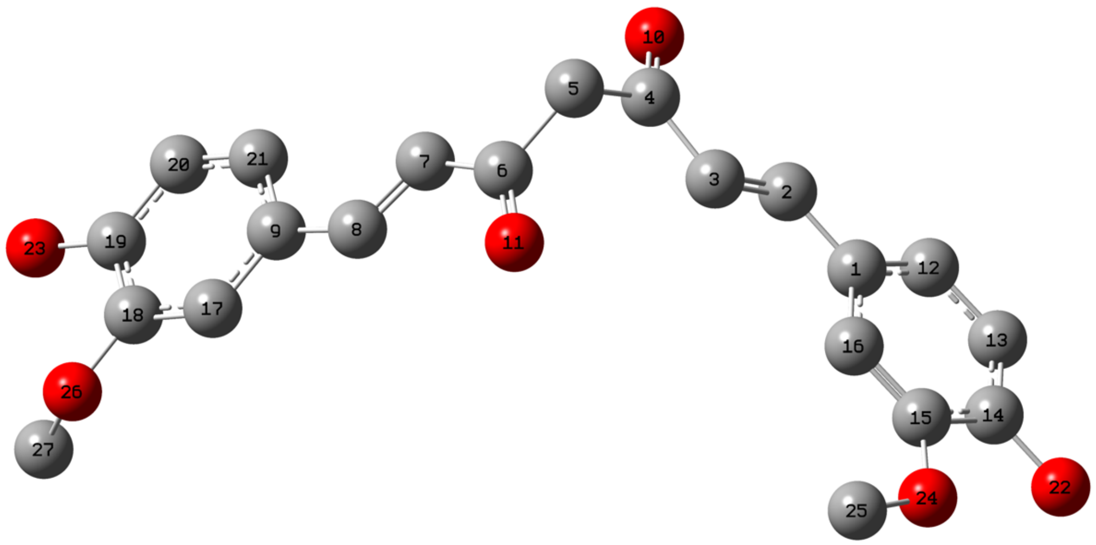

Atom Number	Element	Atom 1	Atom 2	Atom 3	Bond Å	Angle °	Dihedral °
1	C						
2	C	1			1.46		
3	C	2	1		1.35	127.85	
4	C	3	2	1	1.47	121.62	-179.77
5	C	4	3	2	1.53	116.46	-177.02
6	C	5	4	3	1.52	111.14	-65.72
7	C	6	5	4	1.47	116.93	-82.55
8	C	7	6	5	1.35	121.44	-178.55
9	C	8	7	6	1.46	127.96	-179.76
10	O	4	3	2	1.25	123.62	3.36
11	O	6	5	4	1.25	120.00	95.65
12	C	1	2	3	1.41	119.11	-177.90
13	C	12	1	2	1.40	121.36	179.98
14	C	13	12	1	1.39	119.56	0.12
15	C	14	13	12	1.41	119.86	0.03
16	C	15	14	13	1.38	120.79	-0.12
17	C	9	8	7	1.41	118.74	-178.91
18	C	17	9	8	1.39	122.07	179.83
19	C	18	17	9	1.41	118.95	-0.17
20	C	19	18	17	1.40	119.80	1.09
21	C	20	19	18	1.39	120.79	-1.10
22	O	14	13	12	1.38	120.36	179.91
23	O	19	18	17	1.39	117.92	-178.44
24	O	15	14	13	1.40	112.90	179.78
25	C	24	15	14	1.45	119.11	-179.55
26	O	18	17	9	1.39	117.32	-176.33
27	C	26	18	17	1.47	119.07	-130.38

Figure S11. Optimized geometrical parameters of KK curcumin.

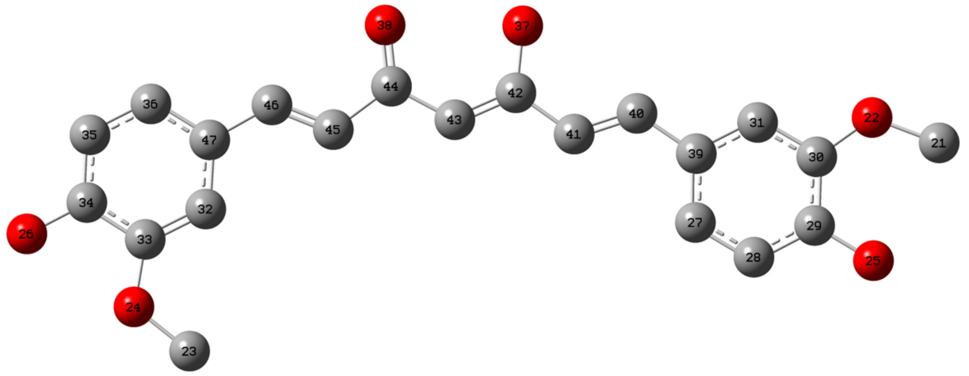

Atom Number	Element	Atom 1	Atom 2	Atom 3	Bond Å	Angle °	Dihedral °
21	C						
22	O	21			1.46		
23	C	22	21		14.73	140.25	
24	O	23	22	21	1.45	122.44	179.82
25	O	21			2.76		
26	O	24	23	22	2.65	175.80	-179.95
27	C	25	21		3.71	106.96	
28	C	27	25	21	1.39	18.67	-179.92
29	C	28	27	25	1.40	121.51	0.01
30	C	22	21		1.39	123.69	
31	C	30	22	21	1.40	114.21	179.86
32	C	24	23	22	2.48	92.40	0.00
33	C	32	24	23	1.38	27.07	-180.00
34	C	26	24	23	1.38	62.11	179.95
35	C	34	26	24	1.39	120.39	-180.00
36	C	35	34	26	1.40	119.62	180.00
37	O	31	30	22	5.18	172.19	179.91
38	O	37	31	30	2.81	157.85	0.17
39	C	31	30	22	1.41	122.72	179.99
40	C	39	31	30	1.46	118.68	-180.00
41	C	40	39	31	1.35	127.38	-179.99
42	C	37	31	30	1.38	73.44	0.12
43	C	42	37	31	1.36	119.74	179.97
44	C	38	37	31	1.26	83.82	-0.04
45	C	44	38	37	1.48	120.89	-179.99
46	C	45	44	38	1.35	121.22	-0.01
47	C	36	35	34	1.41	121.48	0.00

Figure S12. Optimized geometrical parameters of KE curcumin.

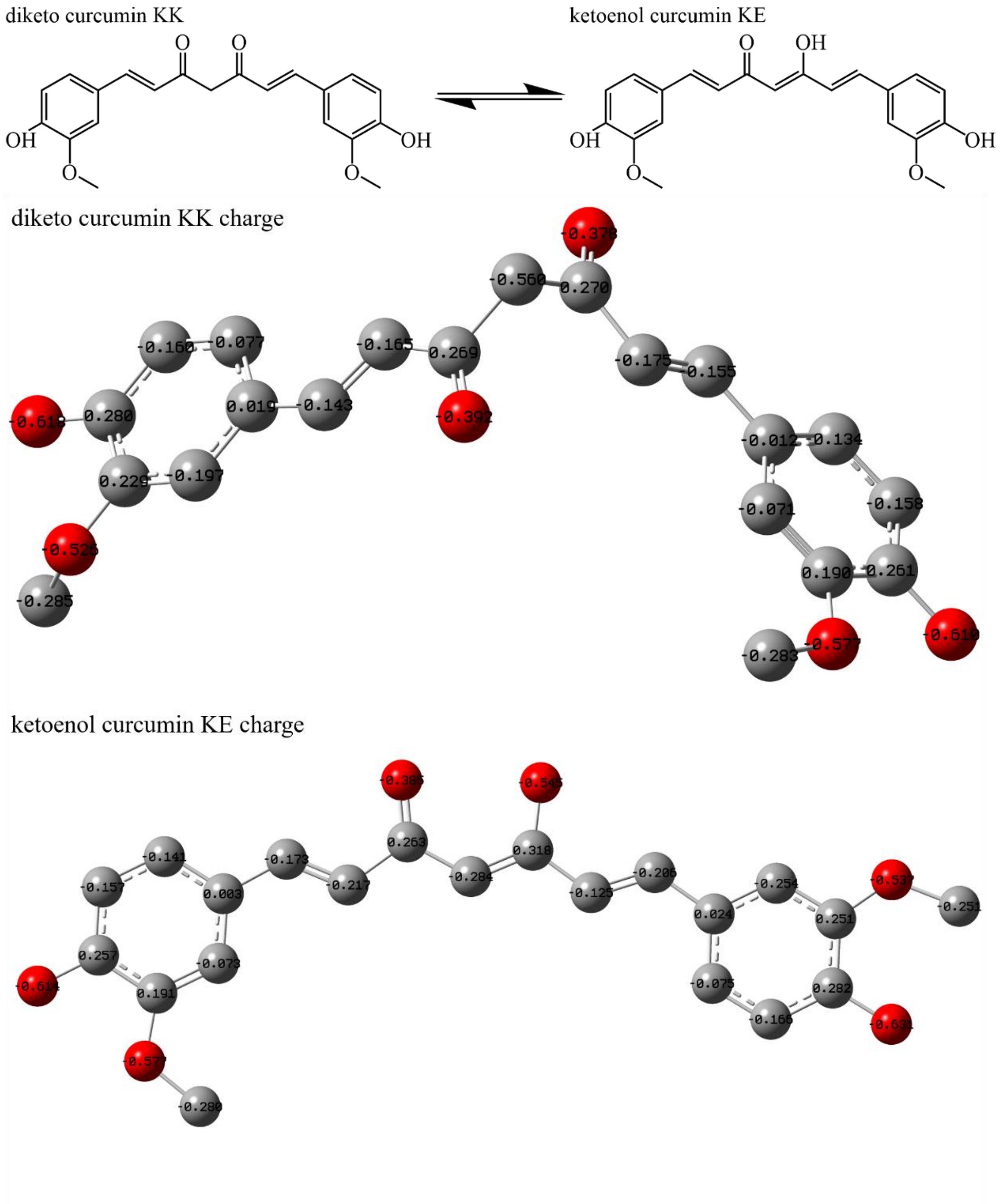

Figure S13. Heavy atom charge of curcumin

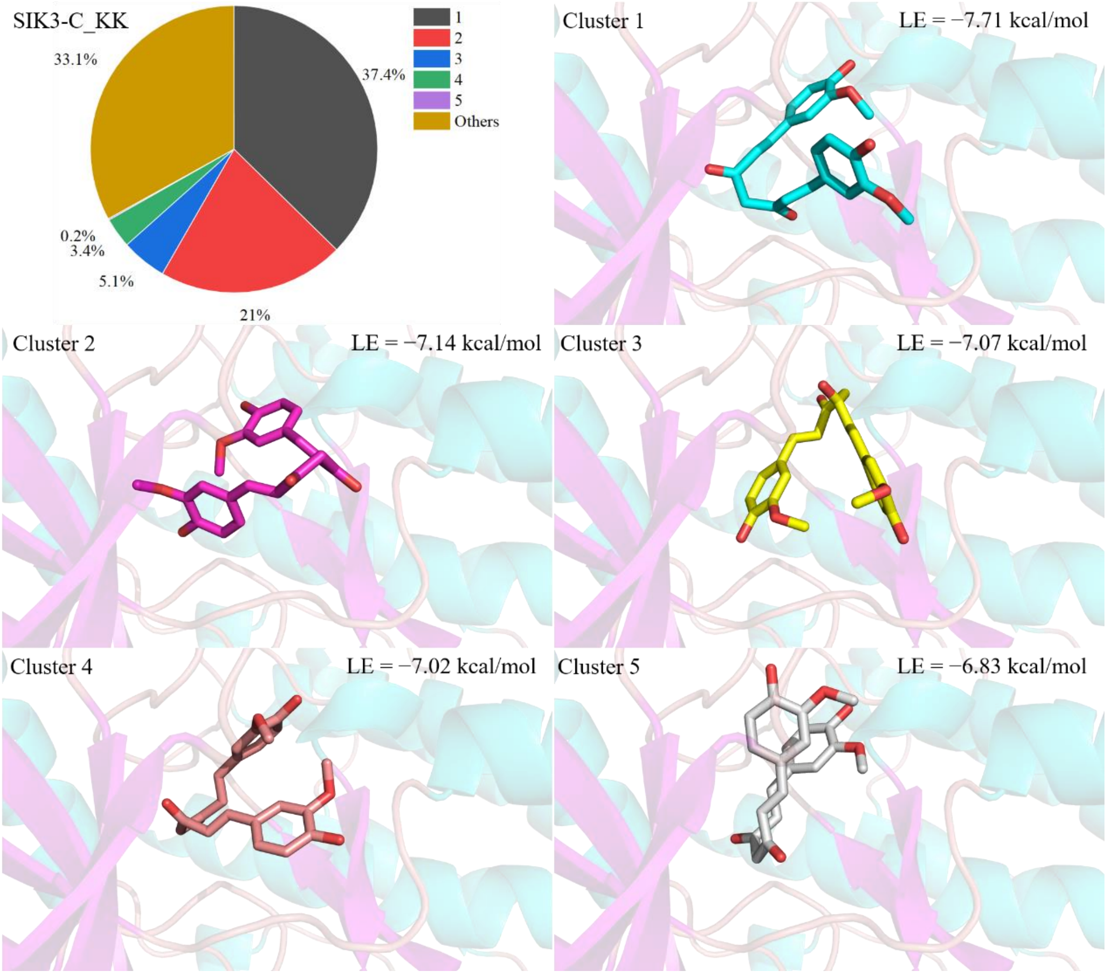

Figure S14. Docking results for curcumin-KK/SIK3-C system.

The cluster analysis result for the 2000 conformations from the docking experiment. The lowest binding energy (LE) was lowest binding energy from the docking score in the same cluster and this conformation was seen as the representative conformation for the cluster. The total number for this docking experiment was set to 2000. The top five clusters for the docking experiment were compared. The speculated binding model for curcumin with the SIK3 took place through hydrogen bonding between the hinge loop of SIK3 and the diketo moiety of curcumin. Thus, the cluster 1 was selected as the possible binding models for diketo form curcumin with SIK3-C.

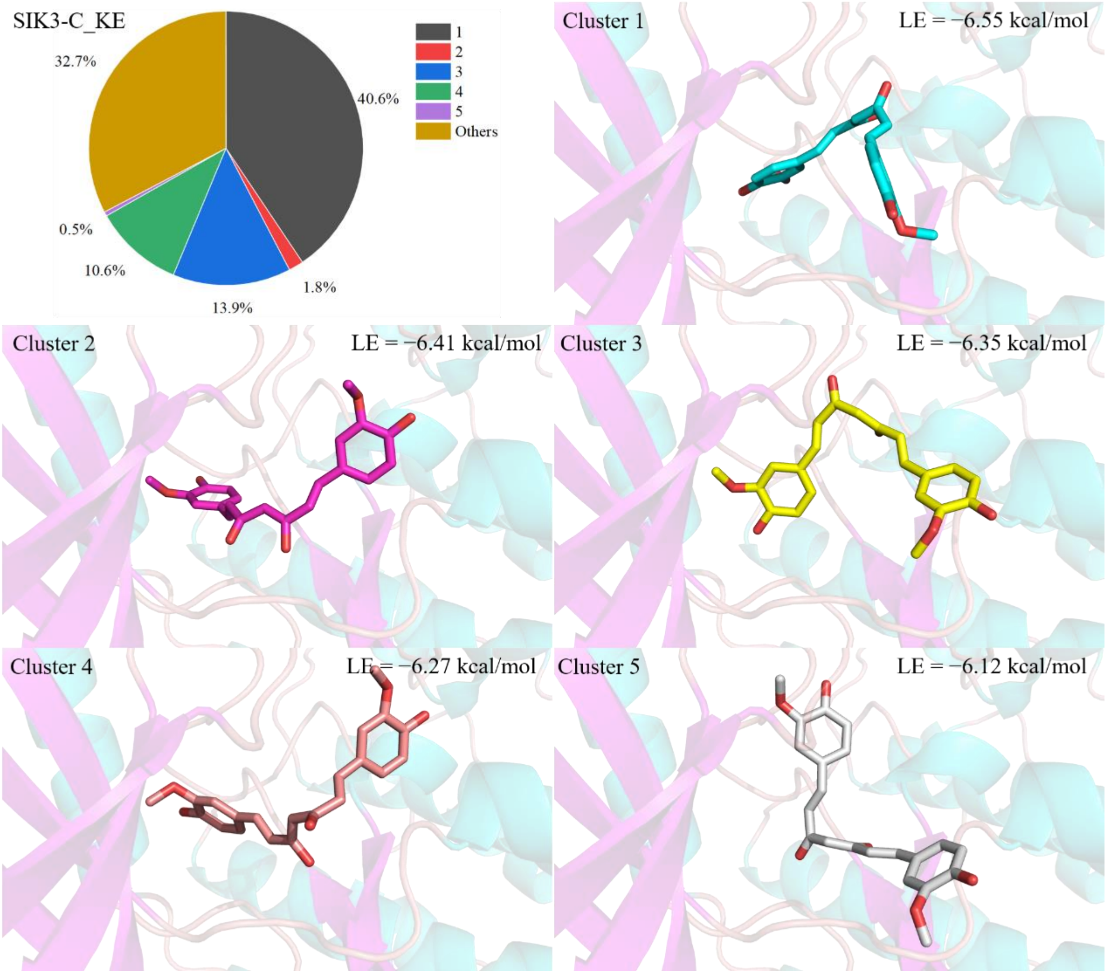

Figure S15. Docking results for curcumin-KE/SIK3-C system.
The cluster analysis result for the 2000 conformations from the docking experiment. The lowest binding energy (LE) was lowest binding energy from the docking score in the same cluster and this conformation was seen as the representative conformation for the cluster. The total number for this docking experiment was set to 2000. The top five clusters for the docking experiment were compared. The speculated binding model for curcumin with the SIK3 took place through hydrogen bonding between the hinge loop of SIK3 and the ketoenol moiety of curcumin. Thus, the cluster 2 was selected as the possible binding models for ketoenol form curcumin with SIK3-C.

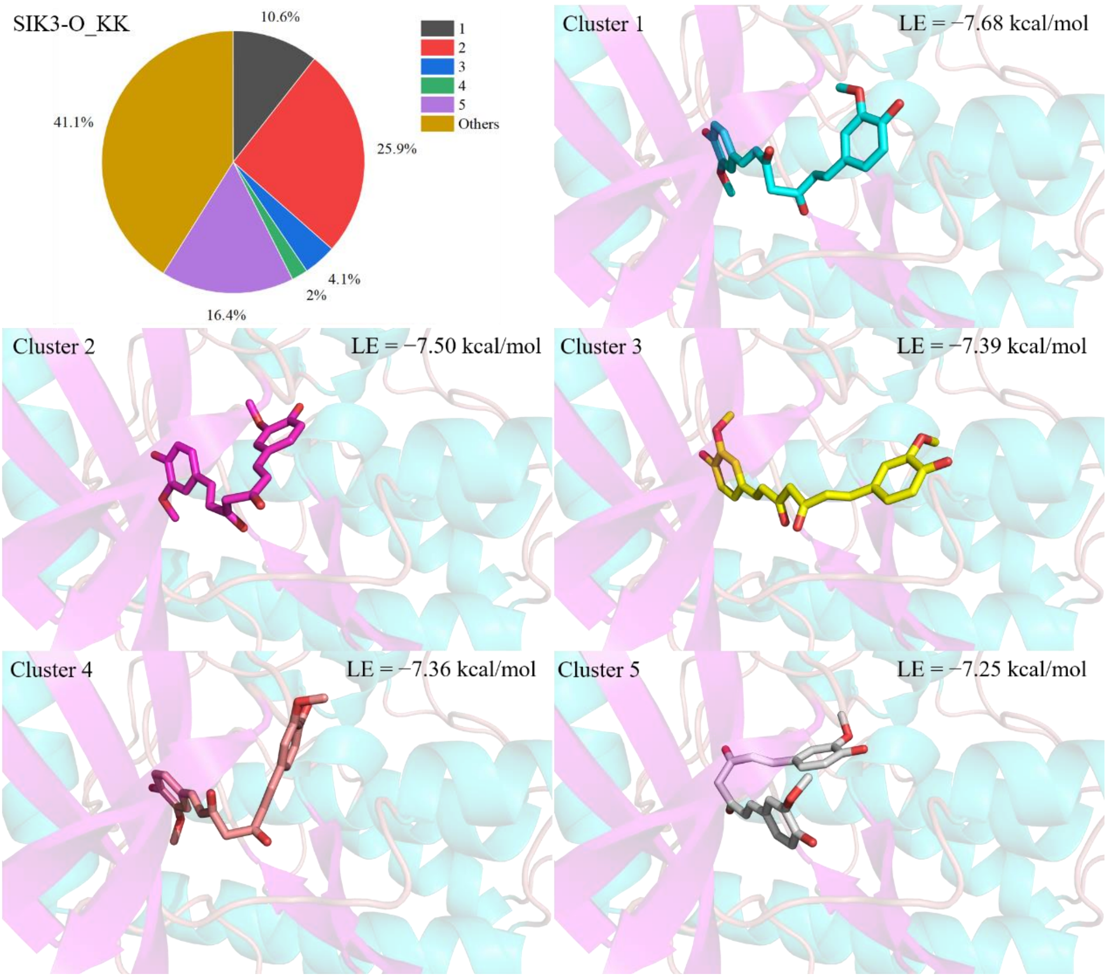

Figure S16. Docking results for curcumin-KK/SIK3-O system.

The cluster analysis result for the 2000 conformations from the docking experiment. The lowest binding energy (LE) was lowest binding energy from the docking score in the same cluster and this conformation was seen as the representative conformation for the cluster. The total number for this docking experiment was set to 2000. The top five clusters for the docking experiment were compared. The speculated binding model for curcumin with the SIK3 took place through hydrogen bonding between the hinge loop of SIK3 and the diketo moiety of curcumin. Thus, the cluster 1 was selected as the possible binding models for diketo form curcumin with SIK3-O.

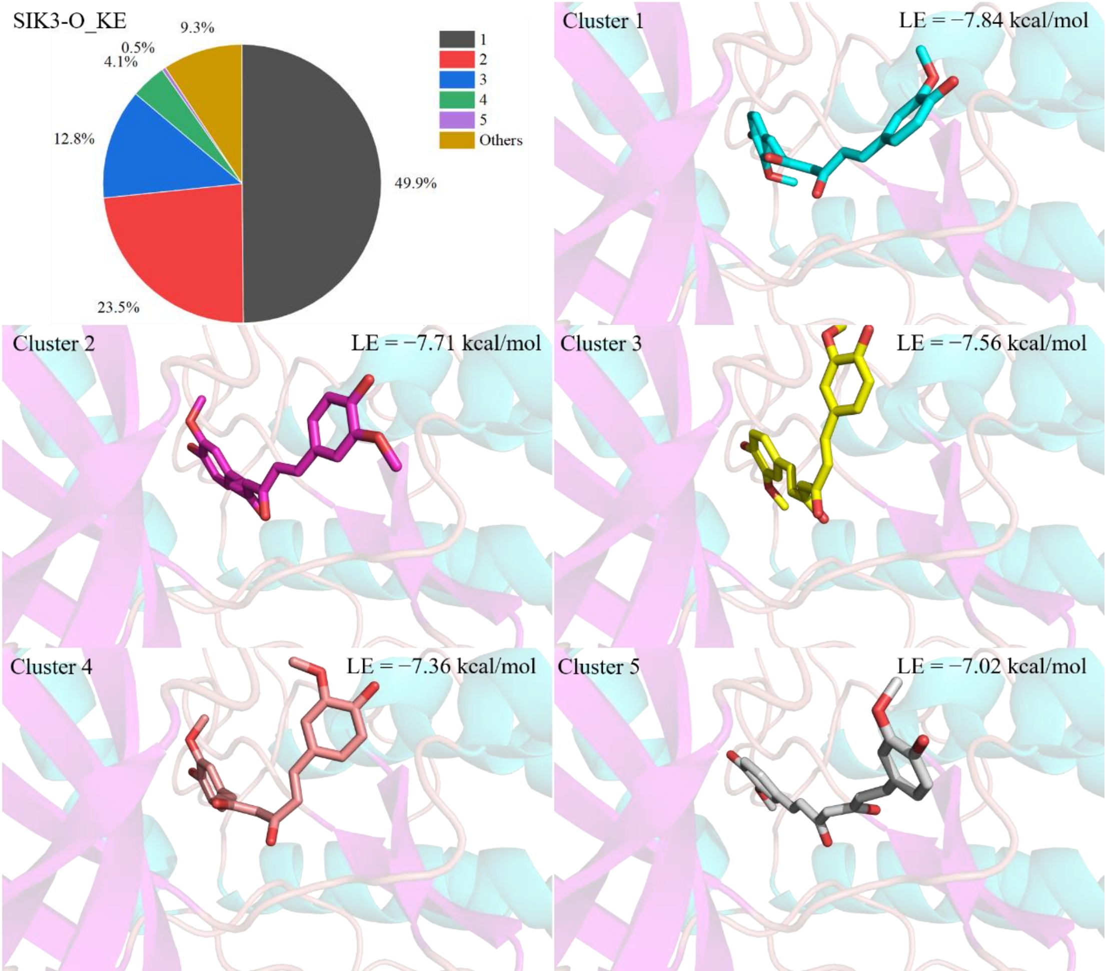

Figure S17. Docking results for curcumin-KE/SIK3-O system.

The cluster analysis result for the 2000 conformations from the docking experiment. The lowest binding energy (LE) was lowest binding energy from the docking score in the same cluster and this conformation was seen as the representative conformation for the cluster. The total number for this docking experiment was set to 2000. The top five clusters for the docking experiment were compared. The speculated binding model for curcumin with the SIK3 took place through hydrogen bonding between the hinge loop of SIK3 and the ketoenol moiety of curcumin. Thus, the cluster 1 was selected as the possible binding models for ketoenol form curcumin with SIK3-O.

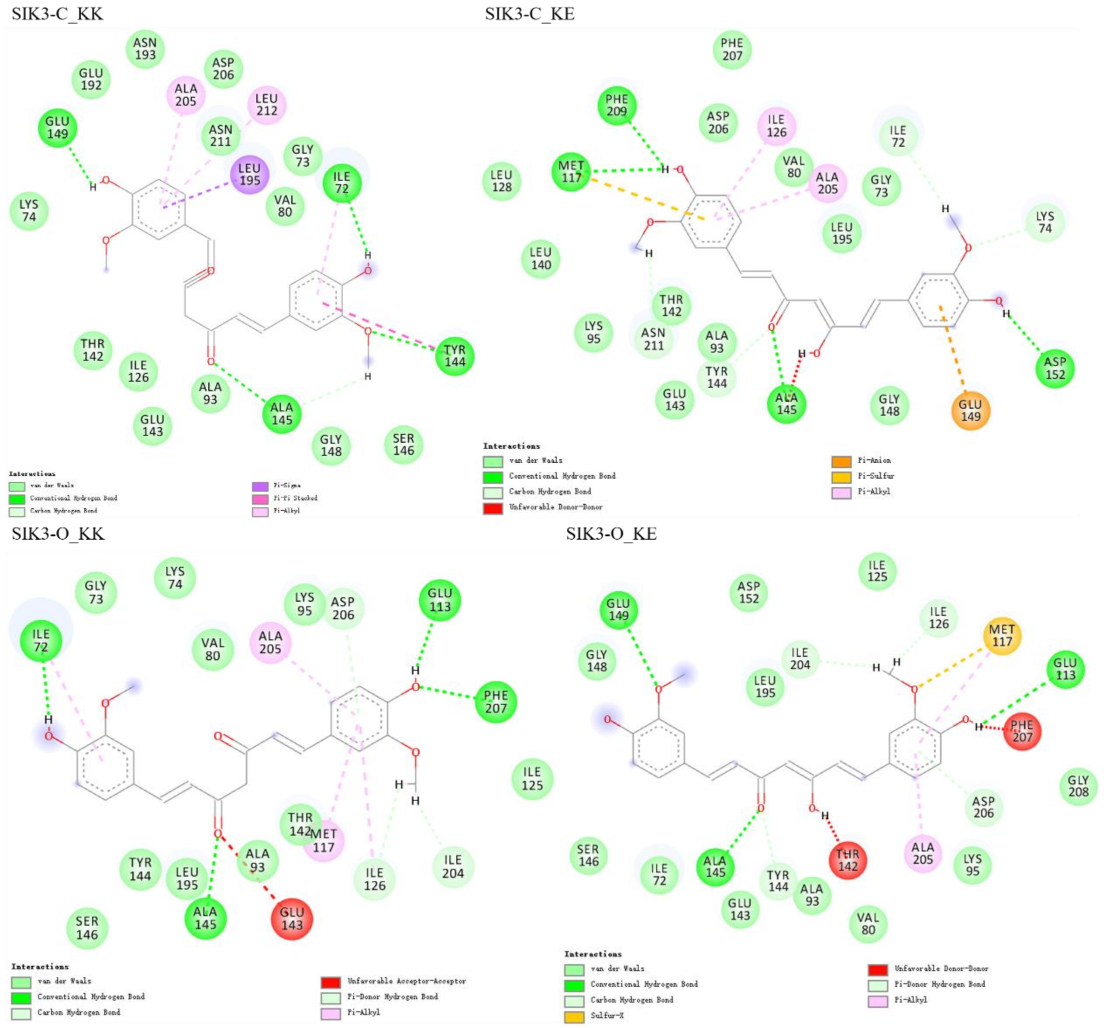

Figure S18. Putative binding mode of inhibitor curcumin in the kinase domain of SIK3. The 2D interaction models were drawn by Discovery Studio 2017 R2 for the selected conformation of curcumin binding with and human SIK3 from molecule docking results.

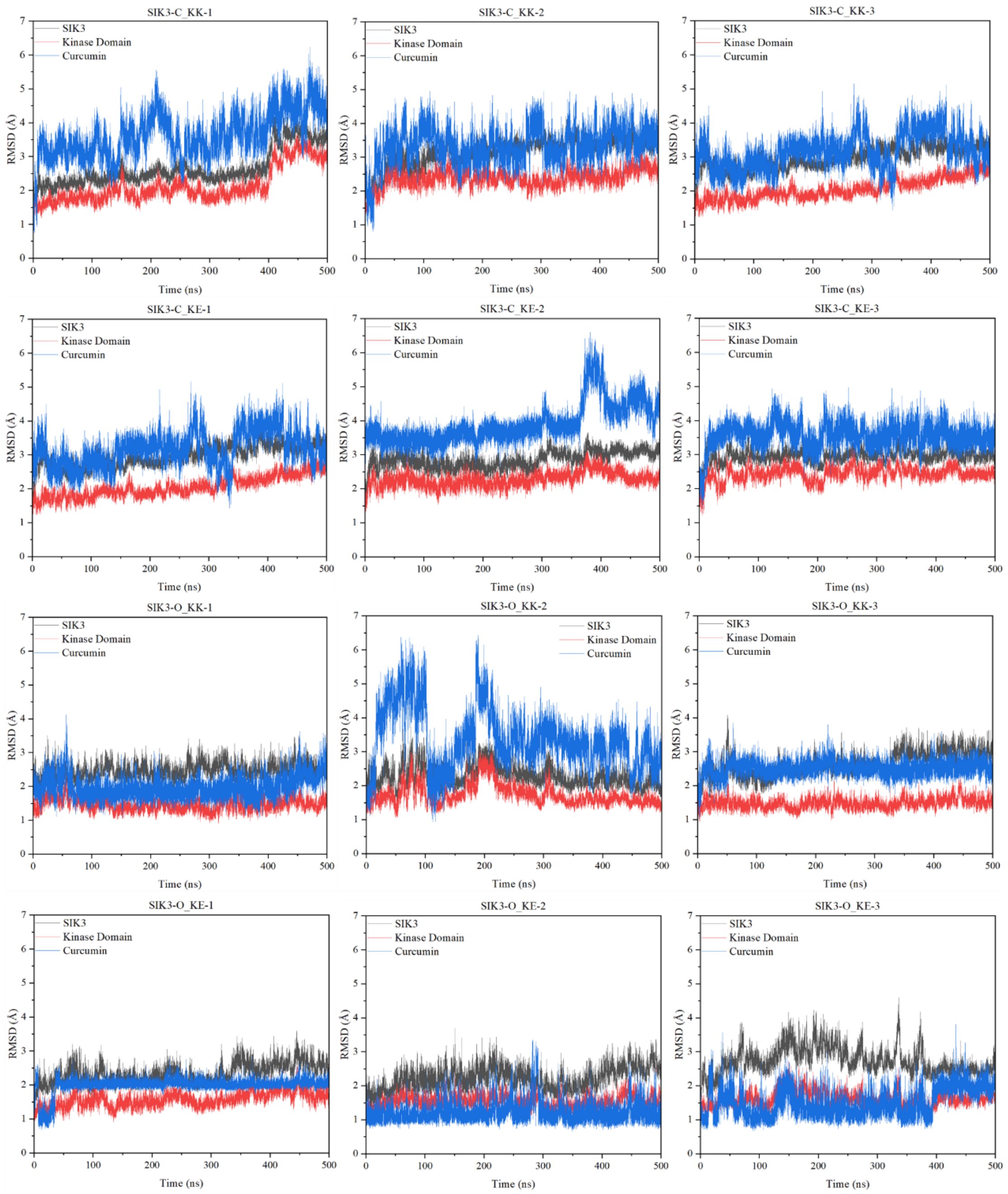

Figure S19. Root mean square deviation (RMSD) value of heavy atoms of backbone for protein receptor SIK3 and of the ligand curcumin along 500 ns MD simulation for **curcumin/SIK3** systems.

The SIK3 and Kinase Domain indicate the RMSD value of heavy atoms of backbone for the residues of SIK3 and kinase domain residues of SIK3, respectively; and curcumin displays the RMSD value of heavy atoms of curcumin. SIK3-C indicates the closed conformation of T-loop for SIK3 protein and SIK3-O for open conformation. KK indicates for the diketo form of curcumin and KE for ketoenol form curcumin.

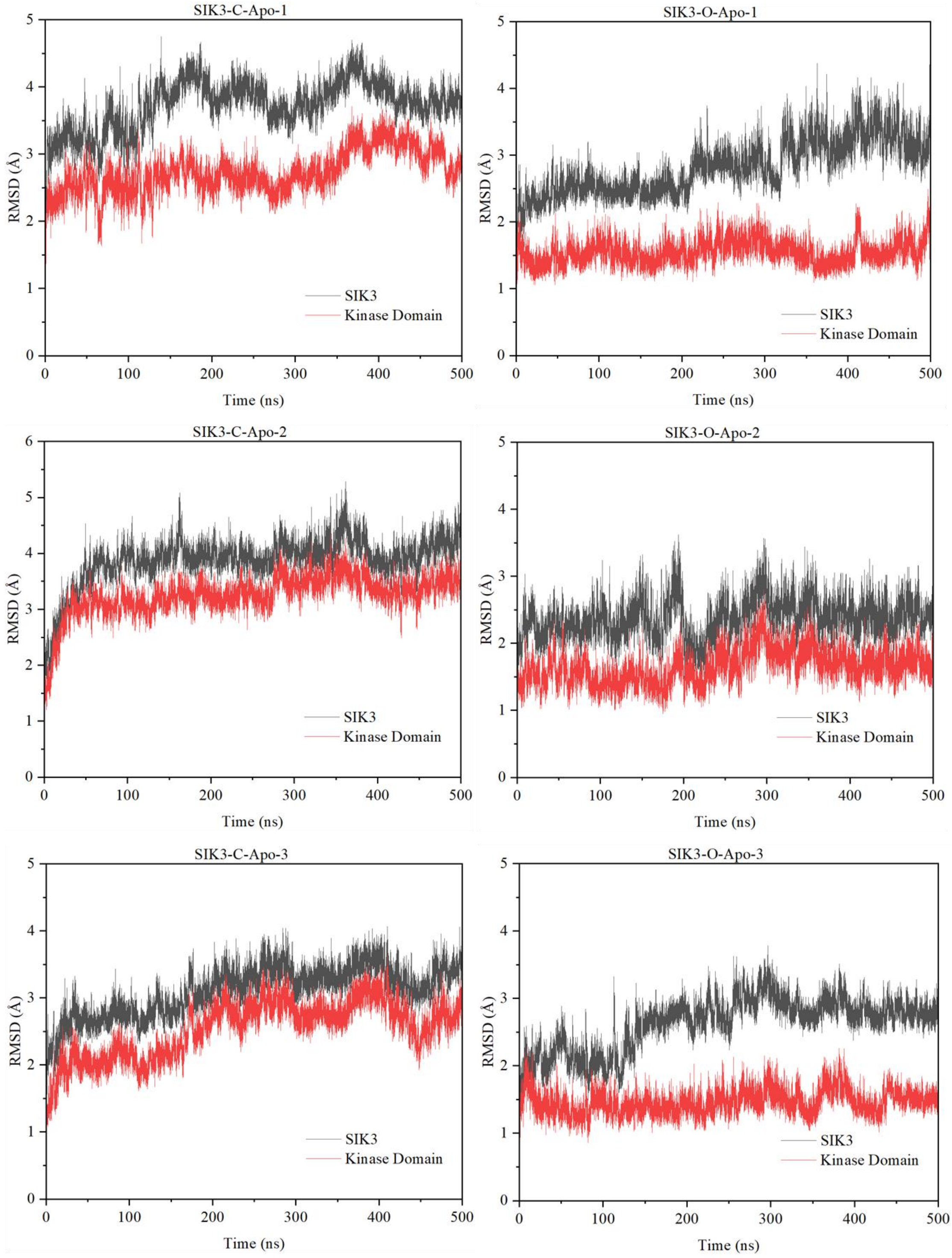

Figure S20. Root mean square deviation (RMSD) value of heavy atoms of backbone for protein receptor SIK3 along 500 ns MD simulation for **SIK3-Apo** systems.
The SIK3 indicates the RMSD value of heavy atoms of backbone for the residues of SIK3 and Kinase Domain shows the RMSD value of heavy atoms of backbone for kinase domain residues of SIK3. SIK3-C indicates the closed conformation of T-loop for SIK3 protein and SIK3-O for open conformation.

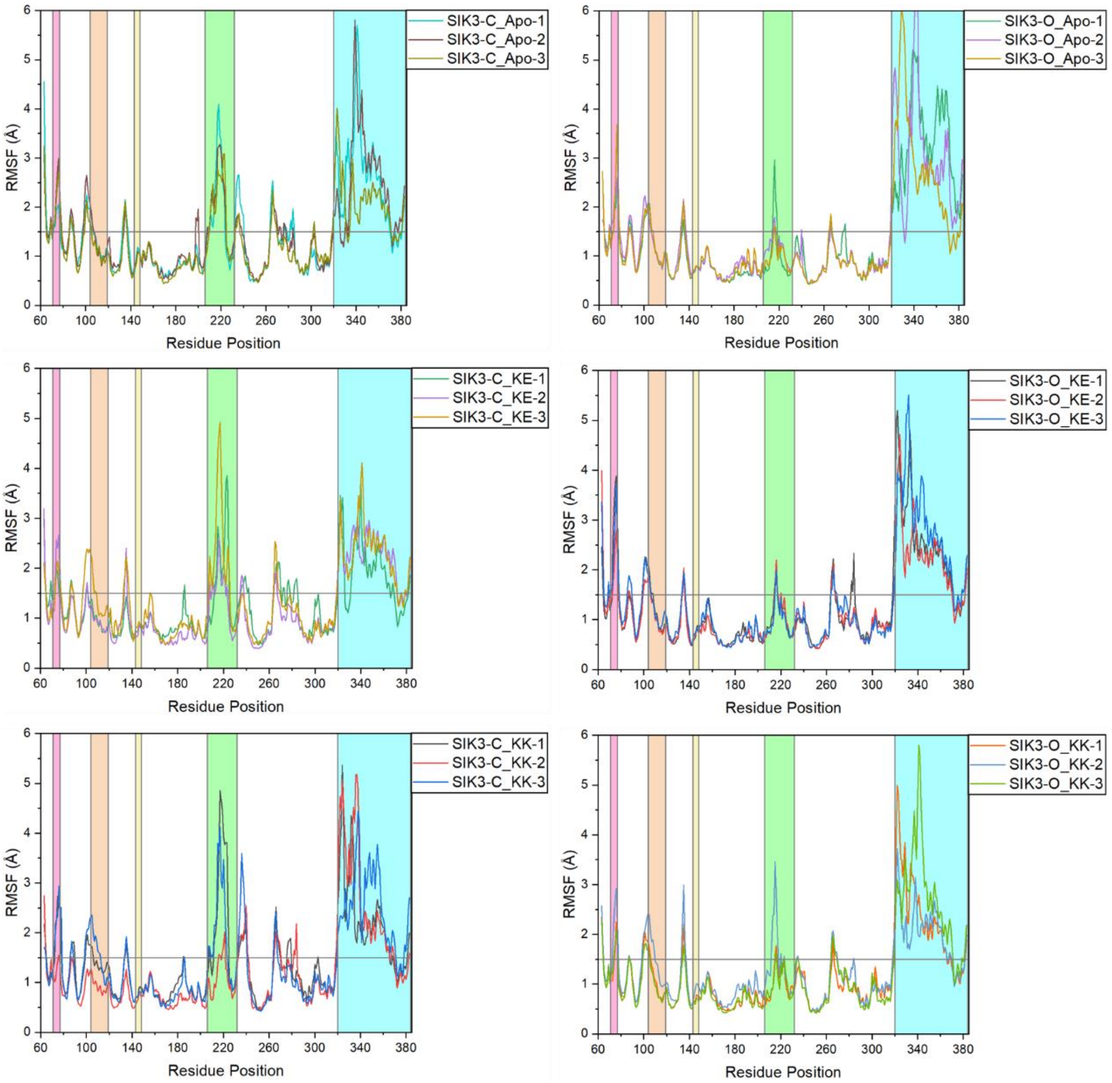

Figure S21. RMSF variations for Cα atom of SIK3 for Curcumin/SIK3 and Apo-SIK3 systems from the 500 ns MD simulation.

SIK3-C indicates the closed conformation of T-loop for SIK3 protein and SIK3-O for open conformation. SIK3-Apo indicates the system without curcumin molecule.

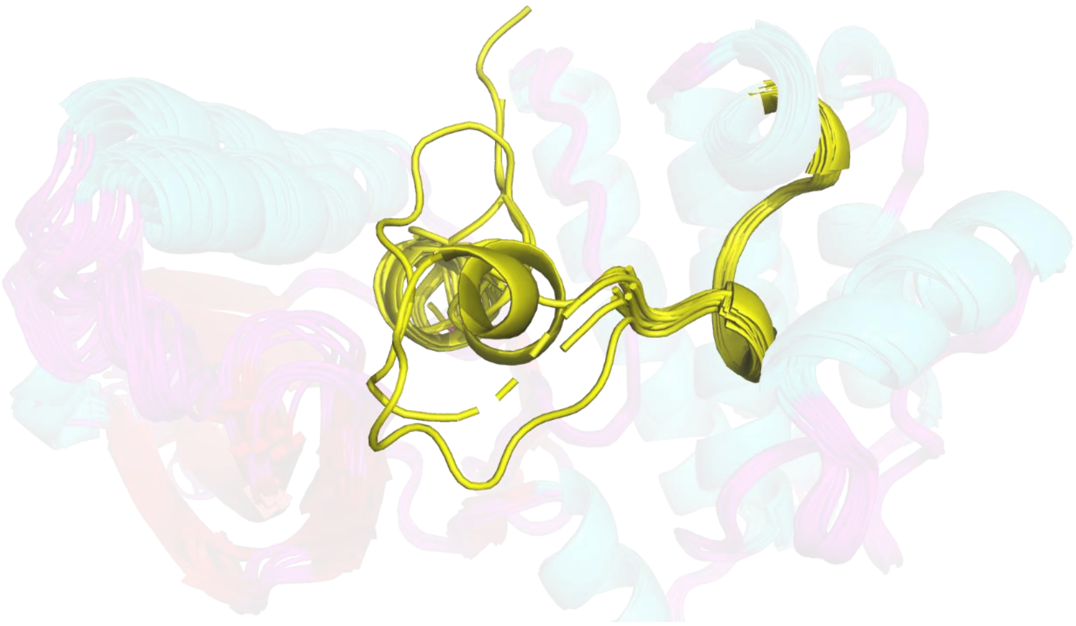

Figure S22. Conformation of human FAK binding with inhibitors.
The crystal complex structures obtained from the Protein Database Bank (November 22, 2022). The T-loop was shown with yellow cartoon.

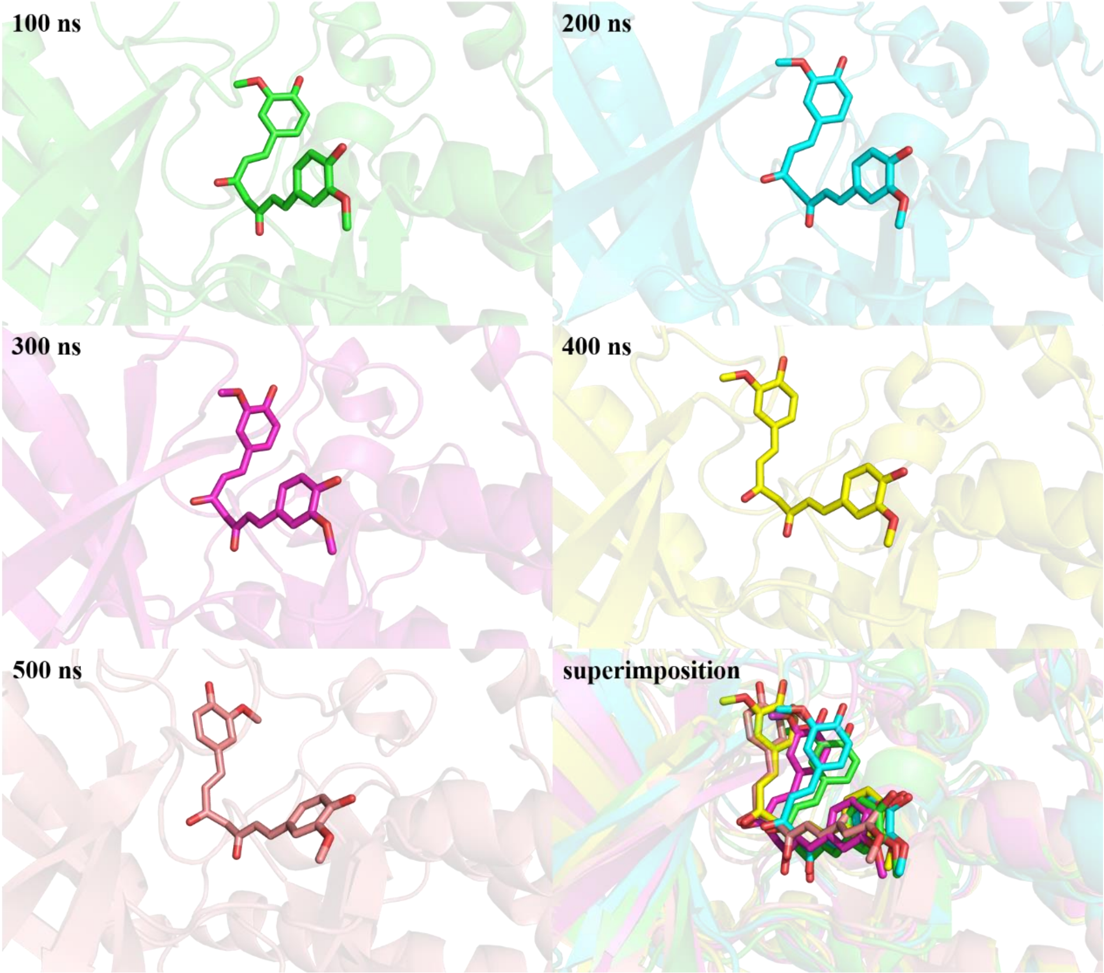

Figure S23. Snapshots of the SIK3-C-KK-1 along the dynamic simulation time for 100, 200, 300, 400, and 500 ns.
For clarity, the water molecules have been removed. The inhibitor is plotted using stick style, while cartoon style for SIK3.

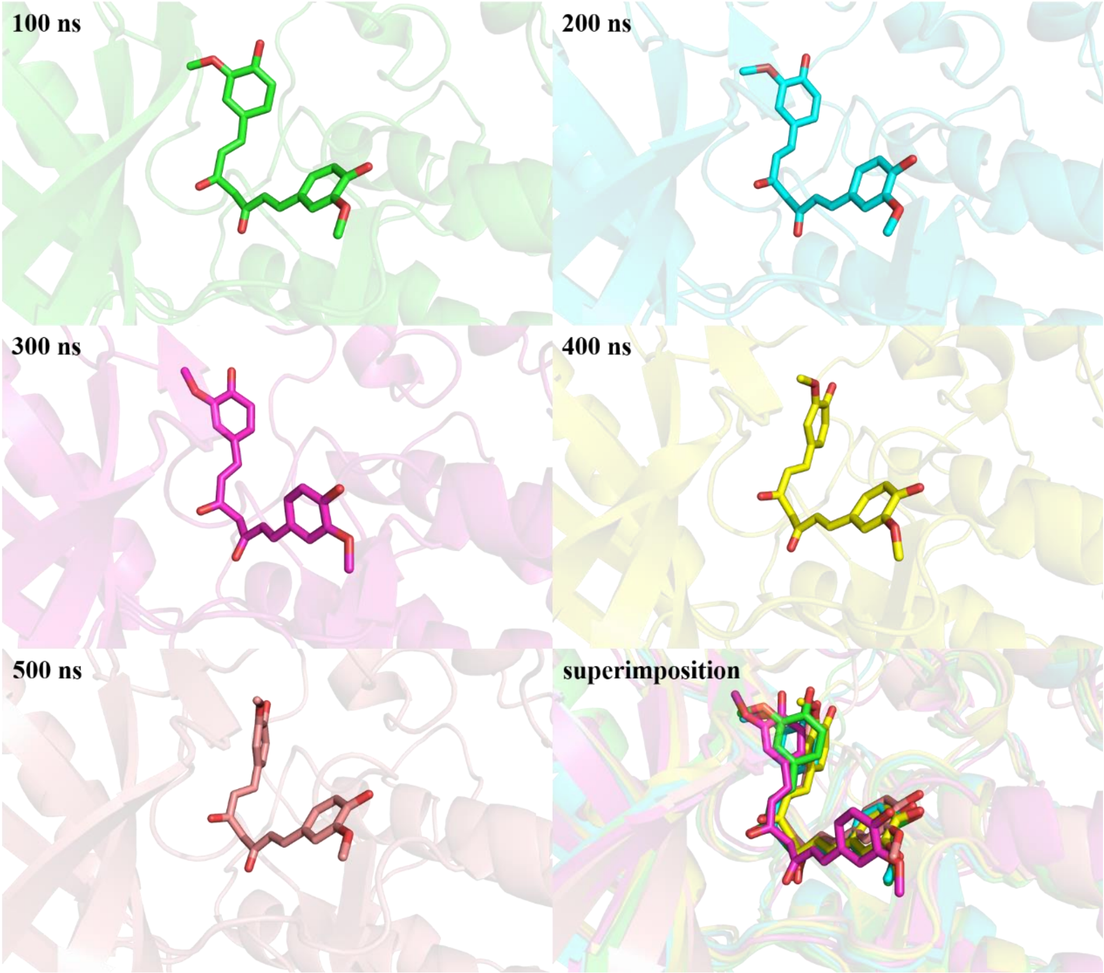

Figure S24. Snapshots of the SIK3-C-KK-2 along the dynamic simulation time for 100, 200, 300, 400, and 500 ns.

For clarity, the water molecules have been removed. The inhibitor is plotted using stick style, while cartoon style for SIK3.

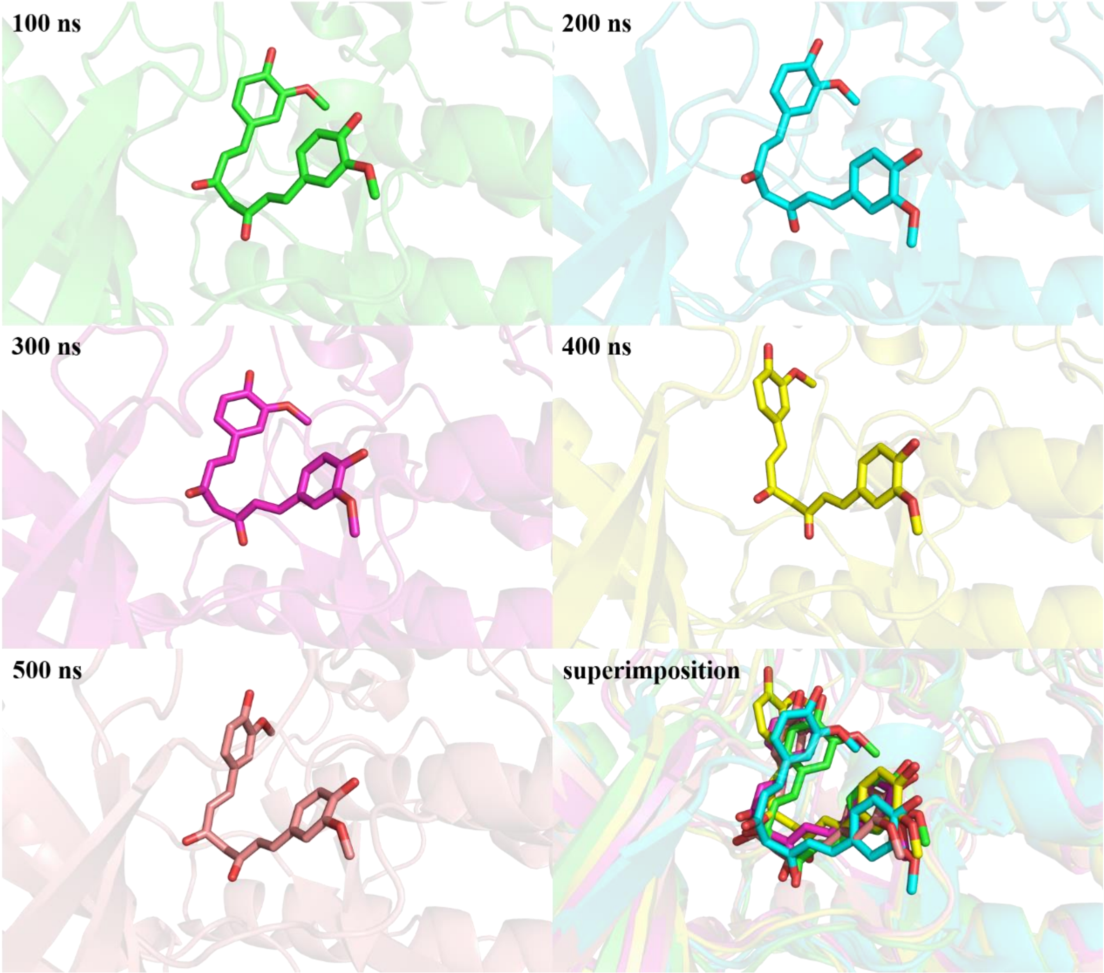

Figure S25. Snapshots of the SIK3-C-KK-3 along the dynamic simulation time for 100, 200, 300, 400, and 500 ns.
For clarity, the water molecules have been removed. The inhibitor is plotted using stick style, while cartoon style for SIK3.

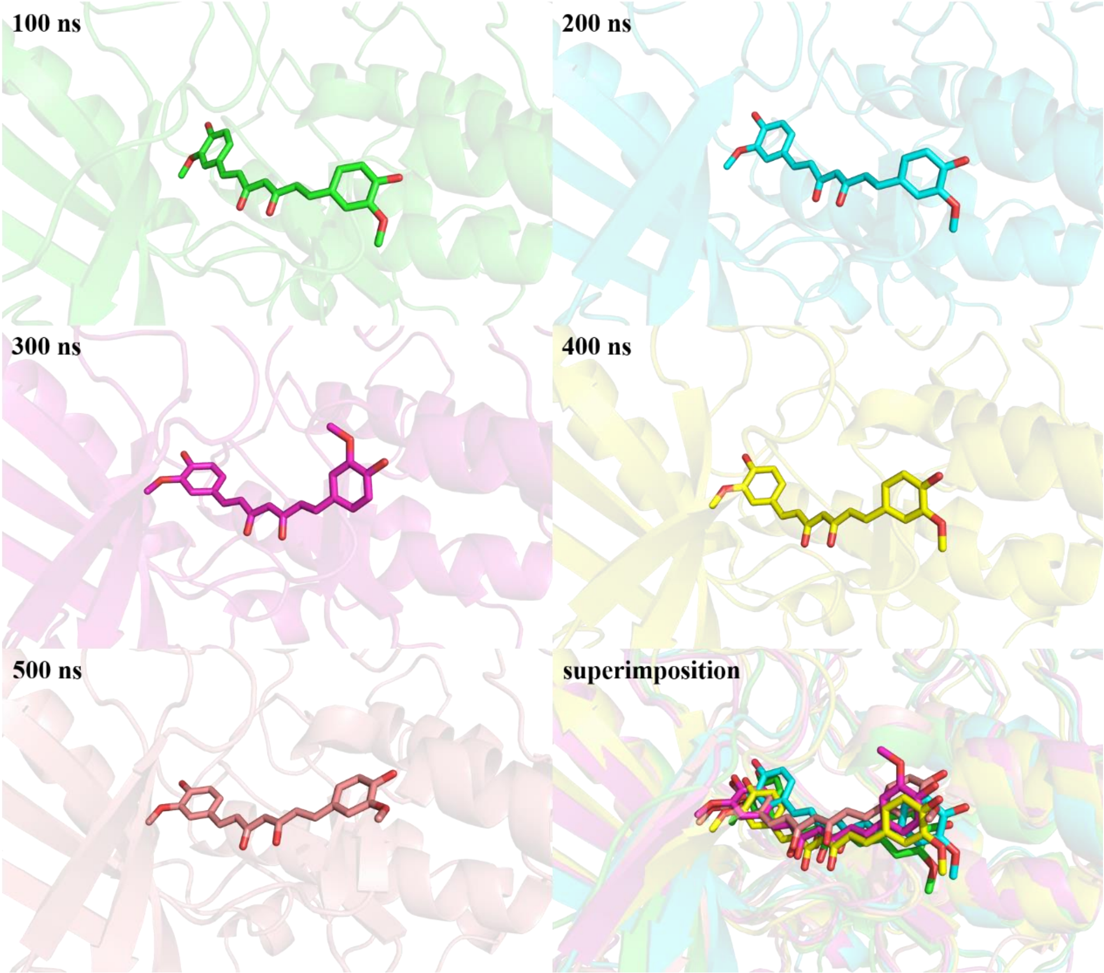

Figure S26. Snapshots of the SIK3-C-KE-1 along the dynamic simulation time for 100, 200, 300, 400, and 500 ns.
For clarity, the water molecules have been removed. The inhibitor is plotted using stick style, while cartoon style for SIK3.

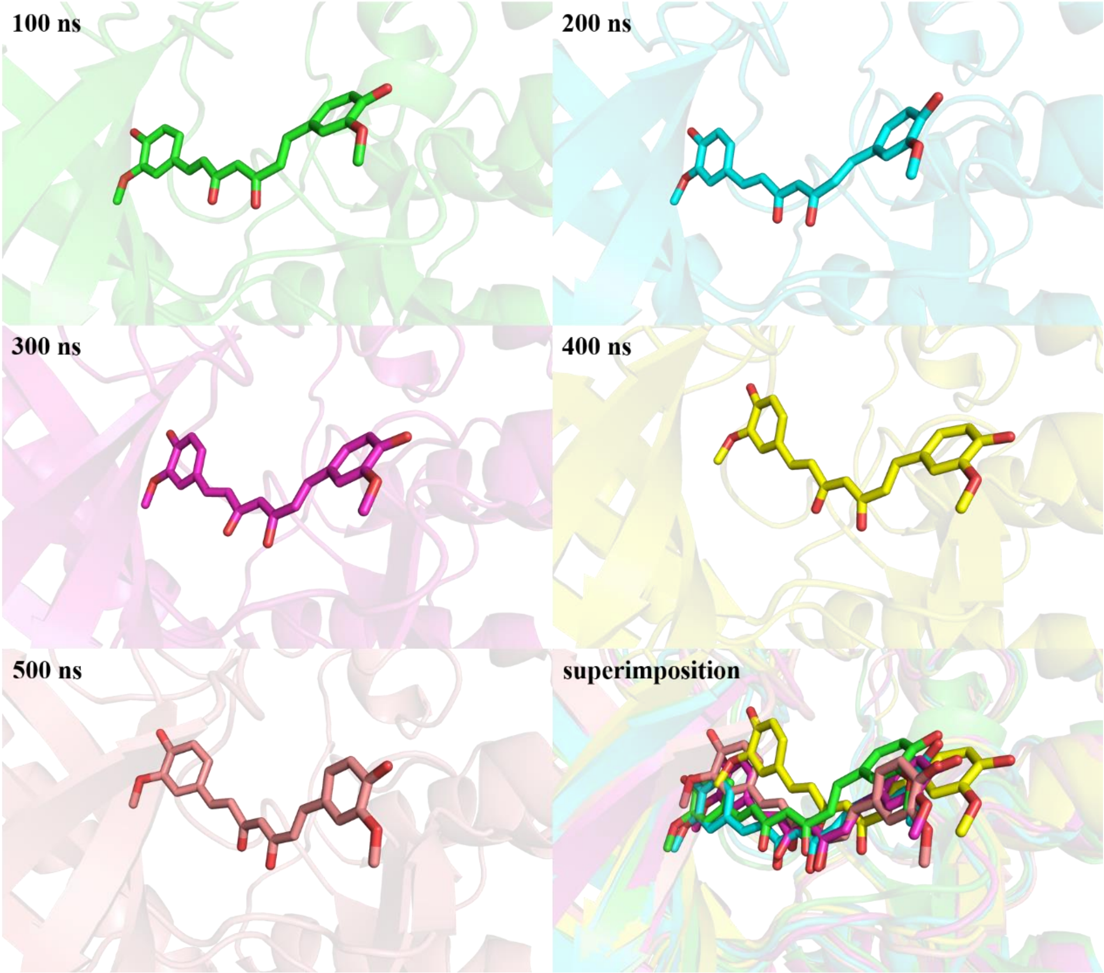

Figure S27. Snapshots of the SIK3-C-KE-2 along the dynamic simulation time for 100, 200, 300, 400, and 500 ns.
For clarity, the water molecules have been removed. The inhibitor is plotted using stick style, while cartoon style for SIK3.

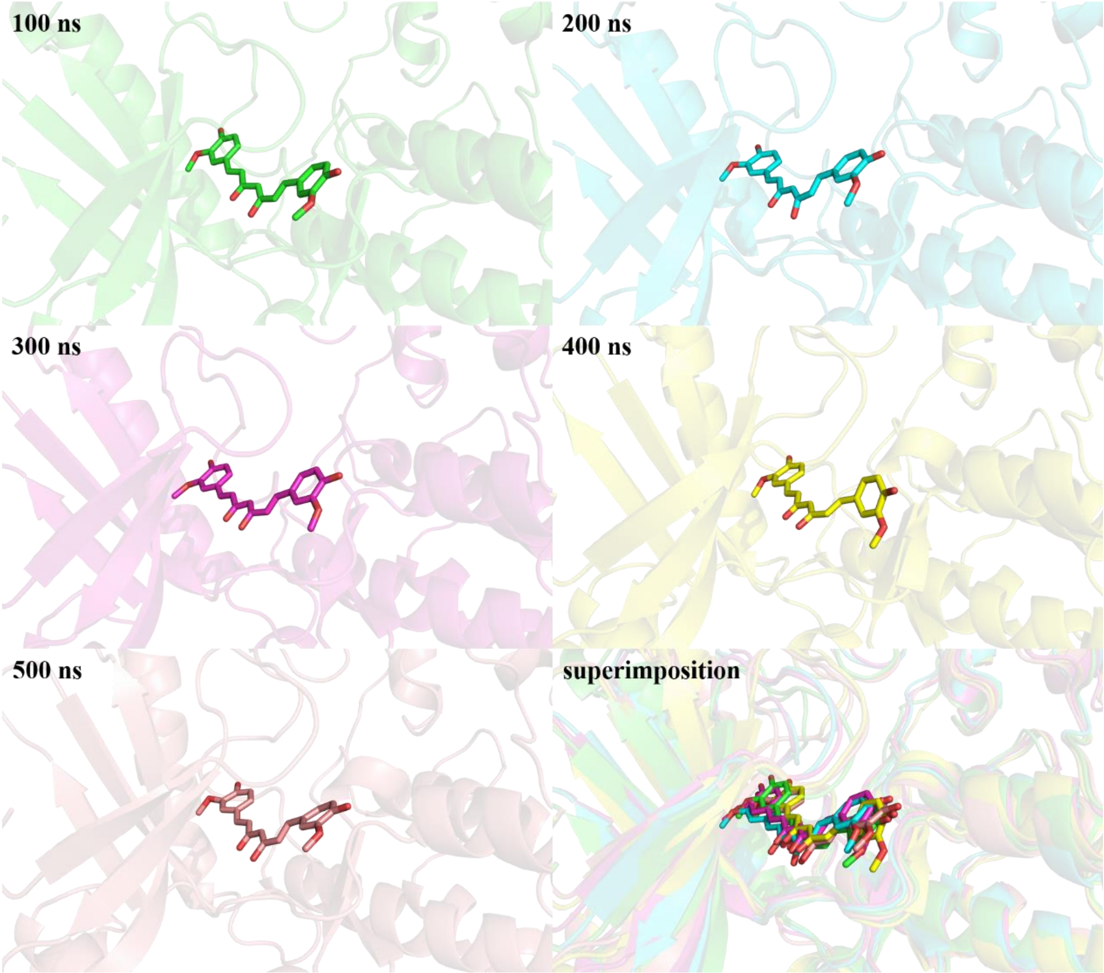

Figure S28. Snapshots of the SIK3-C-KE-3 along the dynamic simulation time for 100, 200, 300, 400, and 500 ns.
For clarity, the water molecules have been removed. The inhibitor is plotted using stick style, while cartoon style for SIK3.

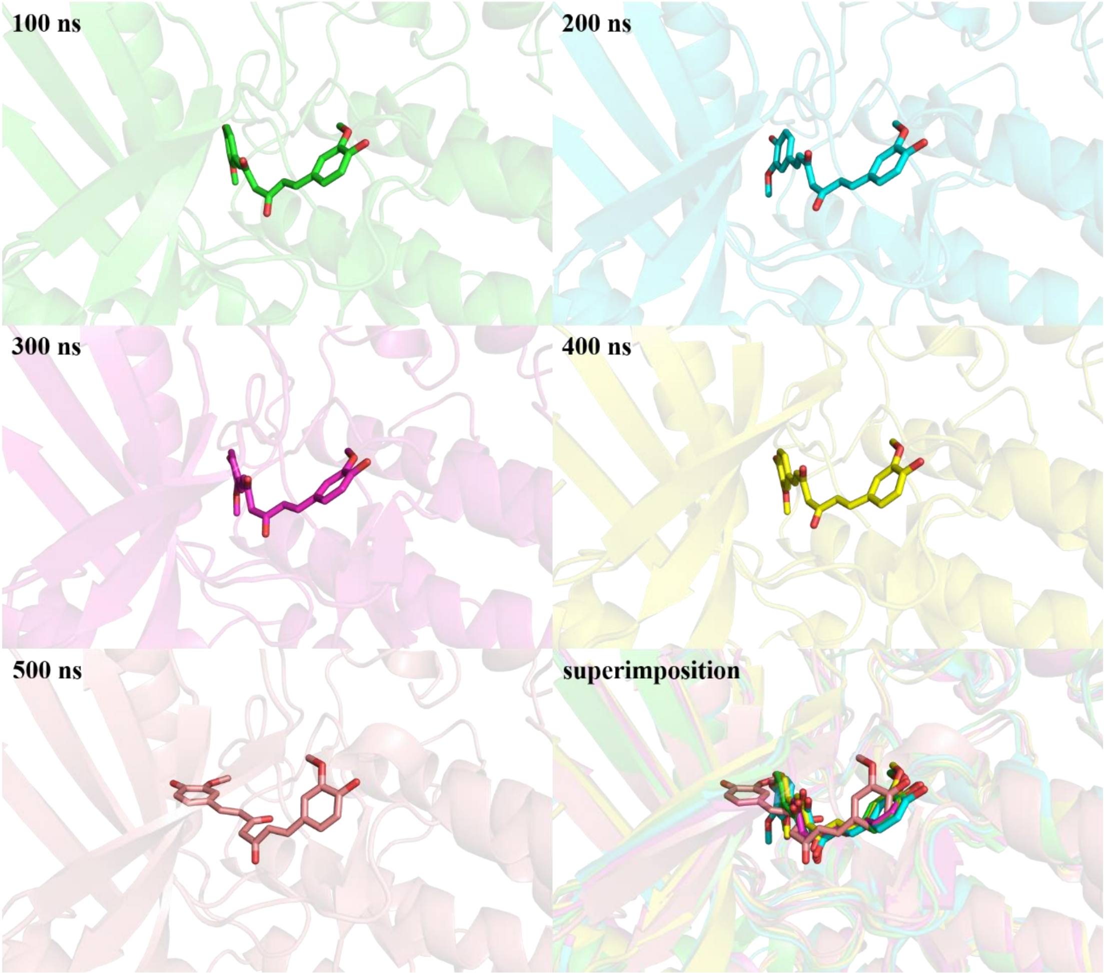

Figure S29. Snapshots of the SIK3-O-KK-1 along the dynamic simulation time for 100, 200, 300, 400, and 500 ns.
For clarity, the water molecules have been removed. The inhibitor is plotted using stick style, while cartoon style for SIK3.

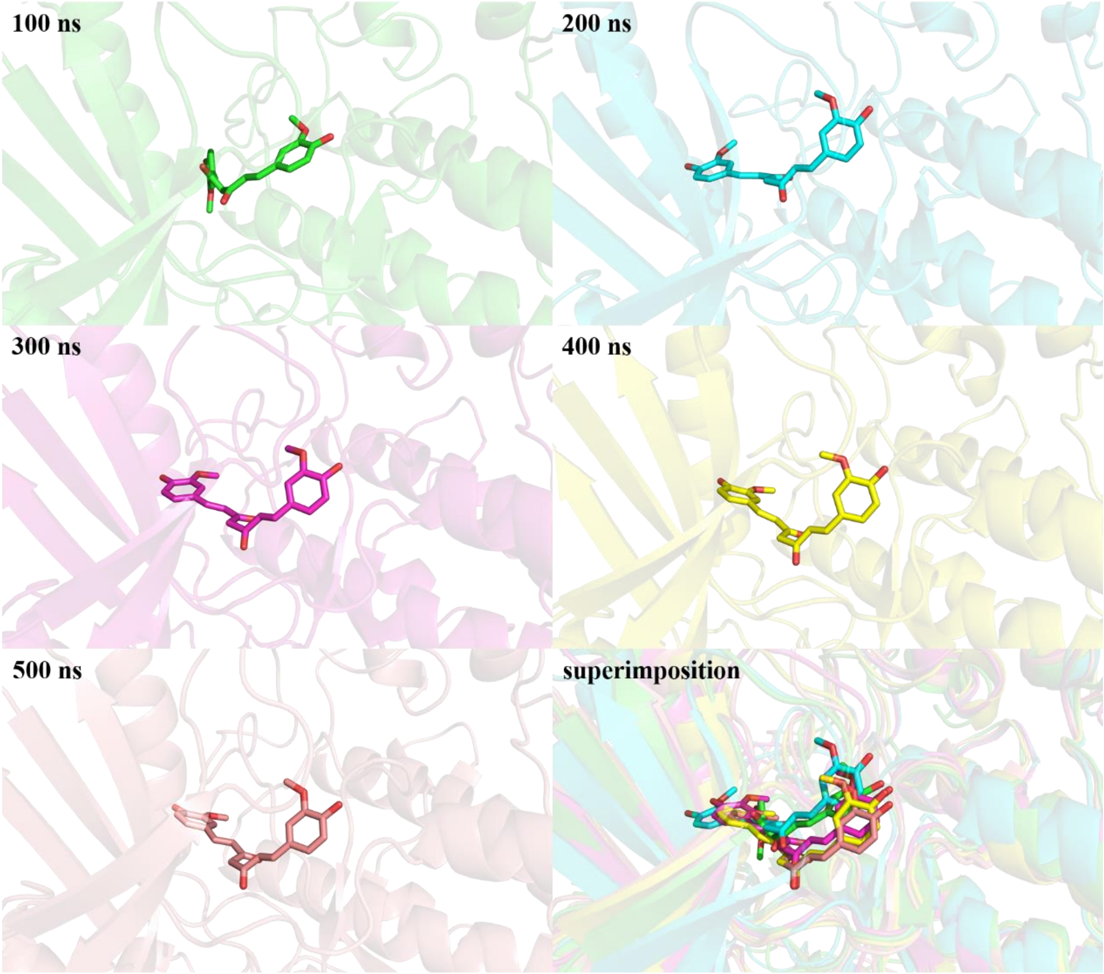

Figure S30. Snapshots of the SIK3-O-KK-2 along the dynamic simulation time for 100, 200, 300, 400, and 500 ns.
For clarity, the water molecules have been removed. The inhibitor is plotted using stick style, while cartoon style for SIK3.

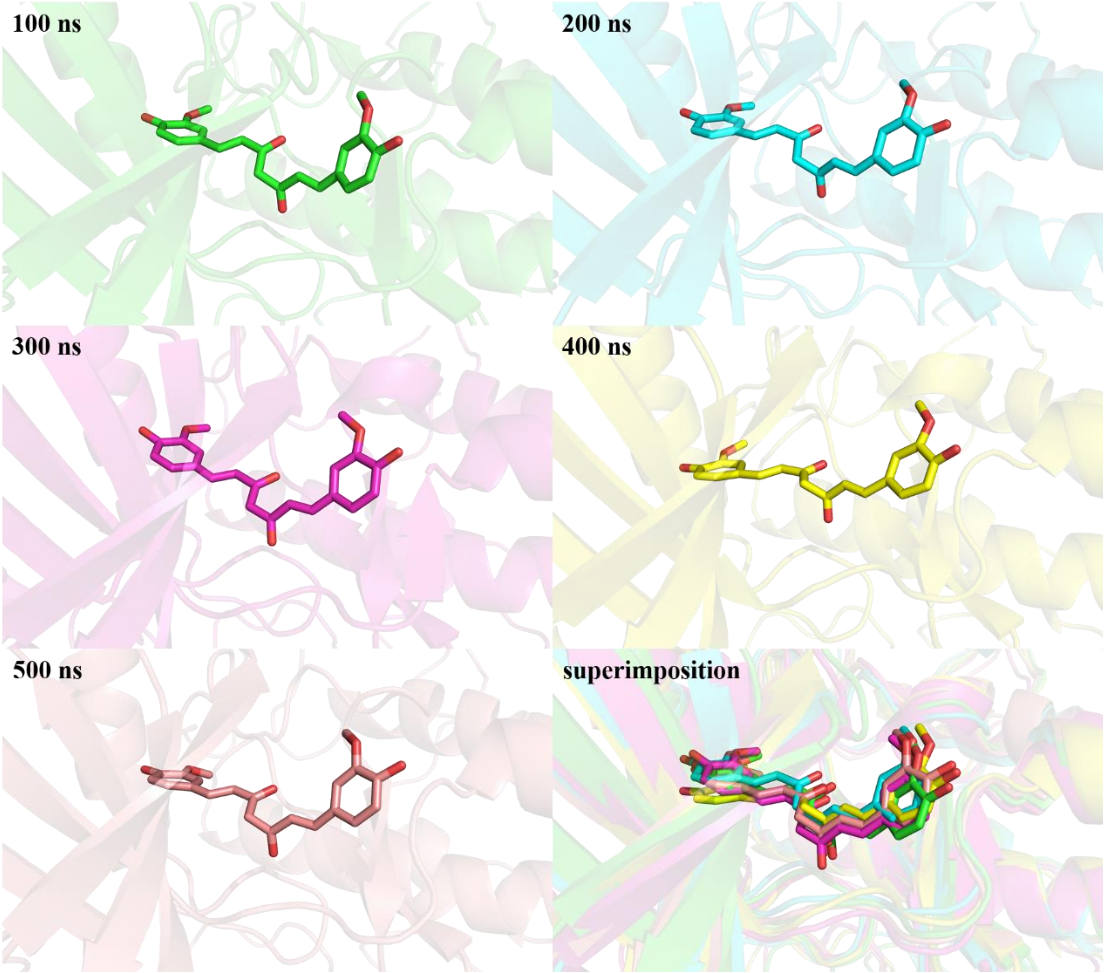

Figure S31. Snapshots of the SIK3-O-KK-3 along the dynamic simulation time for 100, 200, 300, 400, and 500 ns.
For clarity, the water molecules have been removed. The inhibitor is plotted using stick style, while cartoon style for SIK3.

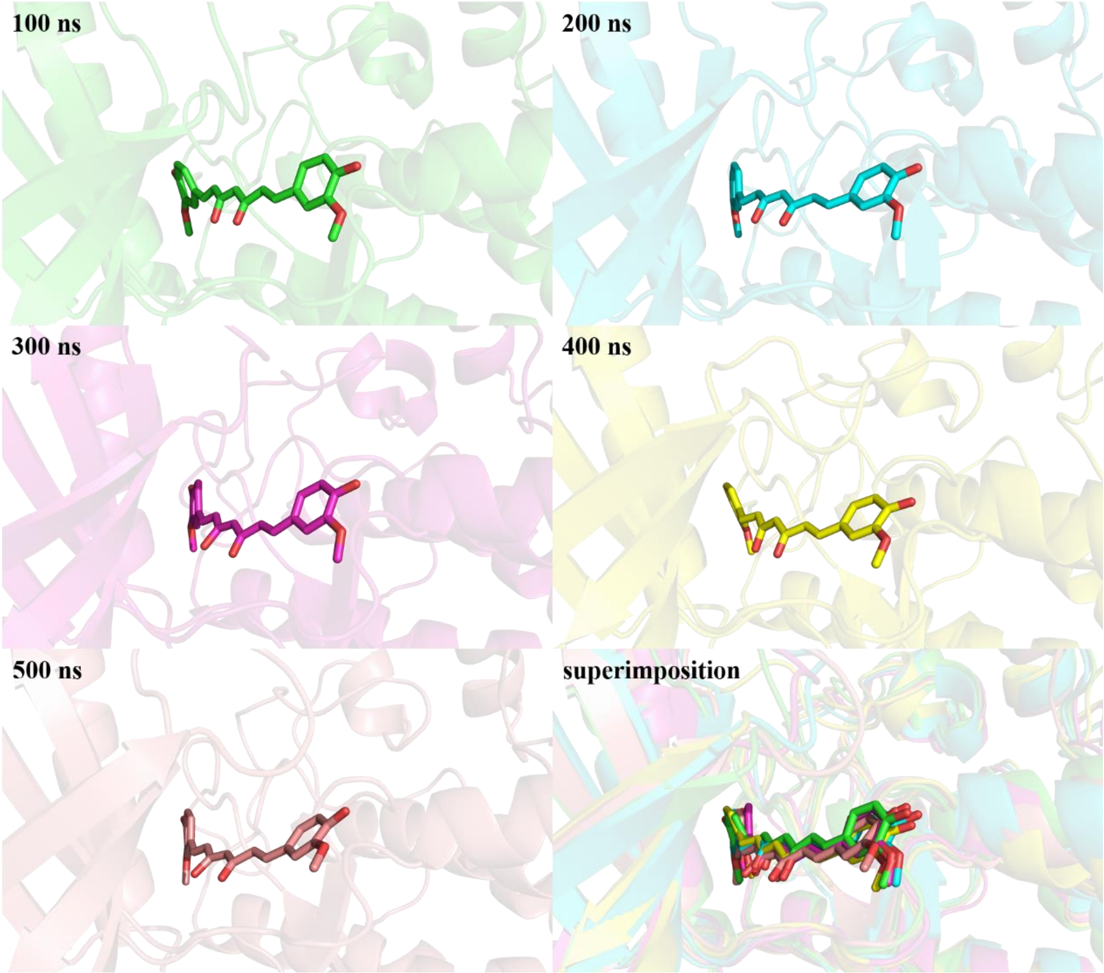

Figure S32. Snapshots of the SIK3-O-KE-1 along the dynamic simulation time for 100, 200, 300, 400, and 500 ns.
For clarity, the water molecules have been removed. The inhibitor is plotted using stick style, while cartoon style for SIK3.

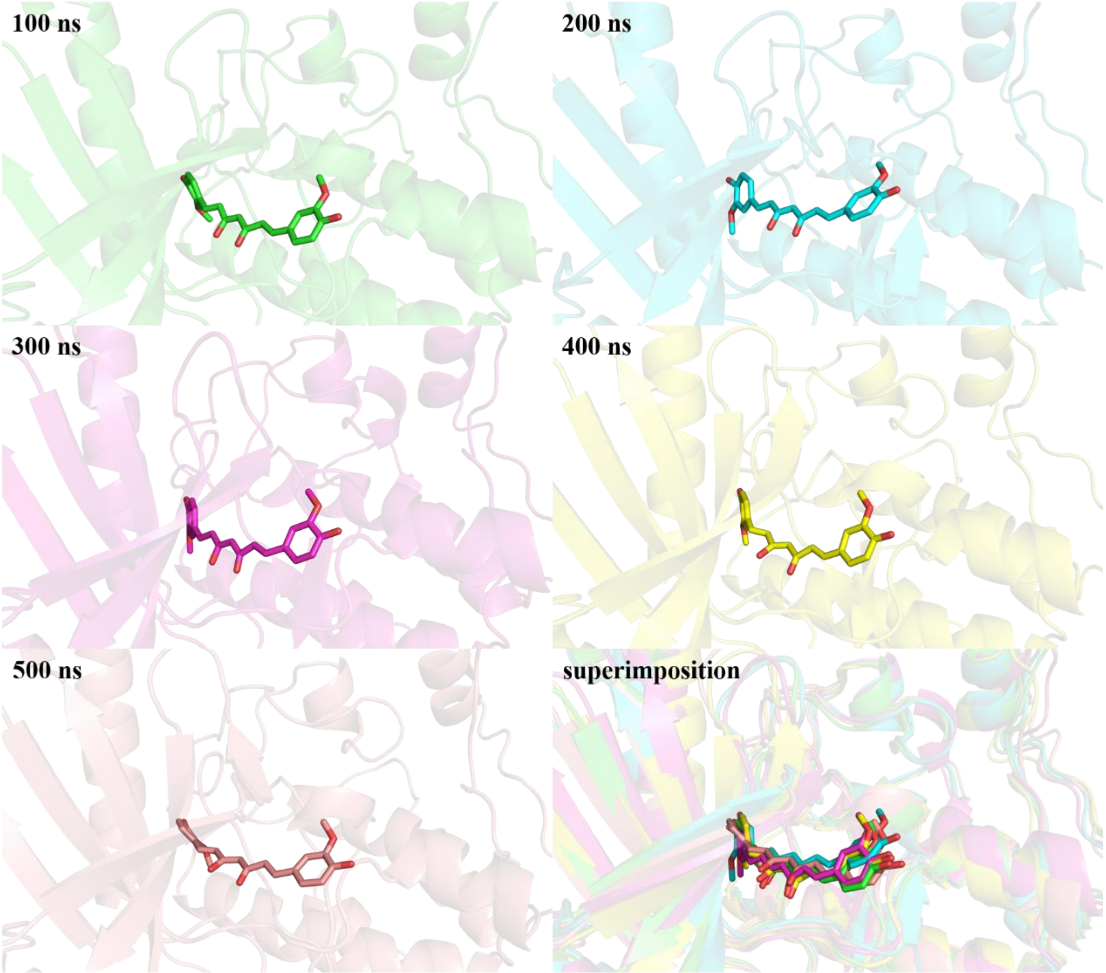

Figure S33. Snapshots of the SIK3-O-KE-2 along the dynamic simulation time for 100, 200, 300, 400, and 500 ns.
For clarity, the water molecules have been removed. The inhibitor is plotted using stick style, while cartoon style for SIK3.

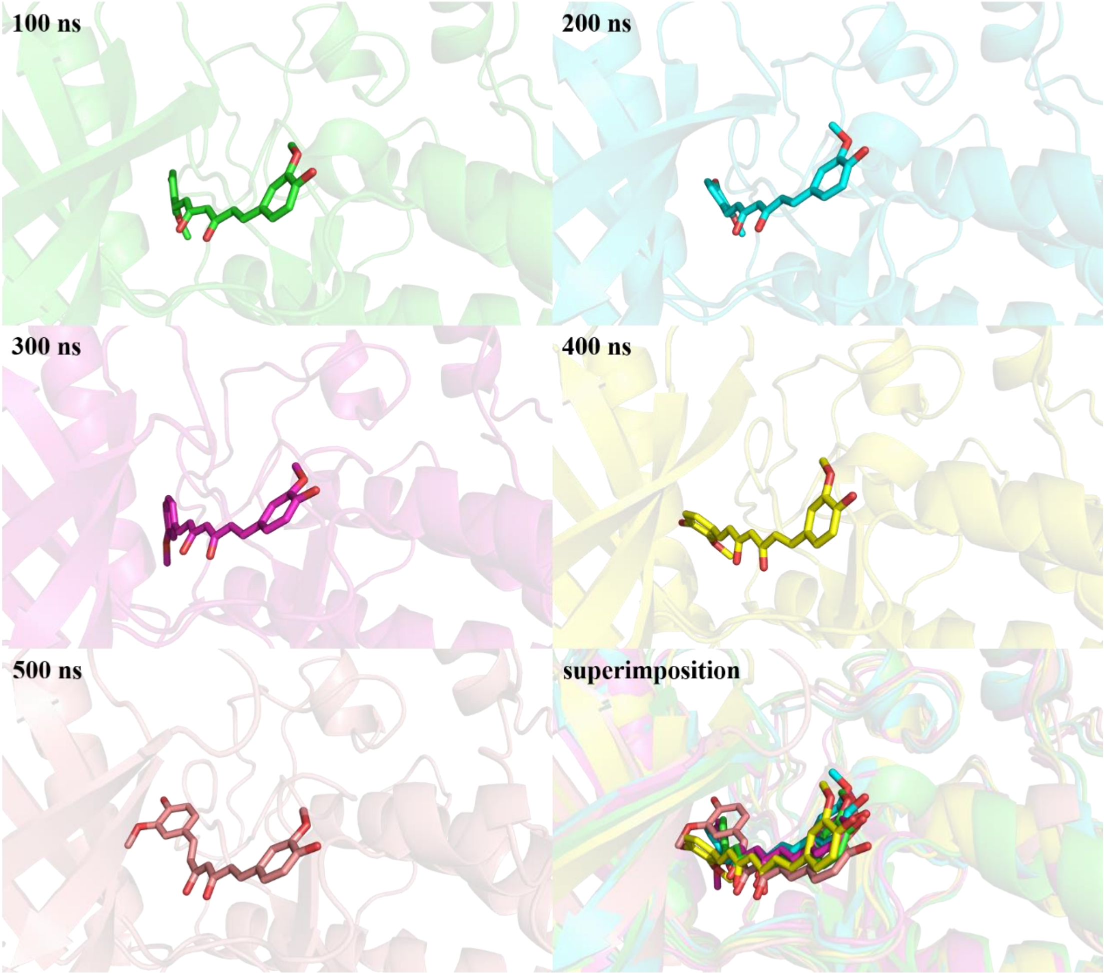

Figure S34. Snapshots of the SIK3-O-KE-3 along the dynamic simulation time for 100, 200, 300, 400, and 500 ns.
For clarity, the water molecules have been removed. The inhibitor is plotted using stick style, while cartoon style for SIK3.

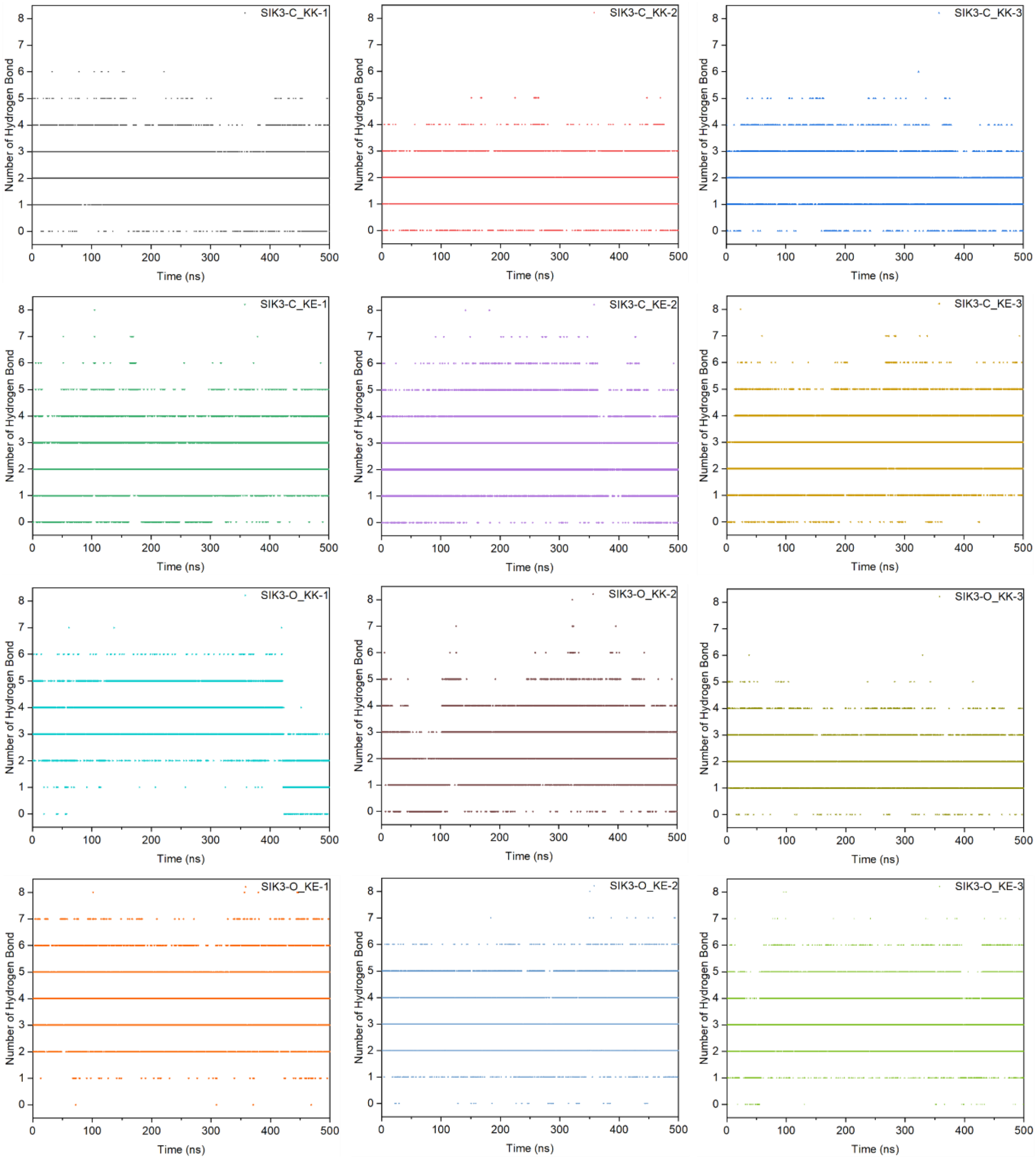

Figure S35. Hydrogen bond number along the 500-ns MD simulation for curcumin/SIK3.

Hydrogen bond is defined as the distance between the acceptor and donor atoms < 3.5 Å, with an internal angle between the H-acceptor and H-donor > 120º.

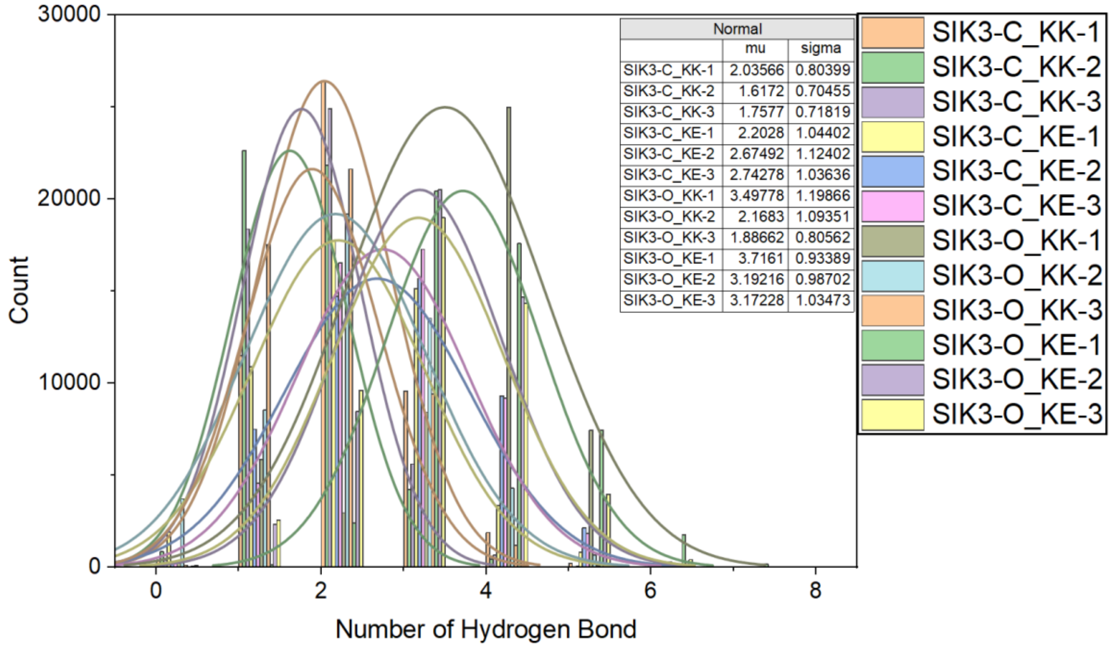

Figure S36. Statistical hydrogen bond number profile along the 500-ns MD simulation for curcumin/SIK3.
Hydrogen bond is defined as the distance between the acceptor and donor atoms < 3.5 Å, with an internal angle between the H-acceptor and H-donor > 120º.

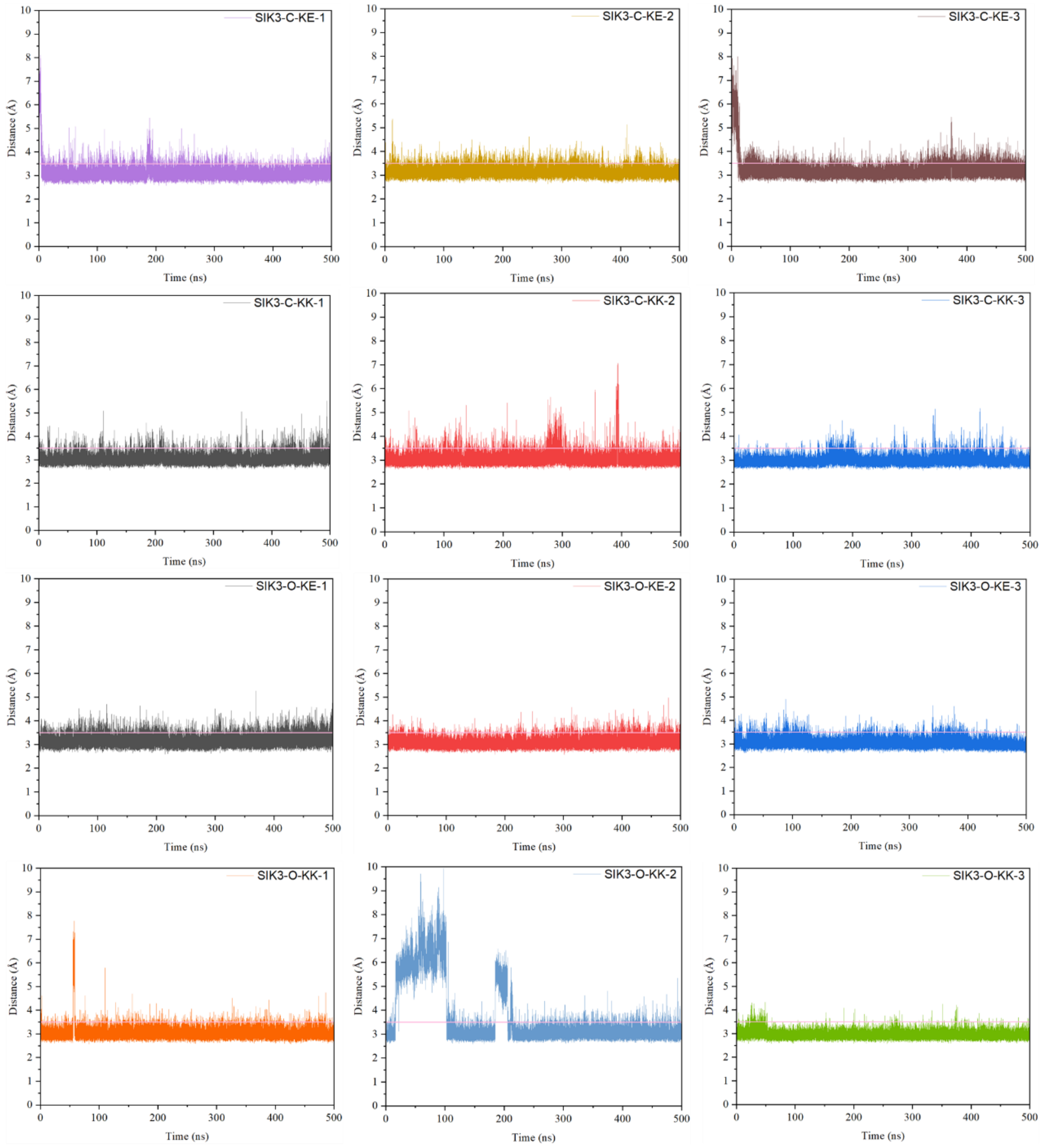

Figure S37. Distance between oxygen atom of diketo or ketoenol group of curcumin and nitrogen atom of A145 in hinge loop for curcumin/SIK3 systems.
The reference line with 3.5 Å applied in those figures from geometric criteria for hydrogen bond analysis.

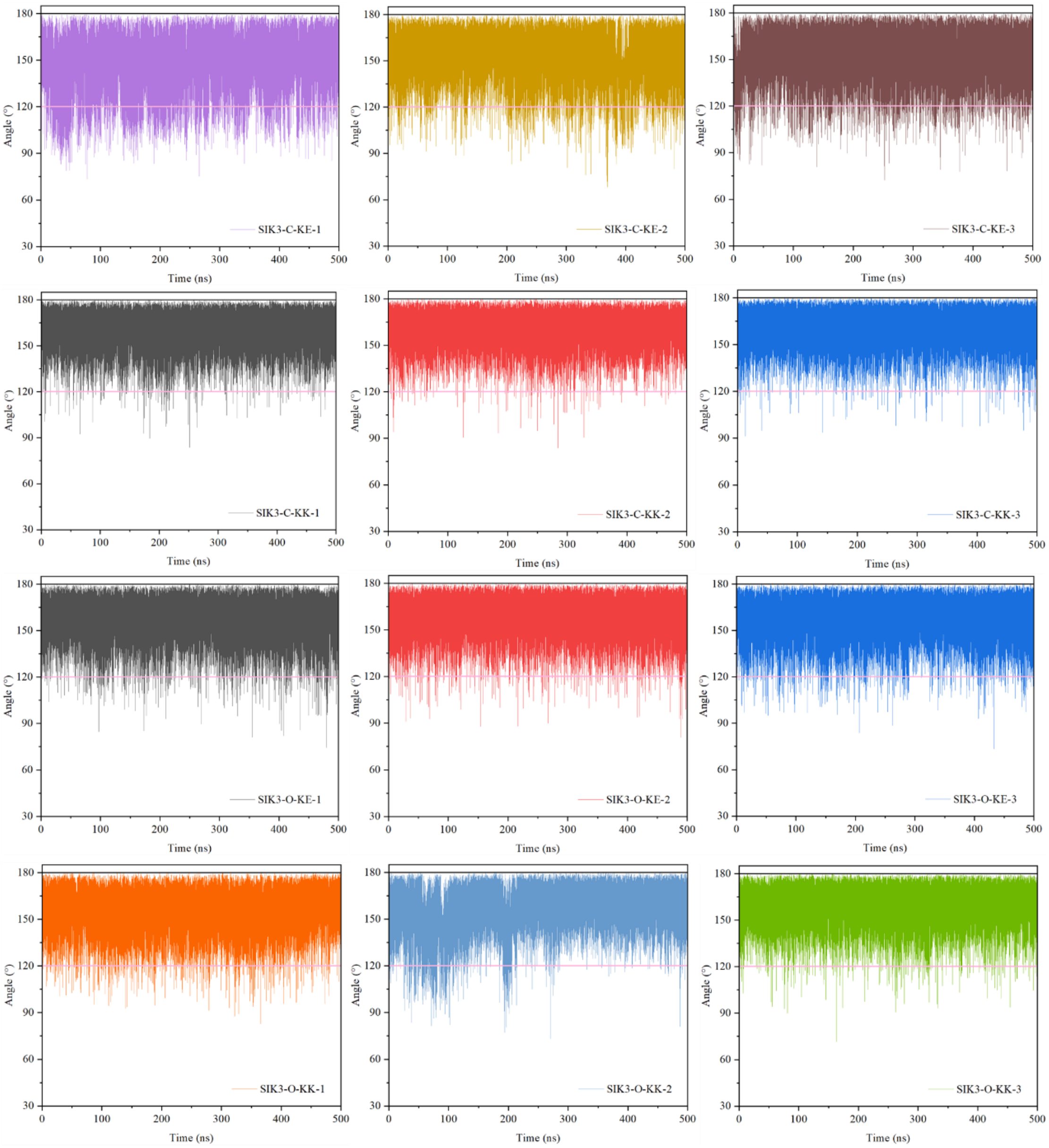

Figure S38. Angle among oxygen atom of diketo or ketoenol group of curcumin, hydrogen atom of A145, and nitrogen atom of A145 in hinge loop for curcumin/SIK3 systems.

The reference line with 120° applied in those figures from geometric criteria for hydrogen bond analysis.

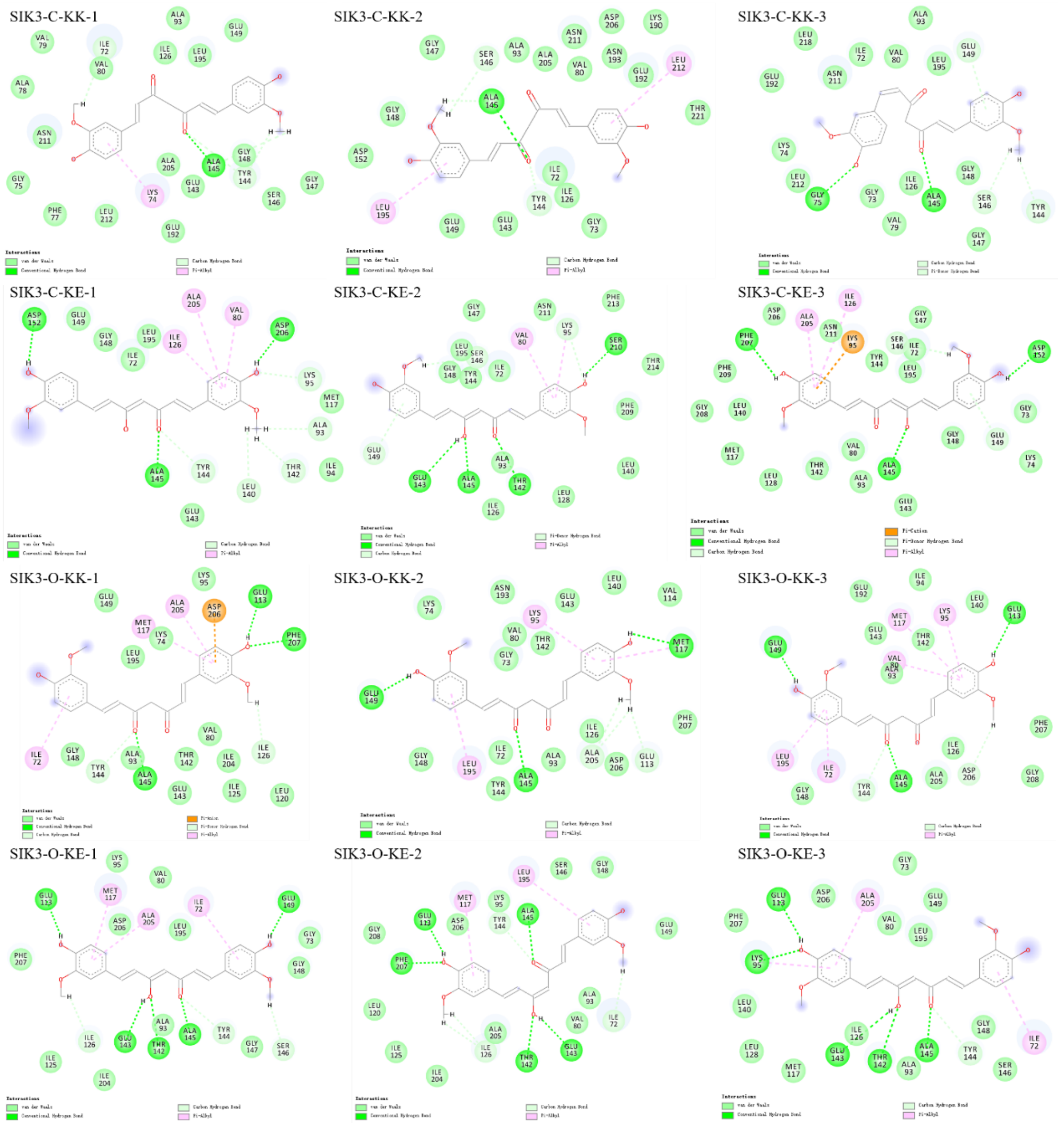

Figure S39. Two-dimension interaction between curcumin and human SIK2.

The cluster analysis was performed with 10000 frames from the last 200 ns simulation for every system. The presentative frame was extracted in the largest cluster based on the average linkage cluster algorithm and epsilon = 2.5 Å.

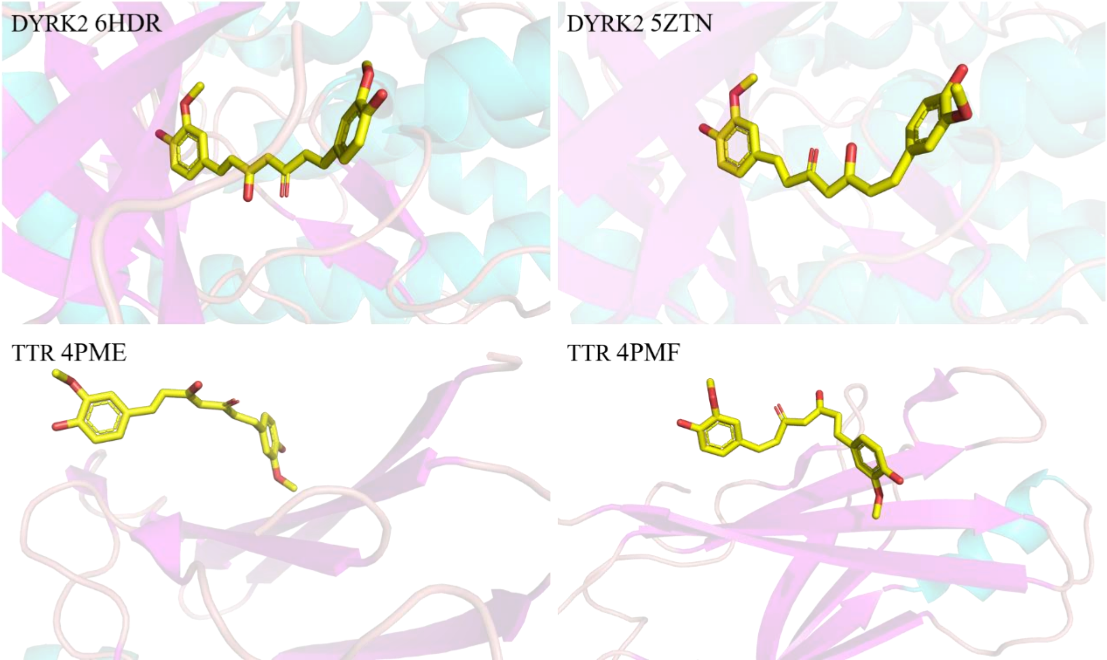

Figure S40. Crystal structure for curcumin binding with protein.

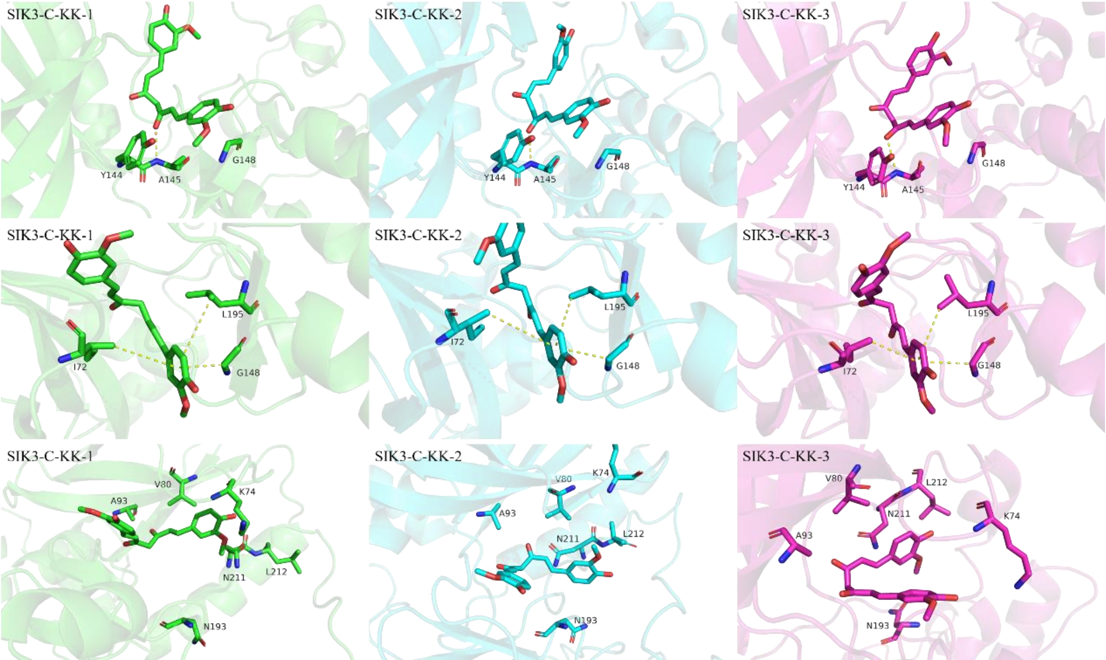

Figure S41. Interaction between curcumin and SIK3 for SIK3-C-KK systems.

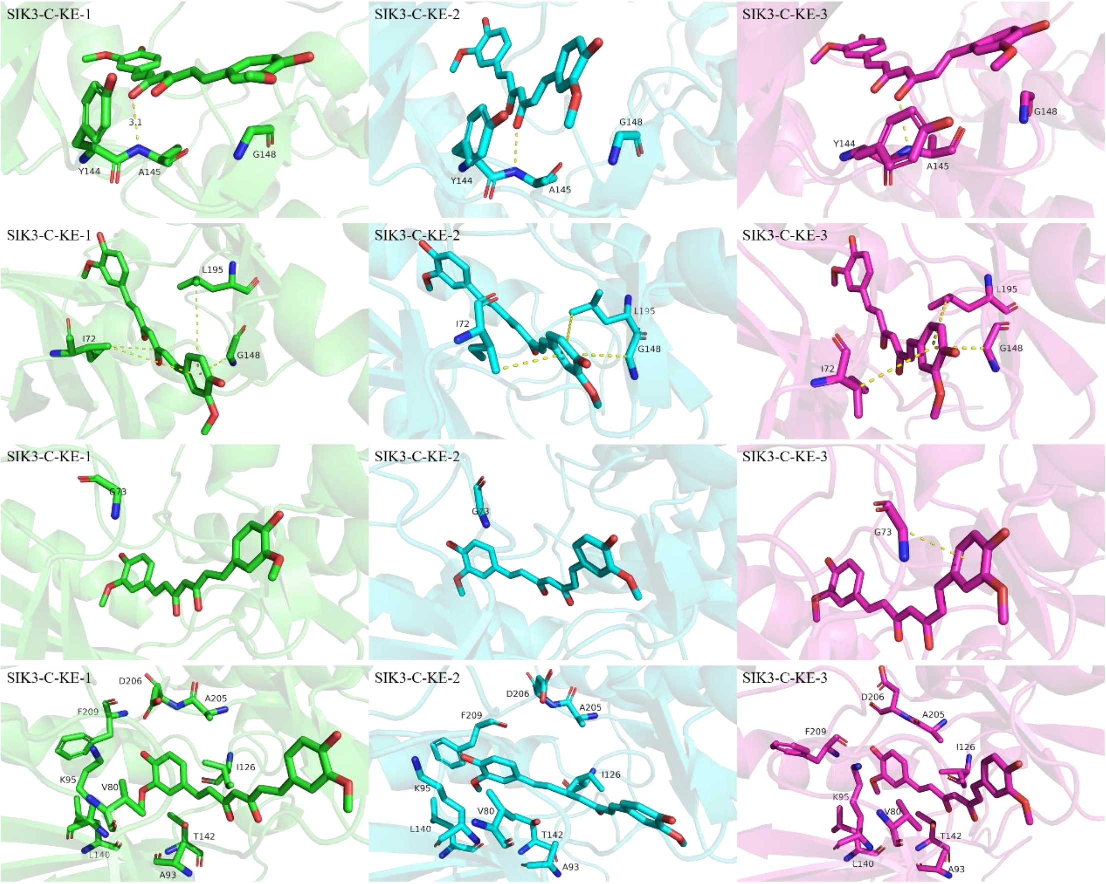

Figure S42. Interaction between curcumin and SIK3 for SIK3-C-KE systems.

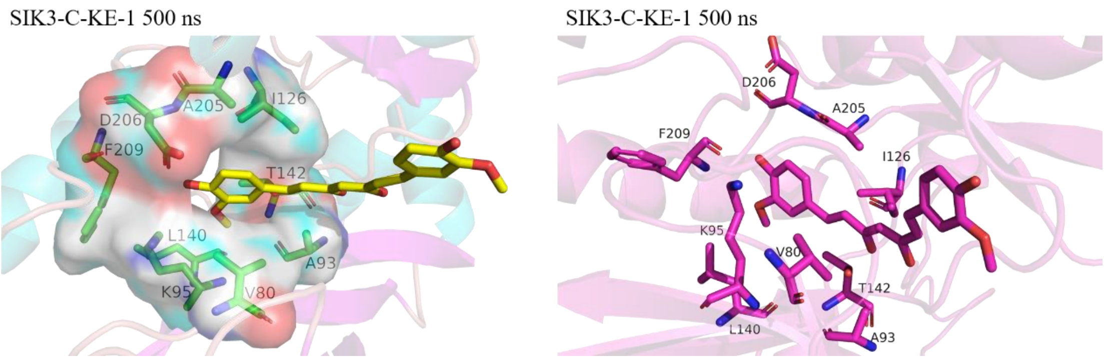

Figure S43. Interaction between curcumin and SIK3 for SIK3-C-KE-1 systems for back pocket.

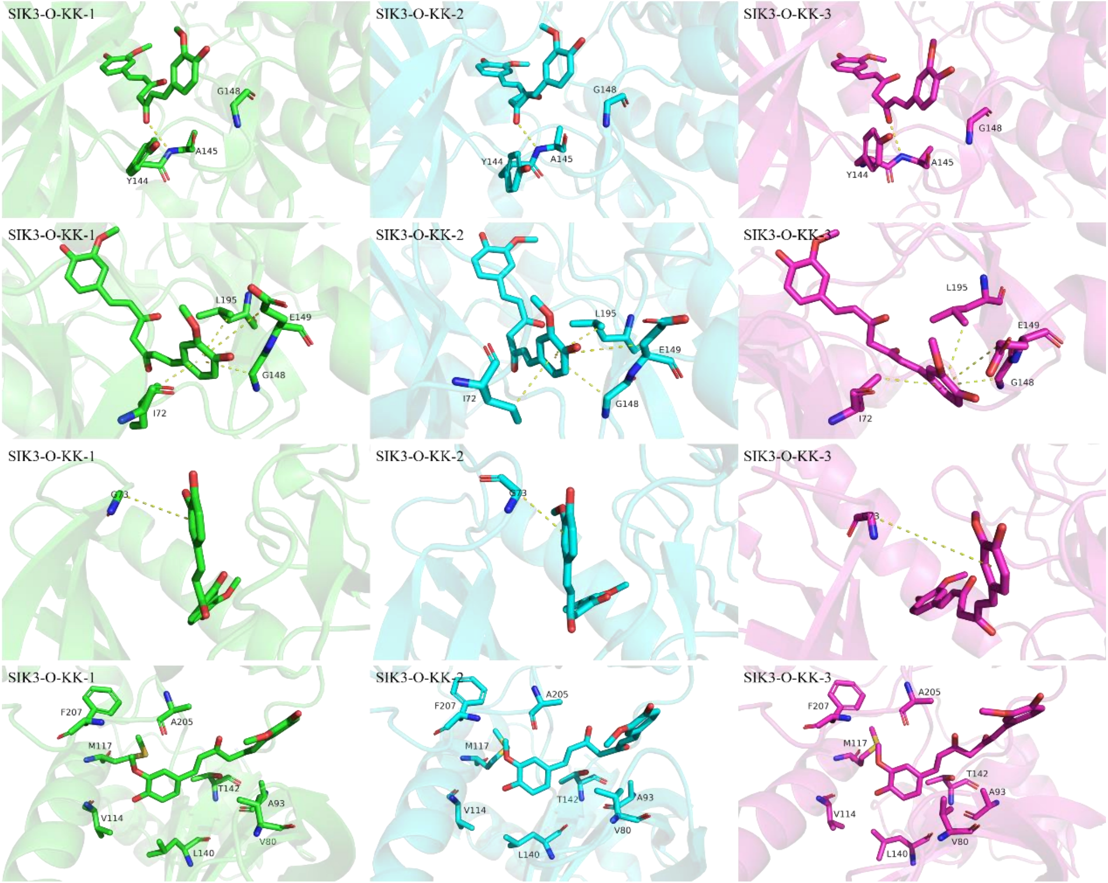

Figure S44. Interaction between curcumin and SIK3 for SIK3-C-KK systems.

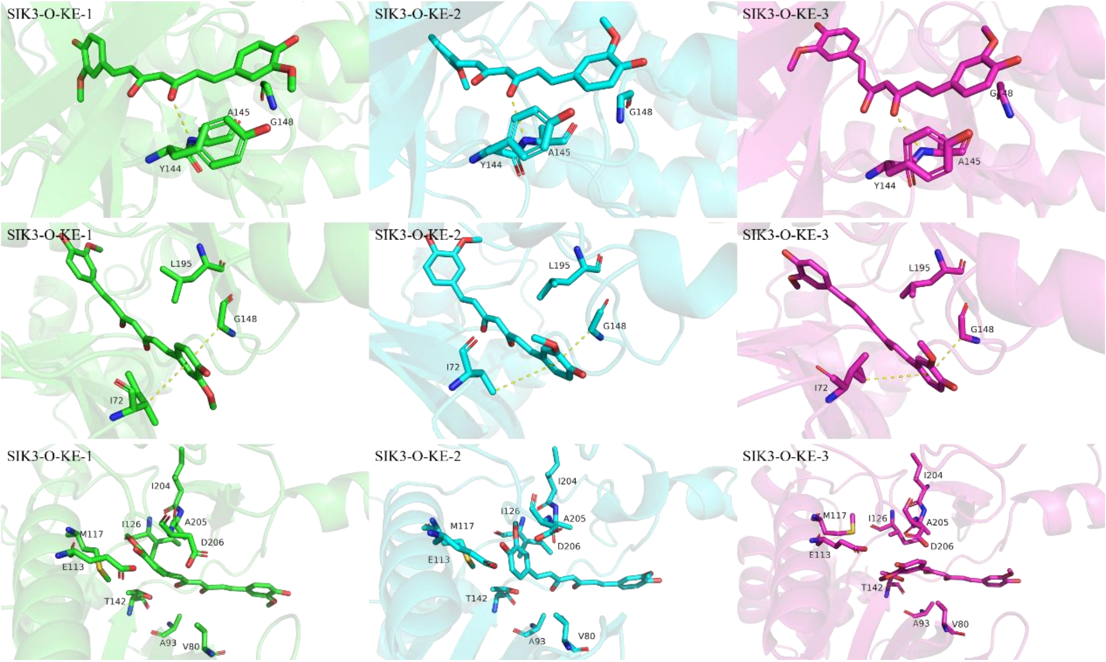

Figure S45. Interaction between curcumin and SIK3 for SIK3-C-KE systems.

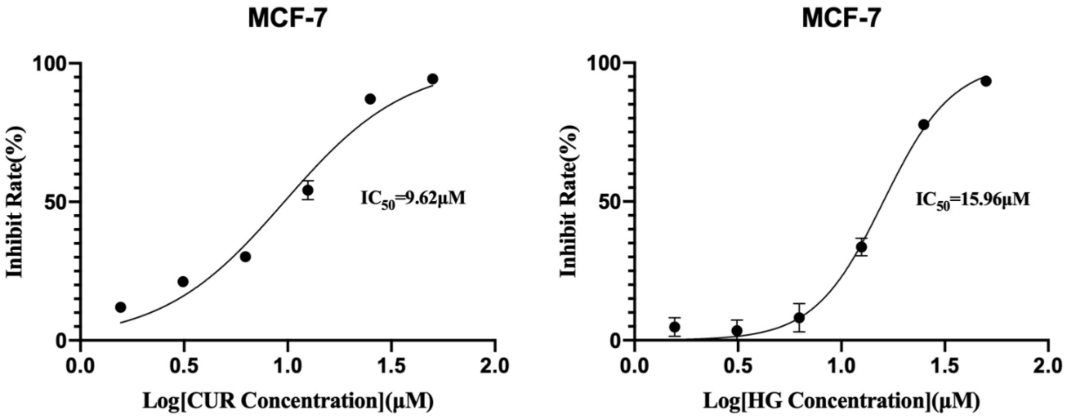

Figure S46. Cellular activities of curcumin and HG-9-91-01 in human breast cancer cell line MCF-7.

Three independent experiments were performed for all experiments. Data are plotted as mean ± SEM from two independent experiments.

Table S1. Root mean square deviation (RMSD) value of the SIK3, kinase domain, and curcumin for the curcumin/SIK3 complex systems although 500 ns MD simulations.

RMSD (Å)	SIK3	Kinase domain	Curcumin
SIK3-C_KK-1	2.64 ± 0.53	2.12 ± 0.51	3.64 ± 0.67
SIK3-C_KK-2	3.17 ± 0.39	2.36 ± 0.25	3.34 ± 0.53
SIK3-C_KK-3	2.96 ± 0.32	2.05 ± 0.31	3.17 ± 0.52
SIK3-C_KE-1	2.89 ± 0.28	2.55 ± 0.28	4.95 ± 0.74
SIK3-C_KE-2	2.86 ± 0.25	2.27 ± 0.22	3.91 ± 0.58
SIK3-C_KE-3	2.95 ± 0.19	2.43 ± 0.22	3.56 ± 0.37
SIK3-O_KK-1	2.45 ± 0.23	1.46 ± 0.18	1.98 ± 0.31
SIK3-O_KK-2	2.29 ± 0.31	1.73 ± 0.31	3.36 ± 0.88
SIK3-O_KK-3	2.62 ± 0.35	1.48 ± 0.15	2.50 ± 0.22
SIK3-O_KE-1	2.33 ± 0.29	1.57 ± 0.22	1.98 ± 0.25
SIK3-O_KE-2	2.22 ± 0.33	1.54 ± 0.19	1.21 ± 0.30
SIK3-O_KE-3	2.73 ± 0.39	1.63 ± 0.23	1.49 ± 0.42

Table S2. Statistical hydrogen bond number for curcumin/SIK3 systems although 500 ns MD simulations.

System	Hydrogen Bond Number
SIK3-C_KK-1	2.04 ± 0.80
SIK3-C_KK-2	1.62 ± 0.70
SIK3-C_KK-3	1.76 ± 0.72
SIK3-C_KE-1	2.20 ± 1.04
SIK3-C_KE-2	2.67 ± 1.12
SIK3-C_KE-3	2.74 ± 1.04
SIK3-O_KK-1	3.50 ± 1.20
SIK3-O_KK-2	2.17 ± 1.09
SIK3-O_KK-3	1.89 ± 0.81
SIK3-O_KE-1	3.72 ± 0.93
SIK3-O_KE-2	3.19 ± 0.99
SIK3-O_KE-3	3.17 ± 1.03

Hydrogen bond is defined as the distance between the acceptor and donor atoms < 3.5 Å, with an internal angle between the H-acceptor and H-donor > 120º.

Table S3. Statistical hydrogen bond distance between A145 and curcumin for curcumin/SIK3 systems although 500 ns MD simulations.

System	Hydrogen Bond Distance (Å)
SIK3-C_KK-1	3.03 ± 0.21
SIK3-C_KK-2	3.06 ± 0.30
SIK3-C_KK-3	2.98 ± 0.19
SIK3-C_KE-1	3.10 ± 0.36
SIK3-C_KE-2	3.15 ± 0.22
SIK3-C_KE-3	5.16 ± 0.35
SIK3-O_KK-1	3.03 ± 0.30
SIK3-O_KK-2	3.69 ± 1.29
SIK3-O_KK-3	2.95 ± 0.15
SIK3-O_KE-1	3.15 ± 0.23
SIK3-O_KE-2	3.07 ± 0.20
SIK3-O_KE-3	3.04 ± 0.19

Hydrogen bond is defined as the distance between the acceptor and donor atoms < 3.5 Å, with an internal angle between the H-acceptor and H-donor > 120º.

Table S4. Binding free energies ($\Delta\mathbf{G}_{\mathbf{bind}}^{\mathbf{cal}}$) for SIK3-C-KK-1 system.

Energy	Complex		Receptor		Ligand		Delta	
	Average	Std. Dev.	Average	Std. Dev.	Average	Std. Dev.	Average	Std. Dev.
E_{vdW}	-2616.58	27.28	-2576.12	27.86	-0.46	1.25	-40.00	4.30
E_{ele}	-23002.48	144.83	-22964.51	143.12	-16.25	5.98	-21.71	8.66
E_{polar}	-4097.30	119.29	-4108.75	117.71	-26.08	1.78	37.53	7.59
$E_{nonpolar}$	121.45	2.62	123.16	2.62	4.16	0.07	-5.87	0.58
E_{gas}	-5673.94	141.64	-5609.62	141.04	-2.61	5.59	-61.71	8.25
E_{solv}	-3975.85	118.05	-3985.60	116.31	-21.92	1.78	31.66	7.66
$E_{gas} + E_{sol}$	-9649.79	58.13	-9595.21	58.18	-24.53	4.98	-30.05	3.98
TS_{total}	3676.89	11.19	3644.35	11.01	54.39	0.41	-21.85	7.01
ΔG_{bind}^{cal}							-8.20	8.07

The binding free energies (ΔG_{bind}^{cal}) for curcumin/SIK3 complex and decomposition to electrostatic interaction (E_{ele}), van der Walls interaction (E_{vdW}), polar solvation free energies (E_{polar}), nonpolar solvation free energies ($E_{nonpolar}$), and entropy (TS_{total}). Energy values are presented in kcal/mol. Uncertainties were calculated as the root mean square error for all frames extracted from the trajectories.

Table S5. Binding free energies ($\Delta\mathbf{G}_{\mathbf{bind}}^{\mathbf{cal}}$) for SIK3-C-KK-2 system.

Energy	Complex		Receptor		Ligand		Delta	
	Average	Std. Dev.	Average	Std. Dev.	Average	Std. Dev.	Average	Std. Dev.
E_{vdW}	-2670.04	25.79	-2626.78	25.68	-0.87	1.23	-42.39	3.02
E_{ele}	-23091.00	114.64	-23046.49	114.79	-14.84	5.02	-29.66	12.22
E_{polar}	-4004.60	94.76	-4023.25	94.85	-26.47	1.53	45.12	9.86
$E_{nonpolar}$	114.23	2.20	116.48	2.17	4.11	0.10	-6.37	0.34
E_{gas}	-5826.67	113.87	-5753.17	113.66	-1.44	5.03	-72.06	11.64
E_{solv}	-3890.37	94.17	-3906.76	94.26	-22.36	1.53	38.75	9.77
$E_{gas} + E_{sol}$	-9717.04	57.57	-9659.93	57.47	-23.79	4.60	-33.31	3.36
TS_{total}	3661.82	10.54	3631.10	10.13	54.19	0.35	-23.47	5.15
ΔG_{bind}^{cal}							-9.84	6.15

The binding free energies (ΔG_{bind}^{cal}) for curcumin/SIK3 complex and decomposition to electrostatic interaction (E_{ele}), van der Walls interaction (E_{vdW}), polar solvation free energies (E_{polar}), nonpolar solvation free energies ($E_{nonpolar}$), and entropy (TS_{total}). Energy values are presented in kcal/mol. Uncertainties were calculated as the root mean square error for all frames extracted from the trajectories.

Table S6. Binding free energies (**ΔG_{bind}^{cal}**) for SIK3-C-KK-3 system.

Energy	Complex		Receptor		Ligand		Delta	
	Average	Std. Dev.	Average	Std. Dev.	Average	Std. Dev.	Average	Std. Dev.
E_{vdW}	-2629.59	25.85	-2587.15	25.73	-0.43	1.27	-42.01	2.69
E_{ele}	-23091.34	118.75	-23050.98	118.22	-16.70	5.86	-23.66	7.42
E_{polar}	-4008.87	97.35	-4022.26	96.88	-25.77	1.76	39.16	6.36
$E_{nonpolar}$	119.32	2.25	121.40	2.17	4.13	0.08	-6.22	0.37
E_{gas}	-5776.55	118.07	-5708.12	117.57	-2.76	5.28	-65.67	7.48
E_{solv}	-3889.55	96.50	-3900.86	96.07	-21.63	1.74	32.95	6.23
$E_{gas} + E_{sol}$	-9666.10	56.66	-9608.98	56.57	-24.40	4.80	-32.73	3.11
TS_{total}	3673.13	11.00	3641.40	10.88	54.36	0.42	-22.64	6.88
ΔG_{bind}^{cal}							-10.09	7.55

The binding free energies (ΔG_{bind}^{cal}) for curcumin/SIK3 complex and decomposition to electrostatic interaction (E_{ele}), van der Walls interaction (E_{vdW}), polar solvation free energies (E_{polar}), nonpolar solvation free energies ($E_{nonpolar}$), and entropy (TS_{total}). Energy values are presented in kcal/mol. Uncertainties were calculated as the root mean square error for all frames extracted from the trajectories.

Table S7. Binding free energies ($\Delta\mathbf{G}_{\mathbf{bind}}^{\mathbf{cal}}$) for SIK3-C-KE-1 system.

Energy	Complex		Receptor		Ligand		Delta	
	Average	Std. Dev.	Average	Std. Dev.	Average	Std. Dev.	Average	Std. Dev.
E_{vdW}	-2638.97	25.64	-2603.34	25.31	1.26	1.16	-36.89	2.96
E_{ele}	-22933.97	139.22	-22884.37	138.82	-6.41	5.33	-43.19	11.62
E_{polar}	-4157.82	125.62	-4184.02	125.05	-22.48	1.84	48.69	8.61
$E_{nonpolar}$	117.41	2.31	119.31	2.30	4.25	0.02	-6.14	0.35
E_{gas}	-5657.88	145.29	-5553.97	144.31	-23.83	5.79	-80.08	11.30
E_{solv}	-4040.41	124.95	-4064.71	124.40	-18.24	1.84	42.54	8.36
$E_{gas} + E_{sol}$	-9698.28	55.42	-9618.68	55.11	-42.07	5.06	-37.53	3.91
TS_{total}	3669.51	10.35	3636.76	10.38	54.58	0.67	-21.83	6.49
ΔG_{bind}^{cal}							-15.70	7.57

The binding free energies (ΔG_{bind}^{cal}) for curcumin/SIK3 complex and decomposition to electrostatic interaction (E_{ele}), van der Walls interaction (E_{vdW}), polar solvation free energies (E_{polar}), nonpolar solvation free energies ($E_{nonpolar}$), and entropy (TS_{total}). Energy values are presented in kcal/mol. Uncertainties were calculated as the root mean square error for all frames extracted from the trajectories.

Table S8. Binding free energies ($\Delta\mathbf{G}_{\mathbf{bind}}^{\mathbf{cal}}$) for SIK3-C-KE-2 system.

Energy	Complex		Receptor		Ligand		Delta	
	Average	Std. Dev.	Average	Std. Dev.	Average	Std. Dev.	Average	Std. Dev.
E_{vdW}	-2674.70	24.70	-2634.62	24.03	1.93	1.41	-42.01	4.16
E_{ele}	-23090.79	149.82	-23055.77	153.24	-11.63	6.25	-23.39	12.15
E_{polar}	-4023.10	141.82	-4037.57	144.76	-20.75	2.42	35.22	8.24
$E_{nonpolar}$	113.86	2.20	116.15	2.14	4.24	0.02	-6.54	0.44
E_{gas}	-5803.73	154.92	-5712.13	158.39	-26.20	5.59	-65.40	11.52
E_{solv}	-3909.25	141.26	-3921.41	144.17	-16.52	2.42	28.68	8.18
$E_{gas} + E_{sol}$	-9712.98	57.67	-9633.54	57.26	-42.72	4.74	-36.72	5.37
TS_{total}	3661.85	9.55	3629.77	10.39	53.97	1.27	-21.89	5.77
ΔG_{bind}^{cal}							-14.83	7.88

The binding free energies (ΔG_{bind}^{cal}) for curcumin/SIK3 complex and decomposition to electrostatic interaction (E_{ele}), van der Walls interaction (E_{vdW}), polar solvation free energies (E_{polar}), nonpolar solvation free energies ($E_{nonpolar}$), and entropy (TS_{total}). Energy values are presented in kcal/mol. Uncertainties were calculated as the root mean square error for all frames extracted from the trajectories.

Table S9. Binding free energies ($\Delta\mathbf{G}_{\mathbf{bind}}^{\mathbf{cal}}$) for SIK3-C-KE-3 system.

Energy	Complex		Receptor		Ligand		Delta	
	Average	Std. Dev.	Average	Std. Dev.	Average	Std. Dev.	Average	Std. Dev.
E_{vdW}	-2641.18	24.92	-2595.63	24.40	2.33	1.37	-47.88	2.86
E_{ele}	-23059.19	133.74	-23020.27	133.01	-12.60	4.94	-26.33	10.35
E_{polar}	-4082.75	108.45	-4100.29	107.86	-19.95	1.23	37.48	7.93
$E_{nonpolar}$	116.48	2.41	119.62	2.35	4.24	0.02	-7.38	0.25
E_{gas}	-5727.32	133.48	-5625.73	132.29	-27.39	5.09	-74.20	10.43
E_{solv}	-3966.28	107.33	-3980.67	106.76	-15.71	1.23	30.10	7.81
$E_{gas} + E_{sol}$	-9693.59	57.76	-9606.39	57.01	-43.10	4.70	-44.10	3.92
TS_{total}	3678.66	9.74	3644.17	9.09	53.71	1.37	-19.22	6.69
ΔG_{bind}^{cal}							-24.88	7.75

The binding free energies (ΔG_{bind}^{cal}) for curcumin/SIK3 complex and decomposition to electrostatic interaction (E_{ele}), van der Walls interaction (E_{vdW}), polar solvation free energies (E_{polar}), nonpolar solvation free energies ($E_{nonpolar}$), and entropy (TS_{total}). Energy values are presented in kcal/mol. Uncertainties were calculated as the root mean square error for all frames extracted from the trajectories.

Table S10. Binding free energies ($\Delta \mathbf{G}_{\mathbf{bind}}^{\mathbf{cal}}$) for SIK3-O-KK-1 system.

Energy	Complex		Receptor		Ligand		Delta	
	Average	Std. Dev.	Average	Std. Dev.	Average	Std. Dev.	Average	Std. Dev.
E_{vdW}	-2657.81	23.98	-2612.56	23.53	-0.12	1.15	-45.12	3.45
E_{ele}	-23227.07	138.92	-23173.91	137.84	-17.30	5.62	-35.86	14.92
E_{polar}	-3900.97	113.90	-3923.63	112.09	-24.99	1.84	47.66	10.33
$E_{nonpolar}$	115.13	2.22	117.87	2.13	4.20	0.03	-6.94	0.34
E_{gas}	-5963.13	135.20	-5878.42	132.57	-3.73	5.58	-80.98	13.58
E_{solv}	-3785.84	113.04	-3805.76	111.24	-20.80	1.83	40.72	10.48
$E_{gas} + E_{sol}$	-9748.97	56.27	-9684.17	55.67	-24.53	5.04	-40.26	4.49
TS_{total}	3660.34	10.68	3628.54	10.18	54.68	0.80	-22.88	5.48
ΔG_{bind}^{cal}							-17.38	7.08

The binding free energies (ΔG_{bind}^{cal}) for curcumin/SIK3 complex and decomposition to electrostatic interaction (E_{ele}), van der Walls interaction (E_{vdW}), polar solvation free energies (E_{polar}), nonpolar solvation free energies ($E_{nonpolar}$), and entropy (TS_{total}). Energy values are presented in kcal/mol. Uncertainties were calculated as the root mean square error for all frames extracted from the trajectories.

Table S11. Binding free energies ($\Delta\mathbf{G}_{\mathbf{bind}}^{\mathbf{cal}}$) for SIK3-O-KK-2 system.

Energy	Complex		Receptor		Ligand		Delta	
	Average	Std. Dev.	Average	Std. Dev.	Average	Std. Dev.	Average	Std. Dev.
E_{vdW}	-2662.06	25.05	-2613.96	24.55	-0.33	1.13	-47.77	3.38
E_{ele}	-23148.58	134.79	-23099.38	133.73	-17.27	4.72	-31.93	8.96
E_{polar}	-4031.53	111.82	-4052.47	110.62	-25.03	1.52	45.97	6.84
$E_{nonpolar}$	116.33	2.16	119.67	2.08	4.19	0.03	-7.53	0.32
E_{gas}	-5782.45	131.66	-5699.15	130.04	-3.60	5.01	-79.70	9.16
E_{solv}	-3915.20	110.88	-3932.80	109.79	-20.85	1.51	38.44	6.70
$E_{gas} + E_{sol}$	-9697.66	55.10	-9631.96	55.08	-24.45	4.87	-41.25	3.91
TS_{total}	3669.13	11.34	3636.65	10.66	54.75	0.63	-22.27	6.68
ΔG_{bind}^{cal}							-18.98	7.74

The binding free energies (ΔG_{bind}^{cal}) for curcumin/SIK3 complex and decomposition to electrostatic interaction (E_{ele}), van der Walls interaction (E_{vdW}), polar solvation free energies (E_{polar}), nonpolar solvation free energies ($E_{nonpolar}$), and entropy (TS_{total}). Energy values are presented in kcal/mol. Uncertainties were calculated as the root mean square error for all frames extracted from the trajectories.

Table S12. Binding free energies ($\Delta\mathbf{G}_{\mathbf{bind}}^{\mathbf{cal}}$) for SIK3-O-KK-3 system.

Energy	Complex		Receptor		Ligand		Delta	
	Average	Std. Dev.	Average	Std. Dev.	Average	Std. Dev.	Average	Std. Dev.
E_{vdW}	-2644.35	25.43	-2596.72	25.16	-0.25	1.20	-47.39	2.90
E_{ele}	-23058.21	119.79	-23012.12	119.29	-24.69	2.99	-21.40	6.53
E_{polar}	-4038.31	101.46	-4052.08	101.11	-22.29	0.92	36.05	4.76
$E_{nonpolar}$	115.94	2.39	119.04	2.41	4.18	0.03	-7.28	0.26
E_{gas}	-5773.91	122.15	-5698.53	121.71	-6.59	4.78	-68.79	6.25
E_{solv}	-3922.37	100.41	-3933.04	100.07	-18.10	0.91	28.77	4.69
$E_{gas} + E_{sol}$	-9696.28	56.00	-9631.57	55.75	-24.69	4.70	-40.02	3.40
TS_{total}	3667.31	9.76	3633.09	9.94	54.65	0.48	-20.43	6.70
ΔG_{bind}^{cal}							-19.59	7.51

The binding free energies (ΔG_{bind}^{cal}) for curcumin/SIK3 complex and decomposition to electrostatic interaction (E_{ele}), van der Walls interaction (E_{vdW}), polar solvation free energies (E_{polar}), nonpolar solvation free energies ($E_{nonpolar}$), and entropy (TS_{total}). Energy values are presented in kcal/mol. Uncertainties were calculated as the root mean square error for all frames extracted from the trajectories.

Table S13. Binding free energies ($\Delta\mathbf{G}_{\mathbf{bind}}^{\mathbf{cal}}$) for SIK3-O-KE-1 system.

Energy	Complex		Receptor		Ligand		Delta	
	Average	Std. Dev.	Average	Std. Dev.	Average	Std. Dev.	Average	Std. Dev.
E_{vdW}	-2656.60	24.33	-2611.65	24.42	0.82	1.12	-45.77	3.54
E_{ele}	-23149.91	141.77	-23106.13	142.51	-7.03	3.71	-36.75	9.97
E_{polar}	-3971.62	115.58	-3992.46	117.05	-21.70	1.26	42.53	7.45
$E_{nonpolar}$	117.16	2.48	119.98	2.47	4.25	0.02	-7.07	0.29
E_{gas}	-5853.38	132.79	-5747.60	133.89	-23.25	5.37	-82.52	9.38
E_{solv}	-3854.46	114.37	-3872.48	115.86	-17.44	1.26	35.46	7.41
$E_{gas} + E_{sol}$	-9707.84	54.69	-9620.08	54.47	-40.70	5.03	-47.06	4.42
TS_{total}	3664.27	11.72	3631.22	11.23	54.24	1.13	-21.20	7.88
ΔG_{bind}^{cal}							-25.86	9.04

The binding free energies (ΔG_{bind}^{cal}) for curcumin/SIK3 complex and decomposition to electrostatic interaction (E_{ele}), van der Walls interaction (E_{vdW}), polar solvation free energies (E_{polar}), nonpolar solvation free energies ($E_{nonpolar}$), and entropy (TS_{total}). Energy values are presented in kcal/mol. Uncertainties were calculated as the root mean square error for all frames extracted from the trajectories.

Table S14. Binding free energies ($\Delta \mathbf{G}_{\mathbf{bind}}^{\mathbf{cal}}$) for SIK3-O-KE-2 system.

Energy	Complex		Receptor		Ligand		Delta	
	Average	Std. Dev.	Average	Std. Dev.	Average	Std. Dev.	Average	Std. Dev.
E_{vdW}	-2645.14	29.03	-2600.88	28.52	1.13	1.16	-45.38	3.46
E_{ele}	-22986.01	132.96	-22945.28	133.59	-10.40	2.88	-30.33	6.96
E_{polar}	-4138.31	115.07	-4157.61	115.64	-20.47	0.86	39.77	4.77
$E_{nonpolar}$	117.71	2.83	120.40	2.85	4.25	0.02	-6.94	0.30
E_{gas}	-5683.45	136.11	-5582.11	136.70	-25.63	4.94	-75.71	6.63
E_{solv}	-4020.60	113.66	-4037.20	114.26	-16.22	0.85	32.83	4.76
$E_{gas} + E_{sol}$	-9704.05	57.49	-9619.32	57.30	-41.85	4.82	-42.89	4.17
TS_{total}	3672.91	8.83	3639.99	10.36	54.34	0.55	-21.42	7.52
ΔG_{bind}^{cal}							-21.46	8.60

The binding free energies (ΔG_{bind}^{cal}) for curcumin/SIK3 complex and decomposition to electrostatic interaction (E_{ele}), van der Walls interaction (E_{vdW}), polar solvation free energies (E_{polar}), nonpolar solvation free energies ($E_{nonpolar}$), and entropy (TS_{total}). Energy values are presented in kcal/mol. Uncertainties were calculated as the root mean square error for all frames extracted from the trajectories.

Table S15. Binding free energies ($\Delta \mathbf{G}_{\mathbf{bind}}^{\mathbf{cal}}$) for SIK3-O-KE-3 system.

Energy	Complex		Receptor		Ligand		Delta	
	Average	Std. Dev.	Average	Std. Dev.	Average	Std. Dev.	Average	Std. Dev.
E_{vdW}	-2649.90	25.53	-2605.96	25.16	1.30	1.26	-45.24	3.23
E_{ele}	-23026.56	197.42	-22982.62	194.26	-14.33	4.83	-29.61	7.86
E_{polar}	-4088.82	145.56	-4109.89	144.29	-19.80	1.19	40.88	5.78
$E_{nonpolar}$	116.89	2.46	119.67	2.43	4.24	0.02	-7.02	0.35
E_{gas}	-5745.36	177.96	-5643.62	177.30	-26.90	5.41	-74.85	7.74
E_{solv}	-3971.92	144.29	-3990.22	143.09	-15.55	1.18	33.85	5.79
$E_{gas} + E_{sol}$	-9717.29	62.12	-9633.84	62.33	-42.45	5.21	-40.99	4.28
TS_{total}	3674.54	10.88	3640.25	11.35	54.36	0.40	-20.07	6.79
ΔG_{bind}^{cal}							-20.92	8.03

The binding free energies (ΔG_{bind}^{cal}) for curcumin/SIK3 complex and decomposition to electrostatic interaction (E_{ele}), van der Walls interaction (E_{vdW}), polar solvation free energies (E_{polar}), nonpolar solvation free energies ($E_{nonpolar}$), and entropy (TS_{total}). Energy values are presented in kcal/mol. Uncertainties were calculated as the root mean square error for all frames extracted from the trajectories.

Table S16. Free energy decomposition for the curcumin/SIK3 (**SIK3-C-KK-1**) complex on the individual residue basis, where decomposition is performed in terms of the contributions from van der Waals energy, the electrostatic interaction energy, the nonpolar solvation free energy, the polar solvation free energy, the backbone energy, and the side chain energy

Residues	ΔE_{vdW}		ΔE_{ele}		ΔG_{polar}		$\Delta G_{nonpolar}$		$\Delta G_{subtotal}$		$S\Delta G_{subtotal}$		$B\Delta G_{subtotal}$	
	Avg.	Std. Dev.	Avg.	Std. Dev.	Avg.	Std. Dev.	Avg.	Std. Dev.	Avg.	Std. Dev.	Avg.	Std. Dev.	Avg.	Std. Dev.
I72	-2.24	0.60	-0.62	0.37	1.19	0.45	-0.33	0.09	-2.00	0.63	-2.06	0.59	0.06	0.23
K74	-1.19	0.82	0.46	2.01	0.17	1.64	-0.19	0.10	-0.75	0.71	-0.70	0.57	-0.04	0.52
V80	-1.19	0.34	-0.36	0.15	0.53	0.16	-0.12	0.04	-1.15	0.34	-0.94	0.31	-0.21	0.11
A93	-0.41	0.16	-0.41	0.17	0.37	0.14	-0.05	0.03	-0.49	0.17	-0.38	0.16	-0.11	0.06
L126	-0.42	0.20	-0.21	0.06	0.27	0.06	-0.05	0.03	-0.42	0.22	-0.46	0.21	0.04	0.01
Y144	-2.24	0.53	-2.11	0.55	2.15	0.50	-0.26	0.07	-2.45	0.51	-1.56	0.47	-0.89	0.21
A145	-1.01	0.46	-2.53	0.58	2.27	0.36	-0.07	0.03	-1.35	0.40	-0.58	0.12	-0.78	0.39
G148	-0.95	0.33	-0.02	0.26	0.16	0.22	-0.16	0.03	-0.97	0.38	-0.37	0.12	-0.60	0.29
N193	-0.07	0.04	0.06	0.09	-0.02	0.11	-0.00	0.01	-0.03	0.04	-0.02	0.03	-0.01	0.02
L195	-1.25	0.31	-0.09	0.09	0.13	0.08	-0.25	0.04	-1.45	0.32	-1.41	0.31	-0.04	0.03
N211	-1.86	0.66	-0.06	0.71	1.08	0.72	-0.29	0.07	-1.12	0.63	-1.07	0.56	-0.05	0.21
L212	-0.48	0.33	0.03	0.08	0.07	0.08	-0.07	0.05	-0.44	0.33	-0.42	0.30	-0.03	0.08

Energies are in kcal/mol.

Table S17. Free energy decomposition for the curcumin/SIK3 (**SIK3-C-KK-2**) complex on the individual residue basis, where decomposition is performed in terms of the contributions from van der Waals energy, the electrostatic interaction energy, the nonpolar solvation free energy, the polar solvation free energy, the backbone energy, and the side chain energy

Residues	ΔE_{vdW}		ΔE_{ele}		ΔG_{polar}		$\Delta G_{nonpolar}$		$\Delta G_{subtotal}$		$S\Delta G_{subtotal}$		$B\Delta G_{subtotal}$	
	Avg.	Std. Dev.	Avg.	Std. Dev.	Avg.	Std. Dev.	Avg.	Std. Dev.	Avg.	Std. Dev.	Avg.	Std. Dev.	Avg.	Std. Dev.
I72	-2.41	0.52	-0.76	0.47	1.37	0.61	-0.42	0.07	-2.22	0.62	-2.31	0.48	0.10	0.31
K74	-0.12	0.09	-0.05	0.30	0.14	0.31	-0.00	0.01	-0.04	0.08	-0.08	0.06	0.04	0.04
V80	-0.78	0.26	-0.44	0.16	0.38	0.11	-0.04	0.02	-0.89	0.25	-0.68	0.22	-0.21	0.08
A93	-0.44	0.16	-0.55	0.17	0.45	0.12	-0.05	0.01	-0.59	0.19	-0.42	0.17	-0.18	0.08
L126	-0.68	0.19	-0.18	0.07	0.26	0.06	-0.06	0.01	-0.66	0.19	-0.70	0.18	0.05	0.02
Y144	-1.84	0.48	-2.16	0.50	1.97	0.40	-0.21	0.05	-2.23	0.47	-1.39	0.40	-0.85	0.25
A145	-1.17	0.44	-2.40	0.64	2.31	0.36	-0.08	0.02	-1.35	0.39	-0.65	0.14	-0.69	0.37
G148	-1.01	0.30	0.11	0.31	0.00	0.30	-0.15	0.02	-1.04	0.36	-0.38	0.10	-0.67	0.29
N193	-0.63	0.26	0.45	0.31	-0.35	0.26	-0.01	0.01	-0.54	0.26	-0.41	0.24	-0.13	0.07
L195	-1.60	0.29	-0.00	0.10	0.05	0.10	-0.22	0.03	-1.77	0.30	-1.72	0.29	-0.05	0.03
N211	-1.96	0.38	1.16	0.46	-0.30	0.48	-0.21	0.04	-1.31	0.49	-1.16	0.42	-0.15	0.12
L212	-0.93	0.31	-0.02	0.10	0.18	0.10	-0.17	0.05	-0.95	0.36	-0.90	0.32	-0.05	0.10

Energies are in kcal/mol.

Table S18. Free energy decomposition for the curcumin/SIK3 (**SIK3-C-KK-3**) complex on the individual residue basis, where decomposition is performed in terms of the contributions from van der Waals energy, the electrostatic interaction energy, the nonpolar solvation free energy, the polar solvation free energy, the backbone energy, and the side chain energy

Residues	ΔE_{vdW}		ΔE_{ele}		ΔG_{polar}		$\Delta G_{nonpolar}$		$\Delta G_{subtotal}$		$S\Delta G_{subtotal}$		$B\Delta G_{subtotal}$	
	Avg.	Std. Dev.	Avg.	Std. Dev.	Avg.	Std. Dev.	Avg.	Std. Dev.	Avg.	Std. Dev.	Avg.	Std. Dev.	Avg.	Std. Dev.
I72	-2.17	0.65	-0.38	0.31	0.86	0.50	-0.30	0.09	-2.00	0.64	-2.01	0.60	0.01	0.18
K74	-1.40	0.75	1.77	2.01	-1.12	1.67	-0.19	0.11	-0.94	0.76	-0.85	0.62	-0.09	0.37
V80	-0.91	0.34	-0.39	0.15	0.43	0.14	-0.08	0.04	-0.95	0.34	-0.85	0.32	-0.11	0.08
A93	-0.41	0.19	-0.48	0.18	0.42	0.14	-0.05	0.02	-0.53	0.20	-0.39	0.18	-0.14	0.07
L126	-0.47	0.21	-0.20	0.07	0.25	0.07	-0.06	0.02	-0.48	0.22	-0.52	0.22	0.04	0.02
Y144	-2.13	0.56	-2.09	0.57	1.99	0.51	-0.22	0.08	-2.45	0.52	-1.55	0.50	-0.90	0.21
A145	-0.91	0.49	-2.57	0.53	2.19	0.32	-0.06	0.02	-1.35	0.39	-0.56	0.13	-0.79	0.38
G148	-1.06	0.24	-0.08	0.24	0.22	0.20	-0.17	0.02	-1.09	0.29	-0.42	0.08	-0.68	0.23
N193	-0.20	0.20	-0.11	0.21	0.23	0.25	-0.01	0.03	-0.09	0.14	-0.06	0.12	-0.03	0.06
L195	-1.42	0.37	-0.08	0.11	0.15	0.08	-0.25	0.04	-1.60	0.37	-1.55	0.36	-0.05	0.03
N211	-1.94	0.48	0.49	0.78	0.71	0.81	-0.29	0.06	-1.03	0.46	-0.97	0.41	-0.06	0.17
L212	-0.89	0.40	0.03	0.21	0.07	0.18	-0.14	0.06	-0.93	0.44	-0.77	0.33	-0.16	0.21

Energies are in kcal/mol.

Table S19. Free energy decomposition for the curcumin/SIK3 (**SIK3-C-KE-1**) complex on the individual residue basis, where decomposition is performed in terms of the contributions from van der Waals energy, the electrostatic interaction energy, the nonpolar solvation free energy, the polar solvation free energy, the backbone energy, and the side chain energy

Residues	ΔE_{vdW}		ΔE_{ele}		ΔG_{polar}		$\Delta G_{nonpolar}$		$\Delta G_{subtotal}$		$S\Delta G_{subtotal}$		$B\Delta G_{subtotal}$	
	Avg.	Std. Dev.	Avg.	Std. Dev.	Avg.	Std. Dev.	Avg.	Std. Dev.	Avg.	Std. Dev.	Avg.	Std. Dev.	Avg.	Std. Dev.
I72	-1.46	0.28	-0.03	0.11	0.11	0.12	-0.25	0.04	-1.64	0.30	-1.59	0.29	-0.04	0.04
G73	-0.11	0.06	0.18	0.08	-0.07	0.07	-0.01	0.03	-0.01	0.06	-0.03	0.02	0.02	0.04
V80	-1.51	0.23	-0.04	0.08	0.05	0.05	-0.17	0.03	-1.67	0.24	-1.59	0.23	-0.09	0.05
A93	-1.03	0.23	-0.24	0.22	0.55	0.19	-0.06	0.02	-0.77	0.25	-0.68	0.22	-0.09	0.12
K95	-0.95	0.32	-1.17	1.09	1.81	1.04	-0.08	0.02	-0.39	0.56	-0.38	0.49	-0.02	0.24
L126	-1.10	0.22	-0.06	0.05	0.03	0.07	-0.11	0.02	-1.24	0.24	-1.15	0.23	-0.09	0.05
L140	-0.34	0.14	-0.55	0.17	0.29	0.11	-0.01	0.01	-0.60	0.16	-0.17	0.06	-0.43	0.14
T142	-0.67	0.44	-0.73	0.19	0.75	0.14	-0.08	0.02	-0.73	0.42	-0.48	0.43	-0.25	0.07
Y144	-1.48	0.36	-1.05	0.48	1.12	0.34	-0.13	0.04	-1.54	0.44	-0.91	0.35	-0.62	0.28
A145	-1.11	0.44	-0.90	1.21	1.26	0.52	-0.09	0.02	-0.83	0.67	-0.63	0.13	-0.20	0.66
G148	-1.14	0.26	-0.48	0.32	0.49	0.23	-0.19	0.03	-1.32	0.31	-0.41	0.08	-0.90	0.26
L195	-1.20	0.25	-0.10	0.07	0.18	0.08	-0.18	0.04	-1.31	0.25	-1.24	0.25	-0.06	0.04
A205	-0.51	0.16	0.26	0.17	-0.15	0.13	-0.07	0.02	-0.47	0.24	-0.42	0.17	-0.04	0.13
D206	0.33	0.72	-9.61	1.89	9.09	1.37	-0.11	0.02	-0.31	0.83	-0.14	0.80	-0.16	0.10
F207	-0.03	0.01	-0.03	0.04	0.08	0.04	0.00	0.00	0.02	0.01	-0.03	0.01	0.05	0.01
F209	-0.16	0.06	-0.22	0.10	0.35	0.12	-0.01	0.01	-0.03	0.07	-0.10	0.04	0.07	0.04
S210	-0.03	0.01	0.10	0.06	-0.07	0.07	0.00	0.00	-0.00	0.02	-0.00	0.01	0.00	0.02
N211	-0.10	0.11	0.50	0.24	-0.47	0.21	-0.00	0.01	-0.07	0.09	-0.08	0.08	0.01	0.03

Energies are in kcal/mol.

Table S20. Free energy decomposition for the curcumin/SIK3 (**SIK3-C-KE-2**) complex on the individual residue basis, where decomposition is performed in terms of the contributions from van der Waals energy, the electrostatic interaction energy, the nonpolar solvation free energy, the polar solvation free energy, the backbone energy, and the side chain energy

Residues	ΔE_{vdW}		ΔE_{ele}		ΔG_{polar}		$\Delta G_{nonpolar}$		$\Delta G_{subtotal}$		$S\Delta G_{subtotal}$		$B\Delta G_{subtotal}$	
	Avg.	Std. Dev.	Avg.	Std. Dev.	Avg.	Std. Dev.	Avg.	Std. Dev.	Avg.	Std. Dev.	Avg.	Std. Dev.	Avg.	Std. Dev.
I72	-1.84	0.44	-0.41	0.31	0.74	0.50	-0.33	0.06	-1.83	0.41	-1.78	0.36	-0.04	0.11
G73	-0.19	0.15	0.15	0.20	0.00	0.14	-0.01	0.01	-0.05	0.11	-0.07	0.06	0.02	0.10
V80	-1.19	0.23	-0.11	0.08	0.08	0.07	-0.16	0.03	-1.38	0.26	-1.24	0.24	-0.14	0.03
A93	-0.73	0.22	-0.22	0.10	0.25	0.09	-0.06	0.02	-0.77	0.24	-0.63	0.20	-0.13	0.07
K95	-1.41	0.56	-1.41	1.10	2.26	1.08	-0.13	0.04	-0.68	0.80	-0.60	0.73	-0.09	0.12
L126	-0.71	0.30	-0.30	0.10	0.30	0.07	-0.08	0.03	-0.79	0.35	-0.75	0.30	-0.04	0.06
L140	-0.54	0.27	0.06	0.16	0.08	0.15	-0.02	0.01	-0.43	0.23	-0.38	0.22	-0.04	0.05
T142	-0.81	0.38	-1.01	0.74	0.85	0.34	-0.15	0.03	-1.13	0.65	-0.86	0.53	-0.27	0.15
Y144	-1.70	0.57	-0.78	0.41	1.22	0.38	-0.18	0.08	-1.44	0.46	-1.17	0.43	-0.27	0.18
A145	-0.93	0.39	-1.53	0.89	1.73	0.49	-0.07	0.03	-0.79	0.47	-0.46	0.14	-0.34	0.48
G148	-0.93	0.32	-0.11	0.30	0.21	0.27	-0.16	0.03	-1.00	0.39	-0.37	0.12	-0.63	0.31
L195	-1.21	0.29	-0.18	0.08	0.22	0.08	-0.22	0.03	-1.38	0.32	-1.33	0.30	-0.05	0.05
A205	-0.21	0.07	-0.09	0.06	0.09	0.06	-0.05	0.03	-0.25	0.10	-0.20	0.08	-0.05	0.03
D206	-0.23	0.08	0.12	0.61	0.44	0.58	-0.03	0.03	0.30	0.20	0.30	0.21	0.00	0.03
F207	-0.04	0.03	-0.05	0.08	0.09	0.11	-0.00	0.00	0.00	0.02	-0.04	0.01	0.05	0.02
F209	-1.24	0.43	-0.23	0.57	0.48	0.54	-0.13	0.03	-1.12	0.56	-0.90	0.43	-0.23	0.34
S210	-0.77	0.33	-0.59	1.14	0.65	0.63	-0.08	0.02	-0.79	0.57	-0.34	0.09	-0.45	0.56
N211	-0.74	0.42	-0.41	1.29	0.62	0.63	-0.09	0.03	-0.62	0.64	-0.46	0.70	-0.15	0.28

Energies are in kcal/mol.

Table S21. Free energy decomposition for the curcumin/SIK3 (**SIK3-C-KE-3**) complex on the individual residue basis, where decomposition is performed in terms of the contributions from van der Waals energy, the electrostatic interaction energy, the nonpolar solvation free energy, the polar solvation free energy, the backbone energy, and the side chain energy

Residues	ΔE_{vdW}		ΔE_{ele}		ΔG_{polar}		$\Delta G_{nonpolar}$		$\Delta G_{subtotal}$		$S\Delta G_{subtotal}$		$B\Delta G_{subtotal}$	
	Avg.	Std. Dev.	Avg.	Std. Dev.	Avg.	Std. Dev.	Avg.	Std. Dev.	Avg.	Std. Dev.	Avg.	Std. Dev.	Avg.	Std. Dev.
I72	-1.94	0.36	-0.33	0.13	0.40	0.14	-0.26	0.05	-2.13	0.38	-1.84	0.35	-0.29	0.11
G73	-0.84	0.21	0.23	0.22	0.11	0.20	-0.18	0.05	-0.68	0.23	-0.28	0.07	-0.41	0.18
V80	-1.23	0.21	-0.09	0.06	0.13	0.05	-0.20	0.03	-1.40	0.22	-1.31	0.21	-0.09	0.04
A93	-0.51	0.22	-0.33	0.12	0.33	0.09	-0.03	0.01	-0.55	0.21	-0.41	0.22	-0.14	0.07
K95	-1.78	0.30	-1.73	0.88	3.36	1.17	-0.23	0.04	-0.38	0.76	-0.40	0.73	0.02	0.06
L126	-1.28	0.23	-0.35	0.11	0.40	0.12	-0.11	0.03	-1.34	0.24	-1.32	0.23	-0.02	0.04
L140	-0.63	0.24	0.22	0.11	-0.03	0.12	-0.05	0.02	-0.49	0.22	-0.50	0.22	0.01	0.03
T142	-0.92	0.25	-0.49	0.75	0.54	0.37	-0.13	0.02	-1.01	0.46	-0.74	0.44	-0.27	0.08
Y144	-1.22	0.27	-0.79	0.34	1.11	0.26	-0.08	0.03	-0.98	0.33	-0.77	0.28	-0.21	0.15
A145	-1.24	0.33	-1.03	0.44	1.63	0.37	-0.09	0.02	-0.74	0.35	-0.61	0.15	-0.13	0.31
G148	-1.12	0.28	-0.35	0.27	0.42	0.24	-0.18	0.02	-1.24	0.33	-0.43	0.09	-0.81	0.28
L195	-1.51	0.25	-0.15	0.06	0.21	0.07	-0.23	0.03	-1.68	0.26	-1.61	0.25	-0.07	0.04
A205	-1.21	0.31	-0.37	0.34	0.76	0.31	-0.14	0.04	-0.96	0.32	-0.74	0.21	-0.22	0.24
D206	-0.77	0.23	0.09	0.87	0.01	0.84	-0.02	0.01	-0.69	0.31	-0.40	0.16	-0.29	0.24
F207	-0.08	0.58	-2.12	1.15	1.31	0.51	-0.08	0.04	-0.97	0.69	-0.23	0.10	-0.75	0.67
F209	-0.55	0.28	0.13	1.06	0.25	0.63	-0.03	0.03	-0.20	0.54	-0.26	0.16	0.06	0.48
S210	-0.22	0.13	0.05	0.22	0.00	0.15	-0.00	0.00	-0.17	0.19	-0.06	0.05	-0.10	0.16
N211	-0.51	0.28	-0.15	0.41	0.57	0.44	-0.05	0.02	-0.13	0.29	-0.17	0.29	0.03	0.06

Energies are in kcal/mol.

Table S22. Free energy decomposition for the curcumin/SIK3 (**SIK3-O-KK-1**) complex on the individual residue basis, where decomposition is performed in terms of the contributions from van der Waals energy, the electrostatic interaction energy, the nonpolar solvation free energy, the polar solvation free energy, the backbone energy, and the side chain energy

Residues	ΔE_{vdW}		ΔE_{ele}		ΔG_{polar}		$\Delta G_{nonpolar}$		$\Delta G_{subtotal}$		$S\Delta G_{subtotal}$		$B\Delta G_{subtotal}$	
	Avg.	Std. Dev.	Avg.	Std. Dev.	Avg.	Std. Dev.	Avg.	Std. Dev.	Avg.	Std. Dev.	Avg.	Std. Dev.	Avg.	Std. Dev.
I72	-1.99	0.34	-0.32	0.33	0.64	0.31	-0.37	0.06	-2.05	0.52	-2.04	0.42	-0.01	0.20
G73	-0.18	0.17	0.21	0.11	-0.08	0.14	-0.02	0.03	-0.07	0.12	-0.06	0.06	-0.01	0.09
V80	-0.76	0.29	-0.13	0.21	0.09	0.11	-0.15	0.03	-0.96	0.25	-0.81	0.28	-0.15	0.09
A93	-0.70	0.17	-0.40	0.29	0.45	0.20	-0.04	0.02	-0.70	0.22	-0.61	0.17	-0.08	0.10
V114	-0.20	0.21	-0.07	0.14	0.06	0.10	-0.01	0.02	-0.22	0.26	-0.14	0.17	-0.08	0.10
M117	-1.39	0.33	-0.04	0.28	0.30	0.22	-0.16	0.04	-1.29	0.36	-1.24	0.36	-0.05	0.04
I126	-1.69	0.37	-0.39	0.21	0.88	0.39	-0.13	0.03	-1.32	0.43	-1.39	0.36	0.07	0.17
L140	-0.33	0.30	0.05	0.23	0.01	0.26	-0.02	0.03	-0.30	0.30	-0.25	0.25	-0.04	0.06
T142	-1.00	0.30	0.03	0.66	0.27	0.49	-0.11	0.03	-0.81	0.39	-0.68	0.32	-0.13	0.08
Y144	-1.10	0.43	-2.03	0.35	1.46	0.24	-0.08	0.04	-1.75	0.35	-0.90	0.29	-0.86	0.24
A145	-1.02	0.42	-1.70	0.57	1.56	0.35	-0.08	0.03	-1.24	0.37	-0.64	0.16	-0.60	0.34
G148	-0.86	0.27	-0.10	0.25	0.13	0.23	-0.14	0.03	-0.98	0.31	-0.32	0.09	-0.65	0.25
E149	-0.77	0.66	-4.32	2.86	5.33	2.73	-0.22	0.05	0.03	0.74	0.21	0.78	-0.18	0.12
L195	-1.47	0.33	-0.17	0.14	0.20	0.16	-0.25	0.03	-1.70	0.33	-1.64	0.33	-0.06	0.03
A205	-1.31	0.32	-0.30	0.50	0.88	0.84	-0.12	0.06	-0.85	0.69	-0.78	0.27	-0.07	0.49
F207	-0.81	0.39	-0.41	0.63	0.36	0.57	-0.04	0.02	-0.91	0.40	-0.68	0.35	-0.23	0.25

Energies are in kcal/mol.

Table S23. Free energy decomposition for the curcumin/SIK3 (**SIK3-O-KK-2**) complex on the individual residue basis, where decomposition is performed in terms of the contributions from van der Waals energy, the electrostatic interaction energy, the nonpolar solvation free energy, the polar solvation free energy, the backbone energy, and the side chain energy

Residues	ΔE_{vdW}		ΔE_{ele}		ΔG_{polar}		$\Delta G_{nonpolar}$		$\Delta G_{subtotal}$		$S\Delta G_{subtotal}$		$B\Delta G_{subtotal}$	
	Avg.	Std. Dev.	Avg.	Std. Dev.	Avg.	Std. Dev.	Avg.	Std. Dev.	Avg.	Std. Dev.	Avg.	Std. Dev.	Avg.	Std. Dev.
I72	-1.49	0.52	-0.27	0.33	0.65	0.39	-0.19	0.09	-1.30	0.49	-1.26	0.44	-0.04	0.23
G73	-0.76	0.38	-0.23	0.37	0.58	0.39	-0.15	0.07	-0.57	0.37	-0.31	0.15	-0.27	0.27
V80	-1.12	0.32	0.16	0.07	-0.01	0.07	-0.22	0.06	-1.18	0.33	-1.17	0.32	-0.01	0.03
A93	-0.61	0.14	-0.11	0.13	0.29	0.13	-0.02	0.01	-0.46	0.13	-0.49	0.14	0.04	0.03
V114	-0.44	0.13	-0.20	0.12	0.14	0.09	-0.02	0.01	-0.52	0.18	-0.32	0.11	-0.20	0.10
M117	-1.48	0.38	0.07	0.30	0.18	0.20	-0.13	0.02	-1.38	0.40	-1.31	0.39	-0.06	0.05
I126	-1.37	0.34	-0.43	0.14	0.52	0.16	-0.08	0.03	-1.36	0.33	-1.32	0.33	-0.03	0.05
L140	-0.72	0.23	-0.31	0.12	0.43	0.13	-0.06	0.02	-0.66	0.23	-0.52	0.23	-0.14	0.06
T142	-1.28	0.26	-0.84	0.22	1.04	0.19	-0.14	0.02	-1.23	0.26	-1.02	0.25	-0.21	0.05
Y144	-0.90	0.38	-2.00	0.34	1.27	0.17	-0.03	0.04	-1.67	0.30	-0.72	0.25	-0.95	0.20
A145	-1.06	0.37	-2.16	0.48	1.81	0.28	-0.09	0.03	-1.50	0.35	-0.72	0.18	-0.78	0.28
G148	-0.55	0.24	0.54	0.40	-0.40	0.30	-0.10	0.03	-0.51	0.37	-0.22	0.07	-0.29	0.33
E149	-0.17	0.74	-6.50	3.28	6.22	2.58	-0.12	0.06	-0.57	0.95	-0.43	0.95	-0.15	0.09
L195	-1.71	0.38	-0.34	0.12	0.39	0.09	-0.26	0.03	-1.92	0.39	-1.84	0.38	-0.08	0.03
A205	-1.09	0.31	-0.69	0.51	1.90	0.44	-0.16	0.03	-0.05	0.37	-0.56	0.15	0.51	0.39
F207	-0.60	0.24	0.15	0.23	-0.22	0.17	-0.02	0.01	-0.70	0.33	-0.48	0.28	-0.22	0.22

Energies are in kcal/mol.

Table S24. Free energy decomposition for the curcumin/SIK3 (**SIK3-O-KK-3**) complex on the individual residue basis, where decomposition is performed in terms of the contributions from van der Waals energy, the electrostatic interaction energy, the nonpolar solvation free energy, the polar solvation free energy, the backbone energy, and the side chain energy

Residues	ΔE_{vdW}		ΔE_{ele}		ΔG_{polar}		$\Delta G_{nonpolar}$		$\Delta G_{subtotal}$		$S\Delta G_{subtotal}$		$B\Delta G_{subtotal}$	
	Avg.	Std. Dev.	Avg.	Std. Dev.	Avg.	Std. Dev.	Avg.	Std. Dev.	Avg.	Std. Dev.	Avg.	Std. Dev.	Avg.	Std. Dev.
I72	-1.90	0.41	-0.13	0.22	0.62	0.44	-0.38	0.04	-1.80	0.40	-1.87	0.34	0.07	0.20
G73	-0.15	0.10	0.06	0.07	0.03	0.06	-0.02	0.03	-0.08	0.08	-0.05	0.04	-0.03	0.05
V80	-1.14	0.22	0.10	0.06	-0.03	0.05	-0.17	0.03	-1.24	0.25	-1.19	0.23	-0.04	0.04
A93	-0.75	0.17	0.15	0.14	0.13	0.16	-0.04	0.01	-0.52	0.17	-0.56	0.17	0.05	0.05
V114	-0.23	0.11	-0.05	0.08	0.05	0.07	-0.01	0.01	-0.24	0.13	-0.17	0.10	-0.07	0.05
M117	-1.15	0.32	-0.13	0.24	0.25	0.16	-0.12	0.03	-1.15	0.36	-1.10	0.36	-0.05	0.03
I126	-1.39	0.28	-0.30	0.09	0.33	0.11	-0.09	0.02	-1.45	0.29	-1.38	0.28	-0.07	0.05
L140	-0.79	0.19	-0.46	0.17	0.49	0.15	-0.05	0.02	-0.80	0.21	-0.56	0.18	-0.24	0.11
T142	-1.22	0.36	-0.90	0.25	1.09	0.18	-0.13	0.03	-1.17	0.37	-0.91	0.37	-0.26	0.06
Y144	-1.72	0.43	-1.66	0.35	1.41	0.23	-0.14	0.04	-2.11	0.36	-1.22	0.31	-0.89	0.21
A145	-0.72	0.42	-2.16	0.46	1.78	0.27	-0.05	0.02	-1.14	0.41	-0.53	0.11	-0.62	0.40
G148	-0.85	0.25	-0.17	0.22	0.22	0.18	-0.14	0.03	-0.94	0.25	-0.31	0.08	-0.63	0.20
E149	-0.99	0.49	-2.09	2.16	3.24	2.16	-0.23	0.06	-0.07	0.52	0.04	0.51	-0.10	0.07
L195	-1.50	0.35	-0.37	0.11	0.41	0.08	-0.25	0.03	-1.71	0.35	-1.63	0.35	-0.08	0.03
A205	-1.06	0.26	-1.00	0.42	1.92	0.39	-0.19	0.03	-0.33	0.29	-0.51	0.18	0.18	0.28
F207	-0.51	0.15	0.34	0.13	-0.26	0.13	-0.01	0.01	-0.44	0.20	-0.32	0.14	-0.12	0.17

Energies are in kcal/mol.

Table S25. Free energy decomposition for the curcumin/SIK3 (**SIK3-O-KE-1**) complex on the individual residue basis, where decomposition is performed in terms of the contributions from van der Waals energy, the electrostatic interaction energy, the nonpolar solvation free energy, the polar solvation free energy, the backbone energy, and the side chain energy

Residues	ΔE_{vdW}		ΔE_{ele}		ΔG_{polar}		$\Delta G_{nonpolar}$		$\Delta G_{subtotal}$		$S\Delta G_{subtotal}$		$B\Delta G_{subtotal}$	
	Avg.	Std. Dev.	Avg.	Std. Dev.	Avg.	Std. Dev.	Avg.	Std. Dev.	Avg.	Std. Dev.	Avg.	Std. Dev.	Avg.	Std. Dev.
I72	-1.78	0.42	-0.30	0.18	0.38	0.28	-0.28	0.05	-1.97	0.40	-1.78	0.35	-0.19	0.17
V80	-0.65	0.33	-0.08	0.05	0.08	0.05	-0.15	0.06	-0.80	0.38	-0.72	0.36	-0.08	0.04
A93	-0.57	0.19	-0.17	0.10	0.18	0.06	-0.04	0.02	-0.60	0.19	-0.49	0.18	-0.11	0.06
E113	0.71	0.86	-8.82	2.01	6.49	1.40	-0.03	0.02	-1.65	0.96	-1.65	0.96	-0.01	0.03
M117	-1.48	0.33	-0.23	0.28	0.50	0.23	-0.18	0.04	-1.39	0.37	-1.33	0.37	-0.05	0.04
I126	-1.68	0.39	-0.69	0.24	1.10	0.28	-0.14	0.03	-1.41	0.44	-1.34	0.39	-0.07	0.16
T142	-0.82	0.28	-0.27	0.58	0.41	0.31	-0.09	0.02	-0.78	0.39	-0.55	0.38	-0.23	0.07
Y144	-1.61	0.29	-1.38	0.37	1.33	0.29	-0.12	0.04	-1.78	0.35	-1.13	0.31	-0.65	0.18
A145	-1.43	0.30	-1.49	0.50	1.80	0.31	-0.09	0.02	-1.20	0.36	-0.68	0.13	-0.52	0.34
G148	-1.09	0.28	-0.15	0.34	0.15	0.33	-0.17	0.02	-1.27	0.36	-0.40	0.10	-0.87	0.30
L195	-1.31	0.24	-0.13	0.06	0.14	0.07	-0.20	0.03	-1.49	0.24	-1.39	0.24	-0.10	0.04
I204	-0.31	0.08	-0.31	0.14	0.24	0.13	-0.00	0.00	-0.38	0.12	-0.09	0.02	-0.29	0.11
A205	-1.38	0.25	-0.46	0.18	0.60	0.18	-0.09	0.03	-1.33	0.34	-0.92	0.20	-0.41	0.21
D206	-1.52	0.28	-1.31	0.55	1.89	0.99	-0.14	0.04	-1.07	0.79	-0.31	0.61	-0.76	0.41
F207	-0.91	0.44	-1.20	0.42	1.01	0.22	-0.06	0.03	-1.16	0.38	-0.93	0.24	-0.23	0.31

Energies are in kcal/mol.

Table S26. Free energy decomposition for the curcumin/SIK3 (**SIK3-O-KE-2**) complex on the individual residue basis, where decomposition is performed in terms of the contributions from van der Waals energy, the electrostatic interaction energy, the nonpolar solvation free energy, the polar solvation free energy, the backbone energy, and the side chain energy

Residues	ΔE_{vdW}		ΔE_{ele}		ΔG_{polar}		$\Delta G_{nonpolar}$		$\Delta G_{subtotal}$		$S\Delta G_{subtotal}$		$B\Delta G_{subtotal}$	
	Avg.	Std. Dev.	Avg.	Std. Dev.	Avg.	Std. Dev.	Avg.	Std. Dev.	Avg.	Std. Dev.	Avg.	Std. Dev.	Avg.	Std. Dev.
I72	-1.98	0.50	-0.48	0.39	1.04	0.71	-0.37	0.05	-1.80	0.42	-1.85	0.37	0.05	0.23
V80	-0.69	0.27	-0.10	0.05	0.06	0.05	-0.15	0.05	-0.87	0.33	-0.76	0.29	-0.12	0.05
A93	-0.60	0.19	-0.15	0.10	0.15	0.07	-0.04	0.02	-0.63	0.18	-0.50	0.19	-0.13	0.06
E113	0.56	0.83	-8.49	2.49	6.53	1.75	-0.04	0.02	-1.43	0.95	-1.42	0.95	-0.01	0.04
M117	-1.44	0.33	-0.28	0.30	0.52	0.23	-0.17	0.04	-1.37	0.35	-1.31	0.34	-0.06	0.05
I126	-1.64	0.40	-0.70	0.31	1.08	0.38	-0.14	0.03	-1.40	0.46	-1.34	0.39	-0.06	0.21
T142	-0.87	0.30	-0.44	0.57	0.47	0.33	-0.11	0.03	-0.94	0.40	-0.66	0.39	-0.28	0.08
Y144	-1.51	0.30	-1.29	0.30	1.14	0.22	-0.10	0.03	-1.76	0.33	-1.05	0.27	-0.71	0.19
A145	-1.29	0.35	-1.13	0.47	1.57	0.27	-0.08	0.02	-0.93	0.43	-0.68	0.17	-0.26	0.40
G148	-1.02	0.22	-0.17	0.26	0.24	0.18	-0.16	0.03	-1.11	0.25	-0.37	0.08	-0.74	0.21
L195	-1.41	0.28	-0.10	0.06	0.14	0.07	-0.23	0.04	-1.61	0.29	-1.53	0.29	-0.08	0.03
I204	-0.37	0.12	-0.30	0.16	0.16	0.15	-0.00	0.00	-0.51	0.16	-0.13	0.06	-0.38	0.13
A205	-1.77	0.29	0.09	0.33	1.31	0.34	-0.19	0.03	-0.55	0.57	-0.72	0.18	0.17	0.52
D206	-1.28	0.26	-1.30	0.64	1.29	0.77	-0.06	0.03	-1.35	0.45	-0.52	0.27	-0.83	0.40
F207	-0.94	0.41	-0.42	0.64	0.47	0.33	-0.06	0.02	-0.95	0.53	-0.84	0.30	-0.11	0.39

Energies are in kcal/mol.

Table S27. Free energy decomposition for the curcumin/SIK3 (**SIK3-O-KE-3**) complex on the individual residue basis, where decomposition is performed in terms of the contributions from van der Waals energy, the electrostatic interaction energy, the nonpolar solvation free energy, the polar solvation free energy, the backbone energy, and the side chain energy

Residues	ΔE_{vdW}		ΔE_{ele}		ΔG_{polar}		$\Delta G_{nonpolar}$		$\Delta G_{subtotal}$		$S\Delta G_{subtotal}$		$B\Delta G_{subtotal}$	
	Avg.	Std. Dev.	Avg.	Std. Dev.	Avg.	Std. Dev.	Avg.	Std. Dev.	Avg.	Std. Dev.	Avg.	Std. Dev.	Avg.	Std. Dev.
I72	-1.99	0.45	-0.43	0.39	1.02	0.61	-0.36	0.05	-1.75	0.40	-1.84	0.35	0.09	0.24
V80	-0.84	0.36	-0.07	0.08	0.07	0.07	-0.17	0.06	-1.01	0.39	-0.89	0.36	-0.12	0.04
A93	-0.69	0.21	-0.12	0.12	0.16	0.09	-0.05	0.02	-0.69	0.21	-0.58	0.20	-0.11	0.06
E113	0.13	0.78	-6.85	2.99	6.03	2.04	-0.07	0.04	-0.76	1.25	-0.73	1.27	-0.03	0.05
M117	-0.85	0.63	-0.06	0.32	0.21	0.31	-0.10	0.06	-0.81	0.64	-0.78	0.59	-0.02	0.06
I126	-1.27	0.47	-0.48	0.28	0.71	0.42	-0.08	0.04	-1.12	0.40	-1.08	0.38	-0.04	0.14
T142	-0.95	0.37	-0.50	0.60	0.64	0.35	-0.15	0.04	-0.96	0.45	-0.68	0.45	-0.28	0.08
Y144	-1.61	0.34	-1.24	0.31	1.12	0.22	-0.10	0.03	-1.83	0.36	-1.04	0.29	-0.78	0.20
A145	-1.13	0.41	-1.32	0.47	1.54	0.26	-0.08	0.02	-0.98	0.42	-0.66	0.14	-0.31	0.42
G148	-1.04	0.25	-0.18	0.26	0.25	0.17	-0.17	0.03	-1.14	0.26	-0.37	0.09	-0.77	0.22
L195	-1.46	0.29	-0.11	0.07	0.16	0.08	-0.24	0.03	-1.64	0.29	-1.56	0.29	-0.08	0.03
I204	-0.27	0.19	-0.19	0.23	0.09	0.22	-0.00	0.00	-0.36	0.23	-0.11	0.08	-0.26	0.17
A205	-1.61	0.37	-0.10	0.44	1.28	0.37	-0.20	0.03	-0.63	0.60	-0.74	0.19	0.10	0.53
D206	-1.14	0.41	-0.72	1.13	1.03	0.94	-0.06	0.04	-0.89	0.58	-0.37	0.36	-0.52	0.37
F207	-0.61	0.43	0.15	0.43	-0.05	0.37	-0.03	0.03	-0.54	0.42	-0.49	0.37	-0.04	0.28

Energies are in kcal/mol.
